# Supplementary material for: Myriapod genomes reveal ancestral horizontal gene transfer and hormonal gene loss in millipedes
Source: Nat Commun. 2022 May 30;13:3010. doi: 10.1038/s41467-022-30690-0 (PMC9151784; doi:10.1038/s41467-022-30690-0)
Supplement: Supplementary file 1 — Supplementary Information [file 41467_2022_30690_MOESM1_ESM.pdf]

Supplementary Information

Myriapod genomes reveal ancestral horizontal gene transfer and hormonal gene loss in millipedes

So, Nong, and Xie et al

Supplementary Figures

Figure 1. Macrosynteny between *Thereuonema tuberculata* (centipede) and eight other myriapods.

Figure 2. Macrosynteny between *Trigoniulus corallinus* (millipede) and eight other myriapods.

Figure 3. Macrosynteny between *Helicorthomorpha holstii* (millipede) and eight other myriapods.

Figure 4. Number of shared orthogroups among 9 myriapods and 24 outgroup species.

Figure 5. GO enrichment analysis of conserved gene families.

Figure 6. KEGG enrichment analysis of conserved gene families.

Figure 7. KOG enrichment analysis of conserved gene families.

Figure 8. Schematic summary of Hox and ParaHox gene arrangement in the genome of centipede *R. immarginata*.

Figure 9. Cladogram of HOXL genes

Figure 10. Phylogenetic tree of glucose/arabinose dehydrogenase (GDH)

Figure 11. Phylogenetic tree of non-ribosomal peptide synthetase (NRPS)

Figure 12. Phylogenetic tree of glycoside hydrolase family 16 protein (GH16)

Figure 13. Phylogenetic tree of efflux RND transporter permease subunit

Figure 14. Phylogenetic tree of NADH dehydrogenase

Figure 15. Phylogenetic tree of AzlD domain-containing protein

Figure 16. Phylogenetic tree of anaerobic sulfatase maturase

Figure 17. Phylogenetic tree of alpha-2-macroglobulin

Figure 18. Phylogenetic tree of SYLF domain-containing protein

Figure 19. Microsynteny of glucose/arabinose dehydrogenase (GDH; WP_189008864.1)

Figure 20. Microsynteny of glucose/arabinose dehydrogenase (GDH; WP_146884959.1)

Figure 21. Microsynteny of non-ribosomal peptide synthetase (NRPS; WP_096595152.1)

Figure 22. Microsynteny of glycoside hydrolase family 16 protein (GH16; AQQ75061.1)

Figure 23. Microsynteny of glycoside hydrolase family 16 protein (GH16; WP_052600908.1)

Figure 24. Microsynteny of efflux RND transporter permease subunit (WP_163176792.1)

Figure 25. Microsynteny of NADH dehydrogenase (WP_034862301.1)

Figure 26. Microsynteny of AzlD domain-containing protein (WP_095524423.1)

Figure 27. Microsynteny of anaerobic sulfatase maturase (SBW02910.1)

Figure 28. Microsynteny of alpha-2-macroglobulin (OYY43986.1)

Figure 29. Microsynteny of SYLF domain-containing protein (RPH48231.1)

Figure 30. Tissue differential expression of horizontal transferred WP_189008864.1 Glucose/arabinose dehydrogenases in *T. tuberculata.*

Figure 31. Phylogenetic tree of acetyl-CoA C-acetyltransferase (ACAT)

Figure 32. Phylogenetic tree of hydroxymethylglutaryl-CoA synthase (HMGCS)

Figure 33. Phylogenetic tree of hydroxymethylglutaryl-CoA reductase (HMGCR)

Figure 34. Phylogenetic tree of mevalonate kinase (MK) and phosphomevalonate kinase (PMK)

Figure 35. Phylogenetic tree of diphosphomevalonate decarboxylase (DPMD)

Figure 36. Phylogenetic tree of isopentenyl-diphosphate delta-isomerase (IPPI)

Figure 37. Phylogenetic tree of farnesyl pyrophosphate synthase (FPPS)

Figure 38. Phylogenetic tree of farnesyltransferase beta (FNTB)

Figure 39. Phylogenetic tree of ste24 endopeptidase (ste24)

Figure 40. Phylogenetic tree of protein-S-isoprenylcysteine O-methyltransferase (ste14/ICMT)

Figure 41. Phylogenetic tree of prenylcysteine oxidase (PCYOX1)

Figure 42. Phylogenetic tree of aldehyde dehydrogenase 3 (ALDH3)

Figure 43. Phylogenetic tree of juvenile hormone acid methyltransferase (JHAMT)

Figure 44. Phylogenetic tree of juvenile hormone epoxide hydrolase (JHEH)

Figure 45. Phylogenetic tree of juvenile hormone diol kinase (JHDK)

Figure 46. Phylogenetic tree of cytosolic juvenile hormone binding protein (cJHBP)/glyoxalase domain containing protein 4 (GLOD4)

Figure 47. Phylogenetic tree of ultraspiracle (USP) and ecdysteroid receptor (EcR)

Figure 48. Phylogenetic tree of steroid receptor coactivator (SRC)

Figure 49. Phylogenetic tree of methoprene receptor (Met)

Figure 50. Phylogenetic tree of heat shock protein 83 (Hsp83)

Figure 51. Phylogenetic tree of allatotropin receptor (ATR)

Figure 52. Phylogenetic tree of allatostatin receptor (ASR)

Figure 53. Phylogenetic tree of S-adenosylmethionine synthase (MAT)

Figure 54. Phylogenetic tree of S-adenosylhomocysteinase (AHC)

Figure 55. Phylogenetic tree of adenosine kinase (AK)

Figure 56. Sequence alignment of centipede juvenile hormone acid methyltransferase (JHAMT)

Figure 57. Microsynteny of JHAMT in centipede genomes

Figure 58. Hormone measurement of sesquiterpenoids

Figure 59. Myriapod repeat location plots

Figure 60. Myriapod repeat landscape plots

Figure 61. Repeat density karyotype plot for *Helicorthomorpha holstii*

Figure 62. Repeat density karyotype plot for *Trigoniulus corallinus*

Figure 63. Repeat density karyotype plot for *Thereuonema tuberculata*

Figure 64. Bar chart showing the pattern by which different myriapod species share individual TE families

Supplementary Discussion - Genome size and transposable element content

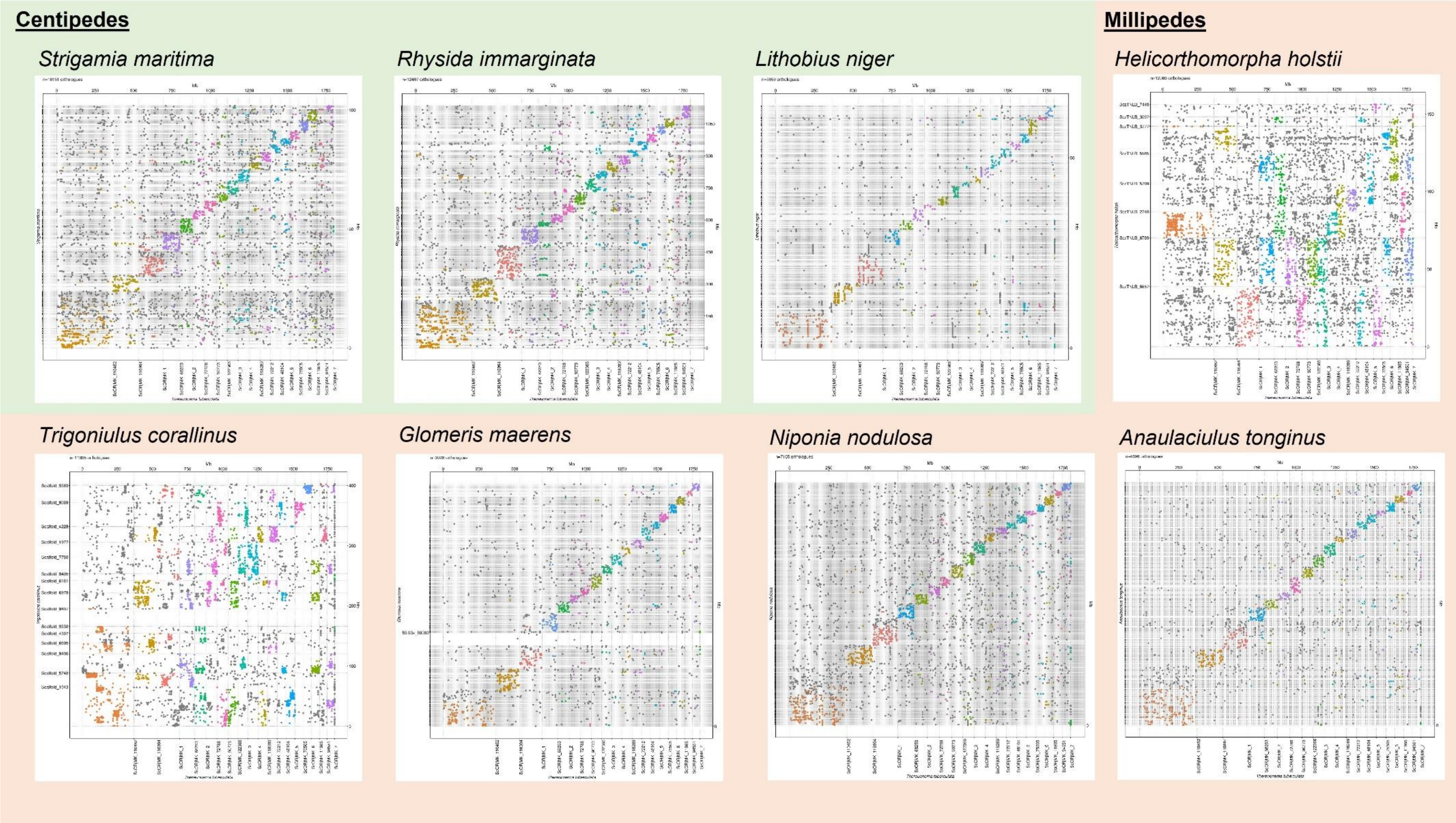

Supplementary Figure 1. Macrosynteny between *Trigoniulus corallinus* (millipede) and eight other myriapods.

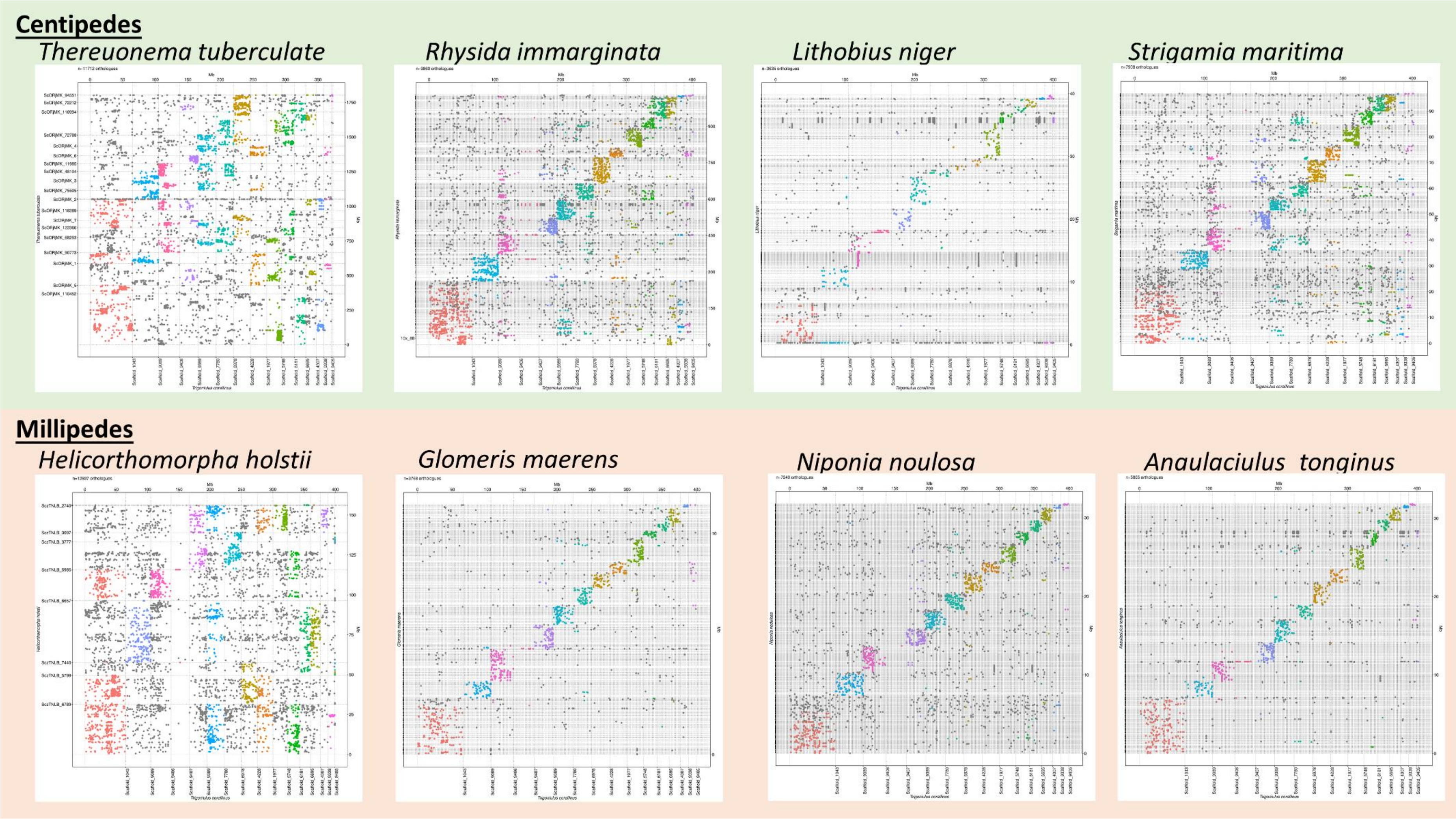

Supplementary Figure 2. Macrosynteny between *Trigoniulus corallinus* (millipede) and eight other myriapods.

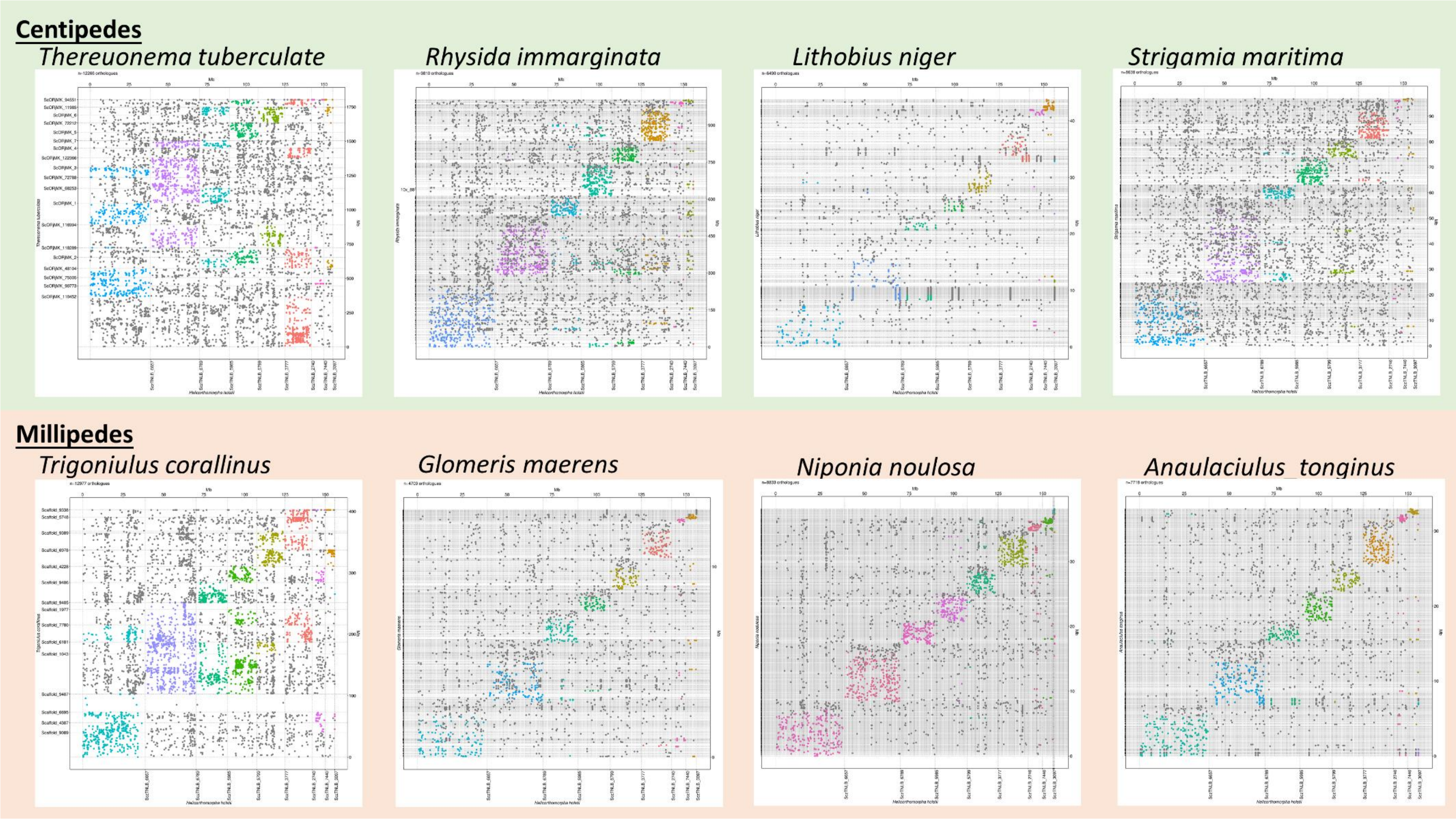

Supplementary Figure 3. Macrosynteny between *Helicorthomorpha holstii* (millipede) and eight other myriapods.

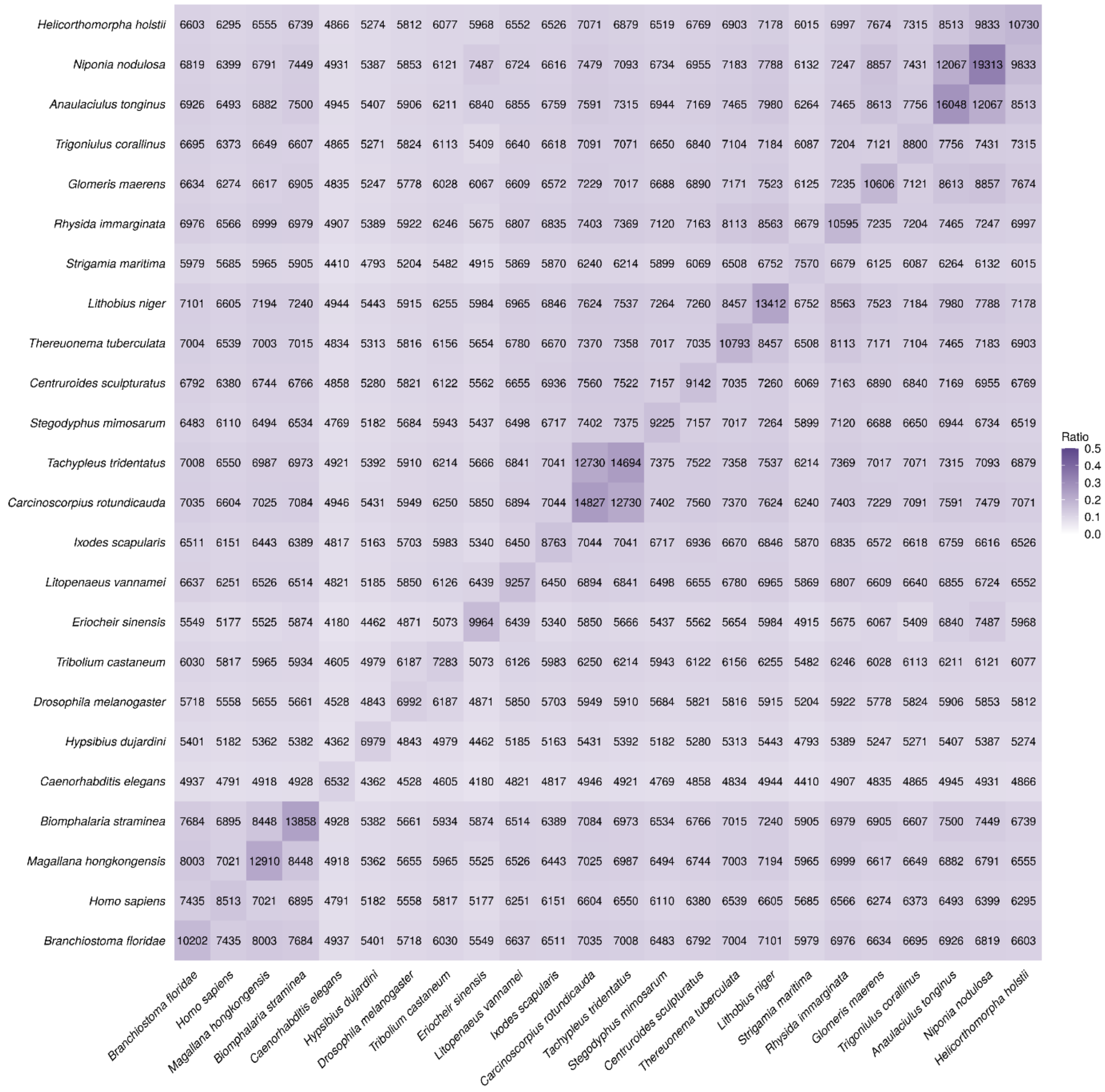

Supplementary Figure 4. Number of shared orthogroups among 9 myriapods and 24 outgroup species.

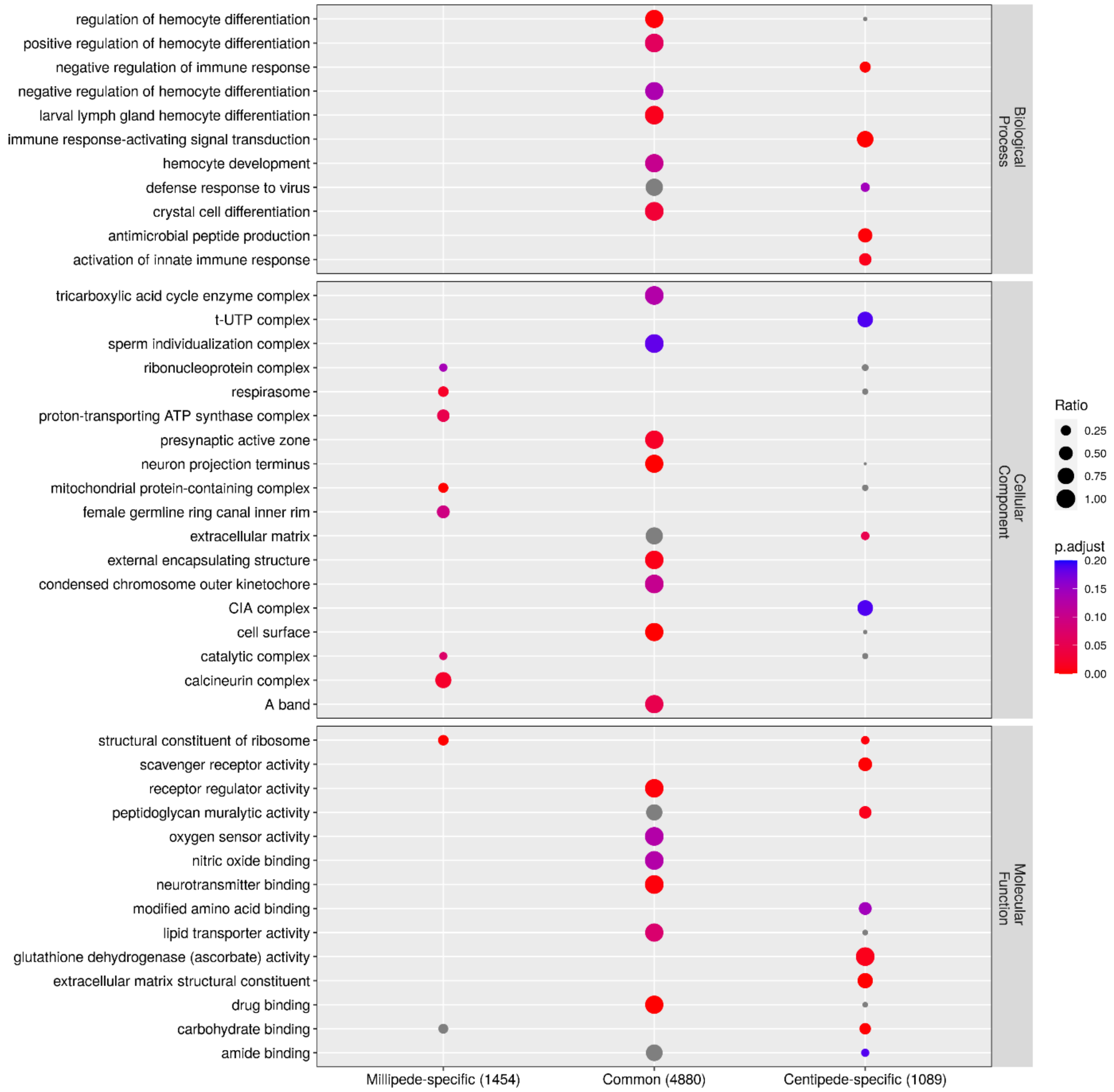

Supplementary Figure 5. GO enrichment analysis of conserved gene families. The number of annotated gene families in each group are listed below. Enrich groups with Benjamini and Hochberg method (BH) adjusted p value ≤ 0.20 are coloured red to blue, others are in grey. Ratio are calculated by number of gene families over total number of annotated gene families of specific GO term. Top 20 enriched groups are shown.

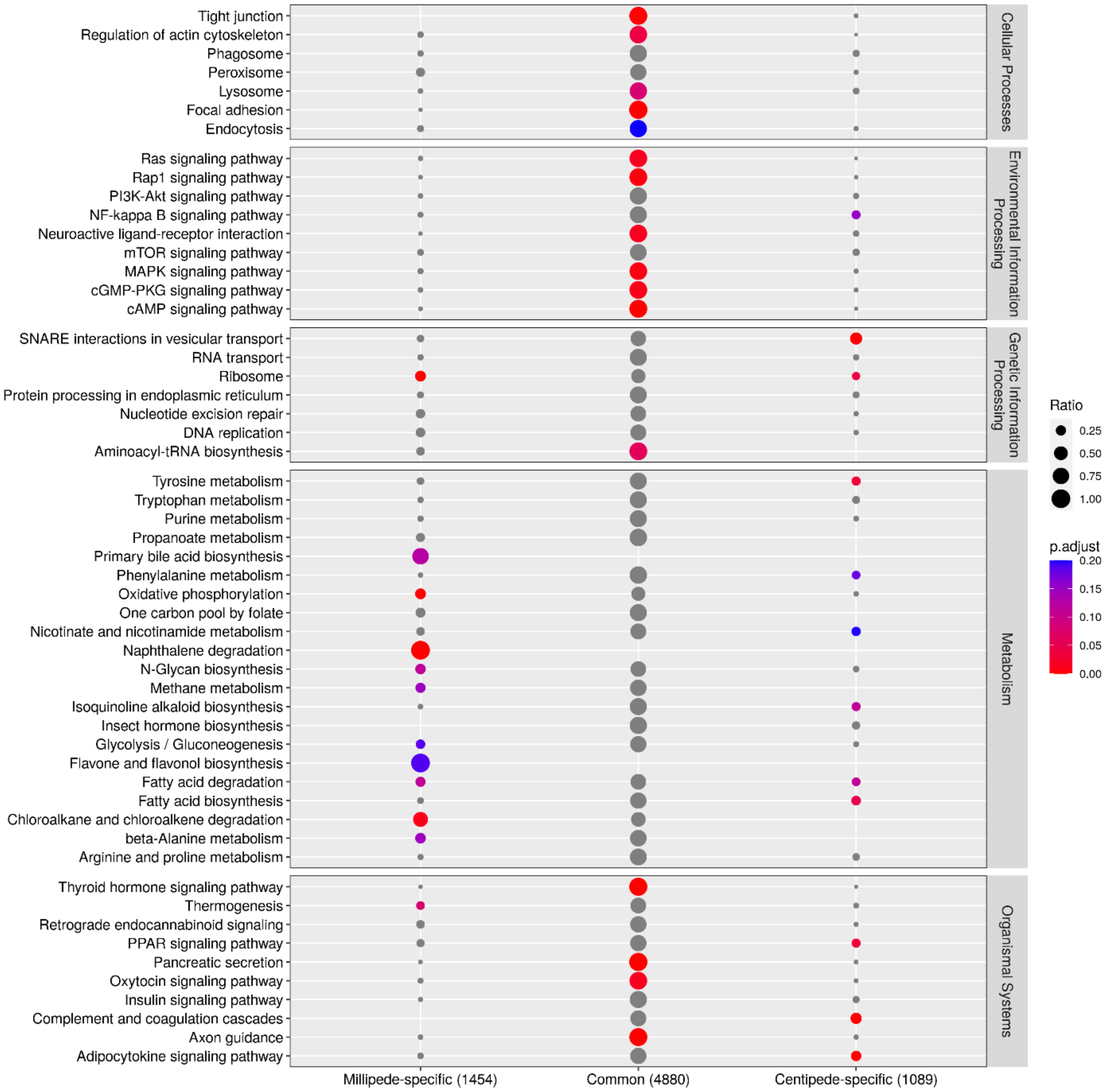

Supplementary Figure 6. KEGG enrichment analysis of conserved gene families. The number of annotated gene families in each group are listed below. Enrich groups with Benjamini and Hochberg method (BH) adjusted p value ≤ 0.20 are coloured red to blue, others are in grey. Ratio are calculated by number of gene families over total number of annotated gene families of specific KEGG term. Top 20 enriched groups are shown.

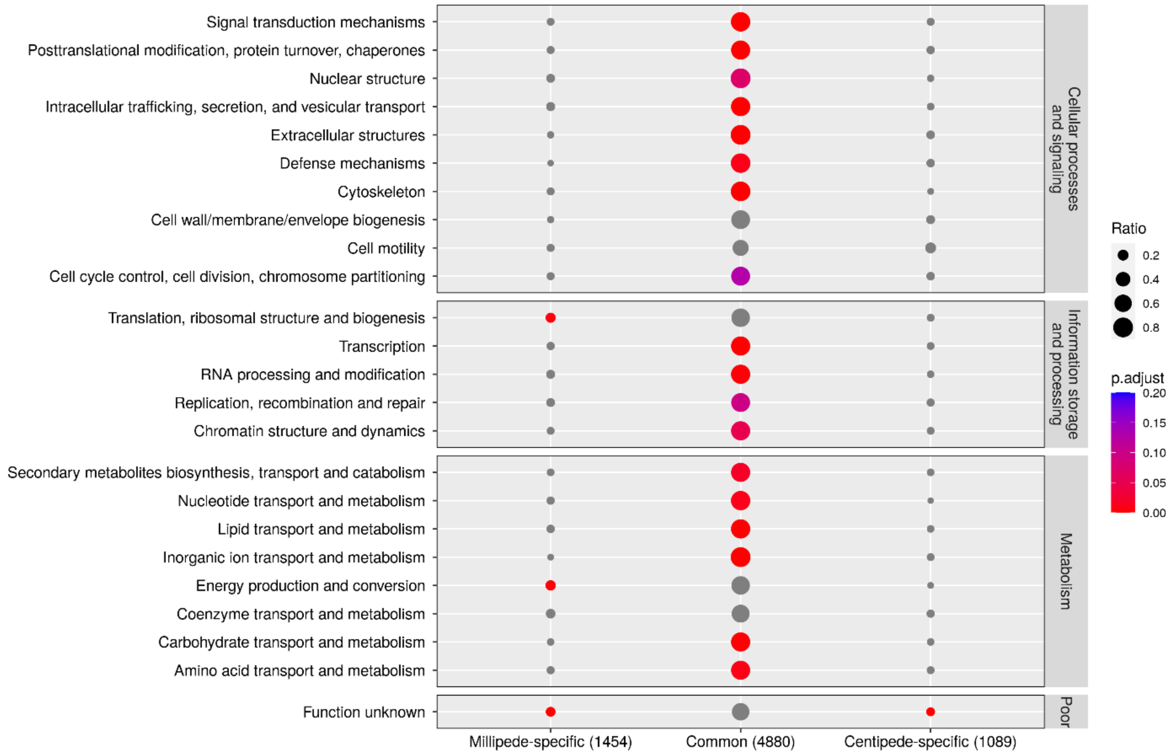

Supplementary Figure 7. KOG enrichment analysis of conserved gene families. The number of annotated gene families in each group are listed below. Enrich groups with Benjamini and Hochberg method (BH) adjusted p value ≤ 0.20 are coloured red to blue, others are in grey. Ratio are calculated by number of gene families over total number of annotated gene families of specific KOG term.

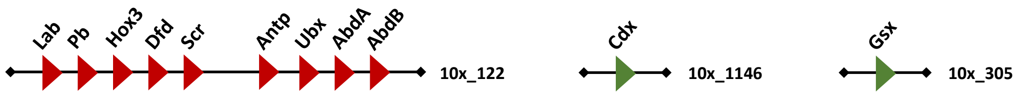

Supplementary Figure 8. Schematic summary of Hox and ParaHox gene arrangement in the genome of centipede *R. immarginata*.

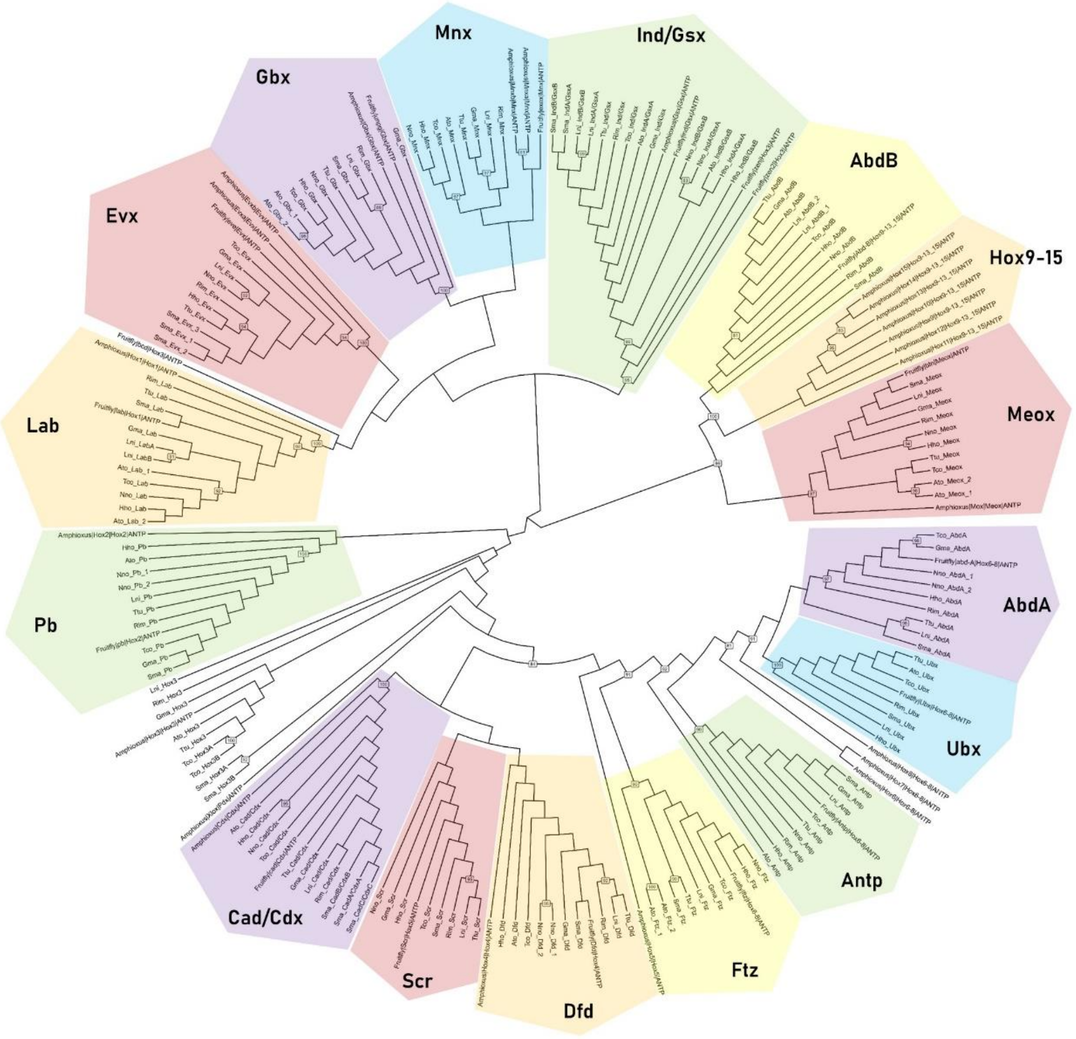

Supplementary Figure 9. Cladogram of HOXL genes. The phylogenetic tree was constructed with the maximum likelihood method in IQ-Tree with ultrafast bootstrap of 1000 times (-B 1000 -bnni --alrt 1000). LG+I+G4 was chosen as the best-fit model according to BIC value. The tree was midpoint rooted using function ‘midpoint()’ in R package ‘phangorn’ v2.7.1. The phylogenetic tree was visualized using function ‘ggtree()’ in R package ‘ggtree’ v3.0.2, with ‘branch.length = "none"’ to show the cladogram. Only bootstrap values larger than 80% are indicated for clarity. Bilaterian homeobox genes from *Branchiostoma floridae* and *Drosophila melanogaster* are used as bilaterian representatives. Ttu, *T. tuberculate*; Rim, *R. immarginata*; Lni, *L. niger;* Sma, *S. maritima*; Gma, *G. maerens*; Hho, *H. holstii*, Nno, *N. niponia*, Tco, *T. corallinus*, Ato, *A. tonginus*.

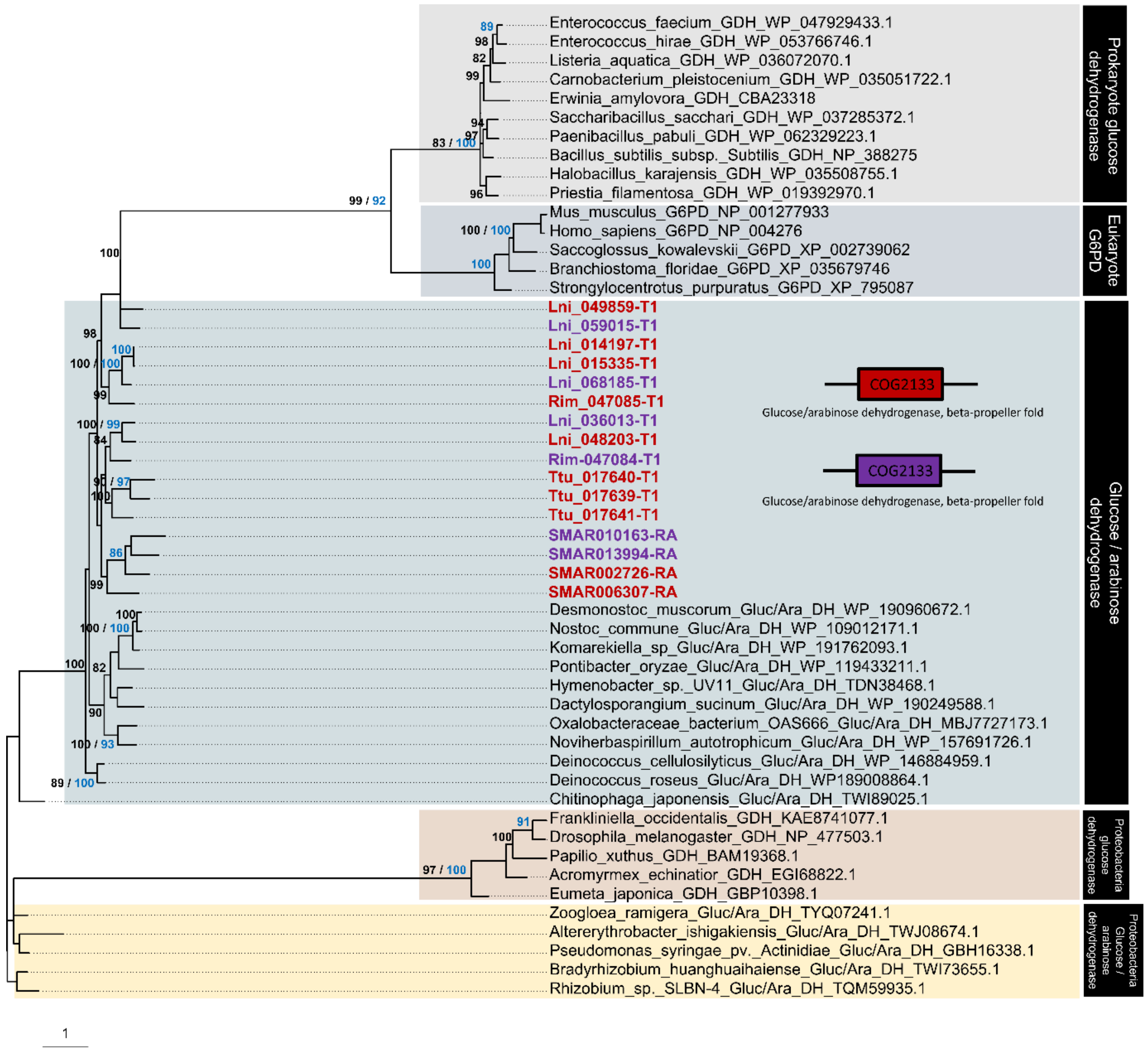

Supplementary Figure 10. Phylogenetic tree of glucose/arabinose dehydrogenase (GDH). Tree topology is displayed according to the Maximum Likelihood (ML) method (WAG+G4). Bootstrapping was conducted on each node with 1000 replicates on both ML (black) and NJ (blue) algorithms. Only bootstrap values larger than 80% are indicated for clarity. Ttu, *T. tuberculate*; Rim, *R. immarginata*; Lni, *L. niger*; SMAR, *S. maritima*.

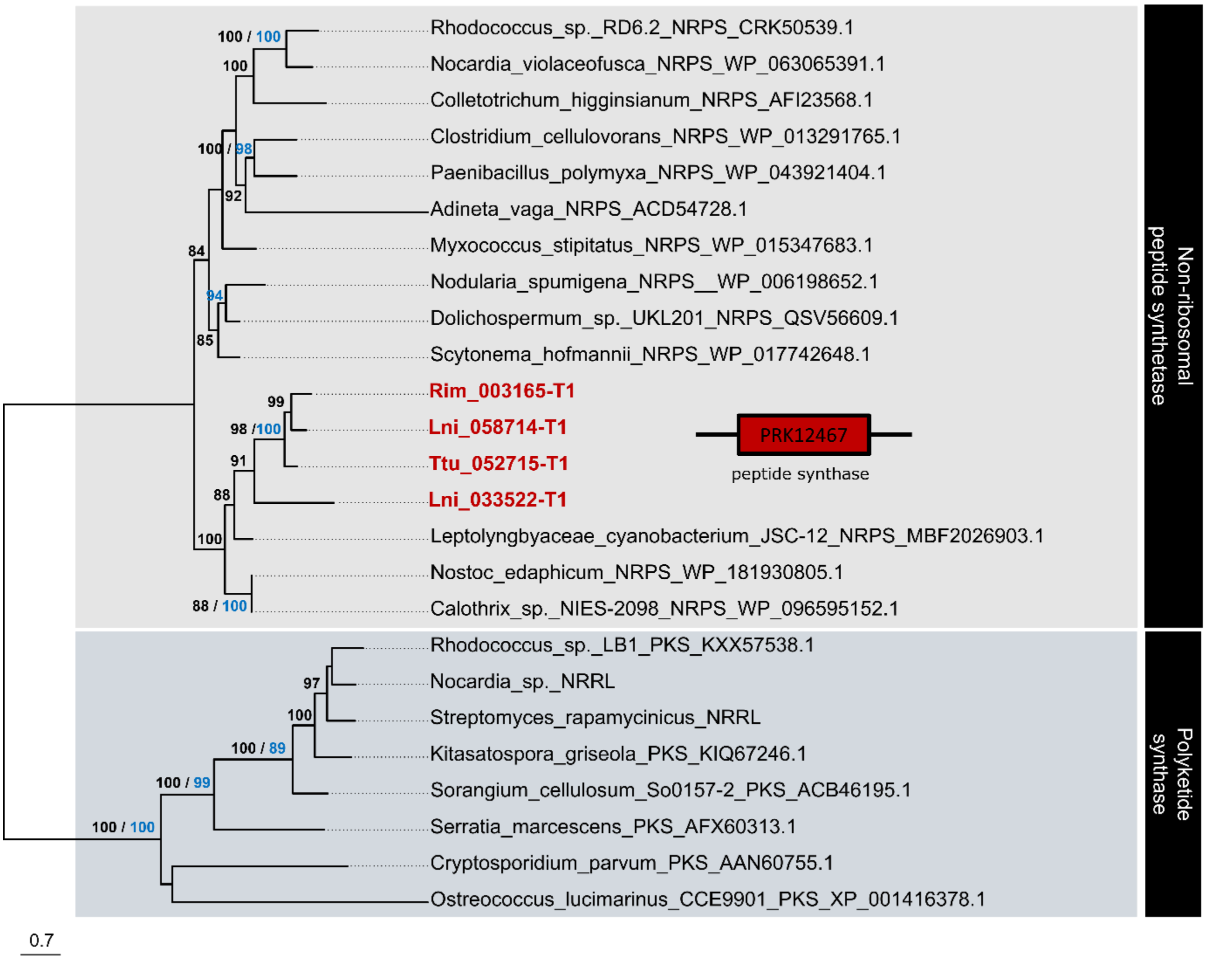

Supplementary Figure 11. Phylogenetic tree of non-ribosomal peptide synthetase (NRPS). Tree topology is displayed according to the Maximum Likelihood (ML) method (WAG+G4). Bootstrapping was conducted on each node with 1000 replicates on both ML (black) and NJ (blue) algorithms. Only bootstrap values larger than 80% are indicated for clarity. Ttu, *T. tuberculate*; Rim, *R. immarginata*; Lni, *L. niger*.

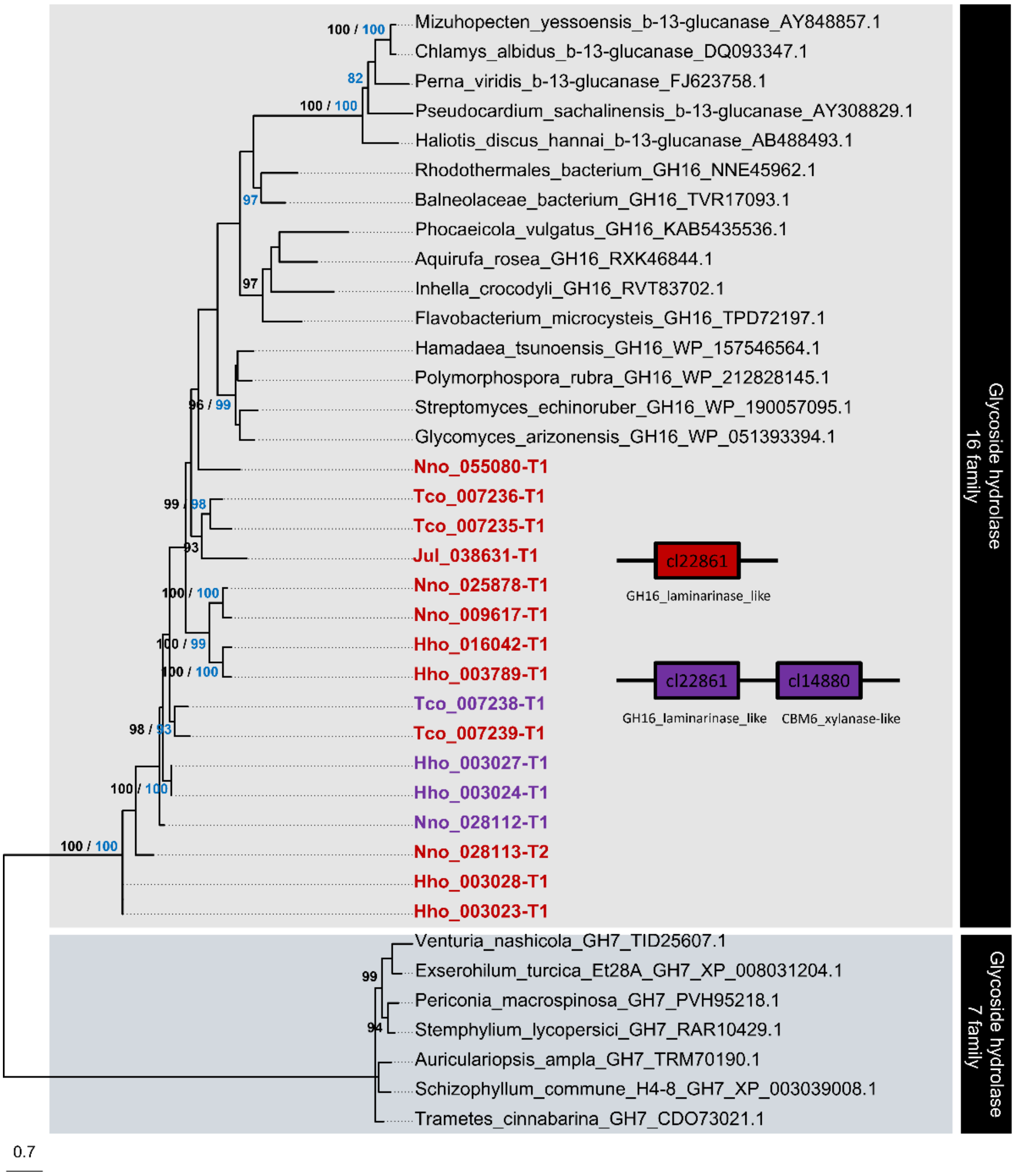

Supplementary Figure 12. Phylogenetic tree of glycoside hydrolase family 16 protein (GH16). Tree topology is displayed according to the Maximum Likelihood (ML) method (WAG+R3). Bootstrapping was conducted on each node with 1000 replicates on both ML (black) and NJ (blue) algorithms. Only bootstrap values larger than 80% are indicated for clarity. Hho, *H. holstii*; Nno, *N. nodulosa*; Tco, *T. corallinus*; Jul, *A. tonginus*.

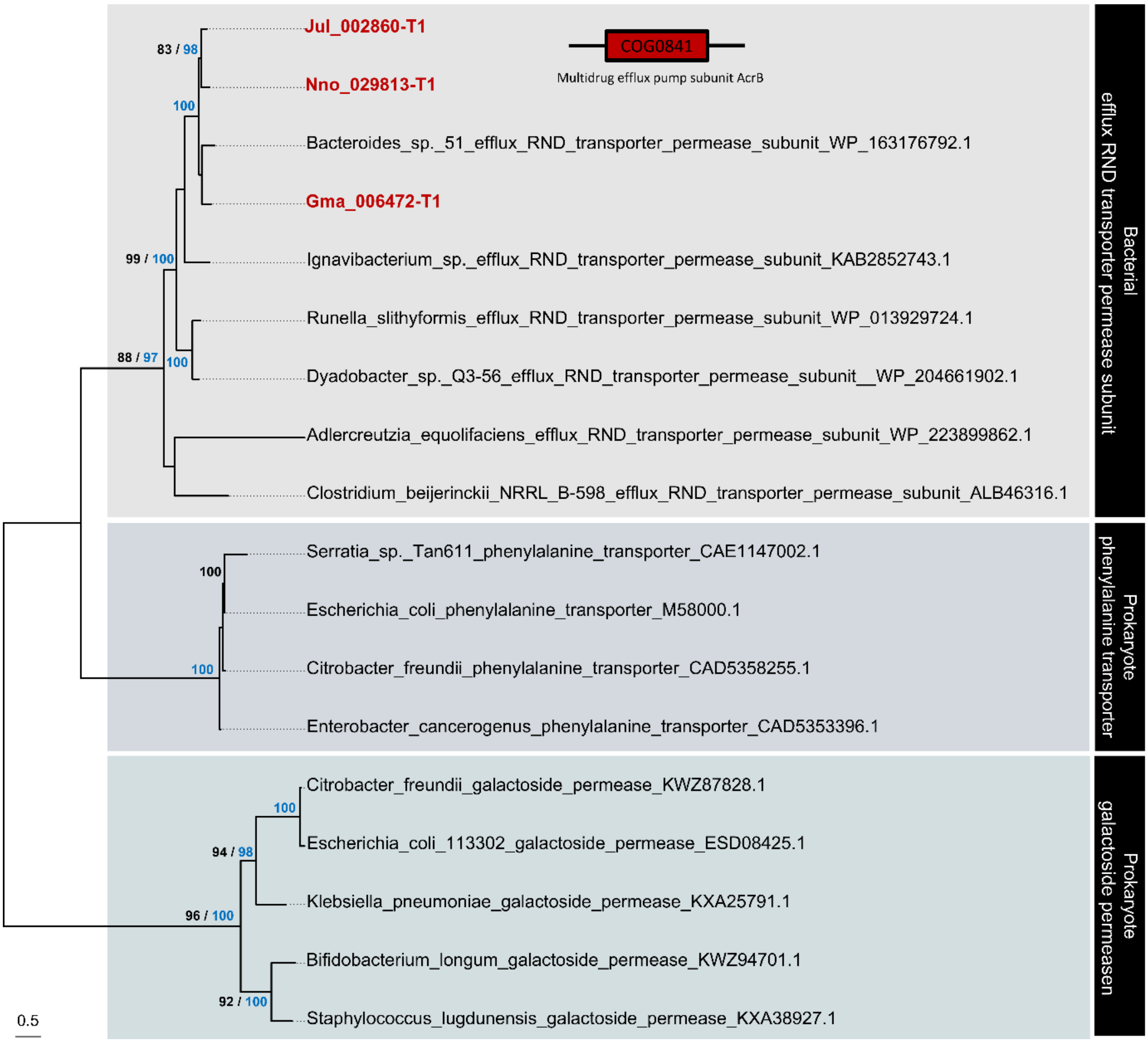

Supplementary Figure 13. Phylogenetic tree of efflux RND transporter permease subunit. Tree topology is displayed according to the Maximum Likelihood (ML) method (LG+F+R3). Bootstrapping was conducted on each node with 1000 replicates on both ML (black) and NJ (blue) algorithms. Only bootstrap values larger than 80% are indicated for clarity. Gma, *G. maerens*; Nno, *N. nodulosa*; Jul, *A. tonginus*.

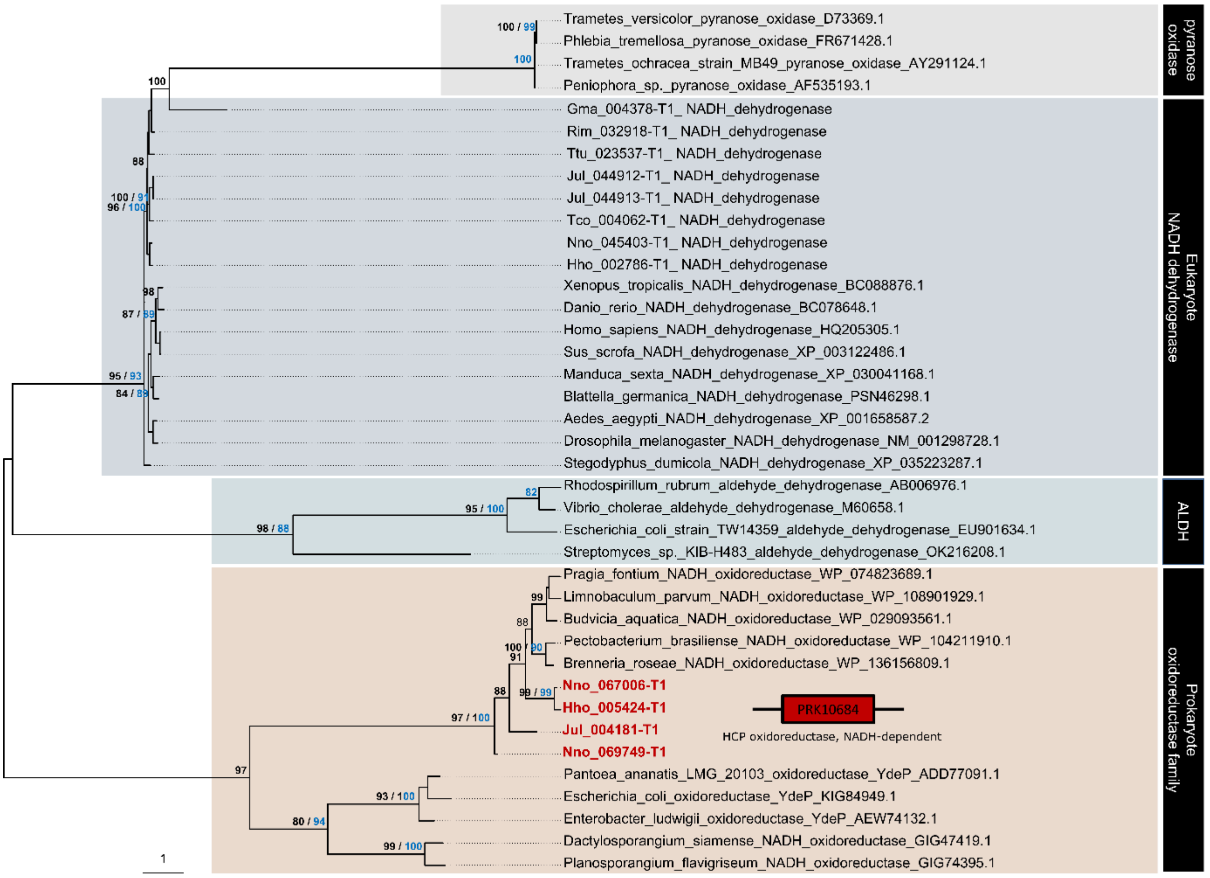

Supplementary Figure 14. Phylogenetic tree of NADH dehydrogenase. Tree topology is displayed according to the Maximum Likelihood (ML) method (WAG+R3). Bootstrapping was conducted on each node with 1000 replicates on both ML (black) and NJ (blue) algorithms. Only bootstrap values larger than 80% are indicated for clarity. Hho, *H. holstii*; Nno, *N. nodulosa*; Jul, *A. tonginus*.

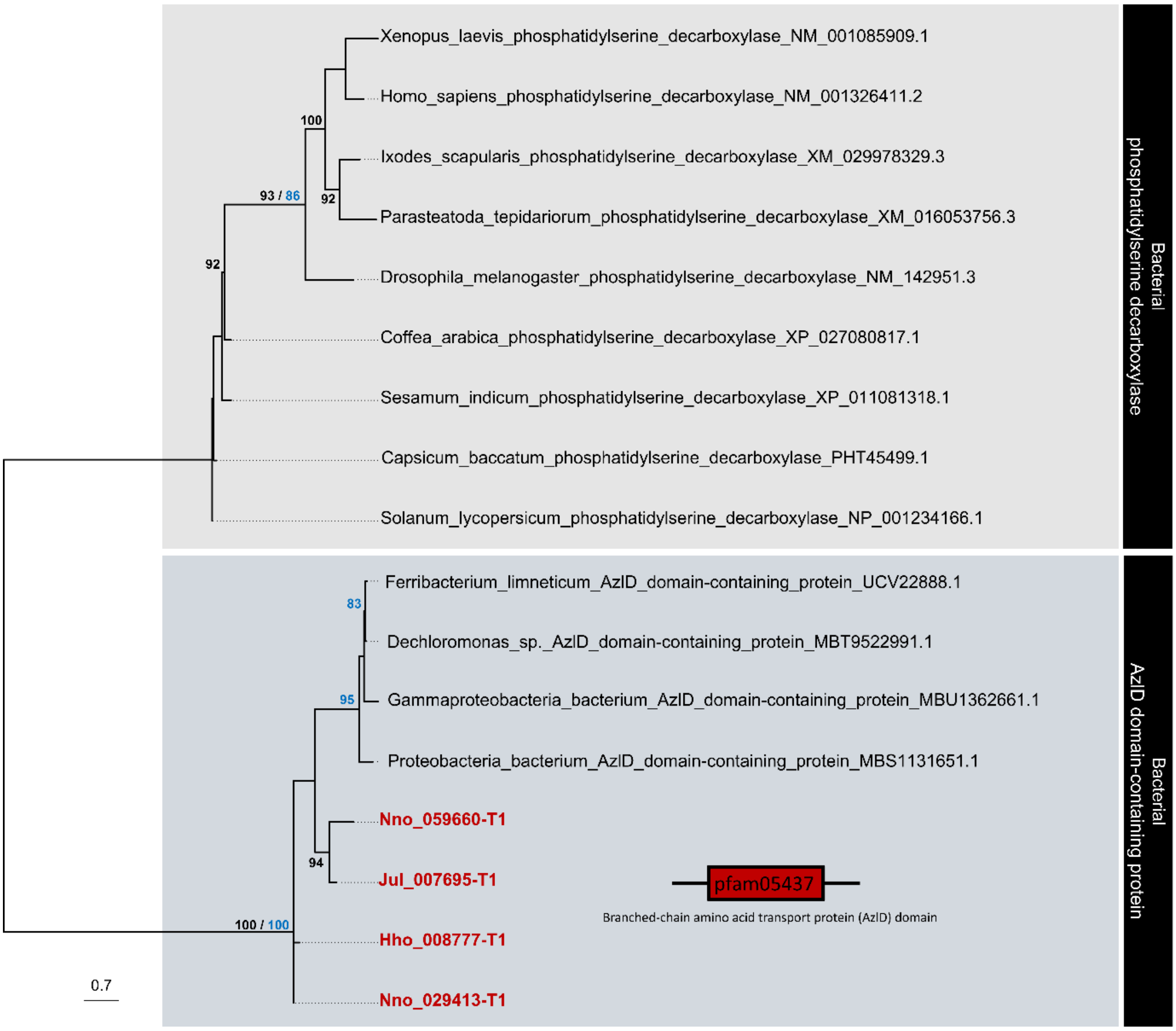

Supplementary Figure 15. Phylogenetic tree of AzlD domain-containing protein. Tree topology is displayed according to the Maximum Likelihood (ML) method (JTT+G4). Bootstrapping was conducted on each node with 1000 replicates on both ML (black) and NJ (blue) algorithms. Only bootstrap values larger than 80% are indicated for clarity. Hho, *H. holstii*; Nno, *N. nodulosa*; Jul, *A. tonginus*.

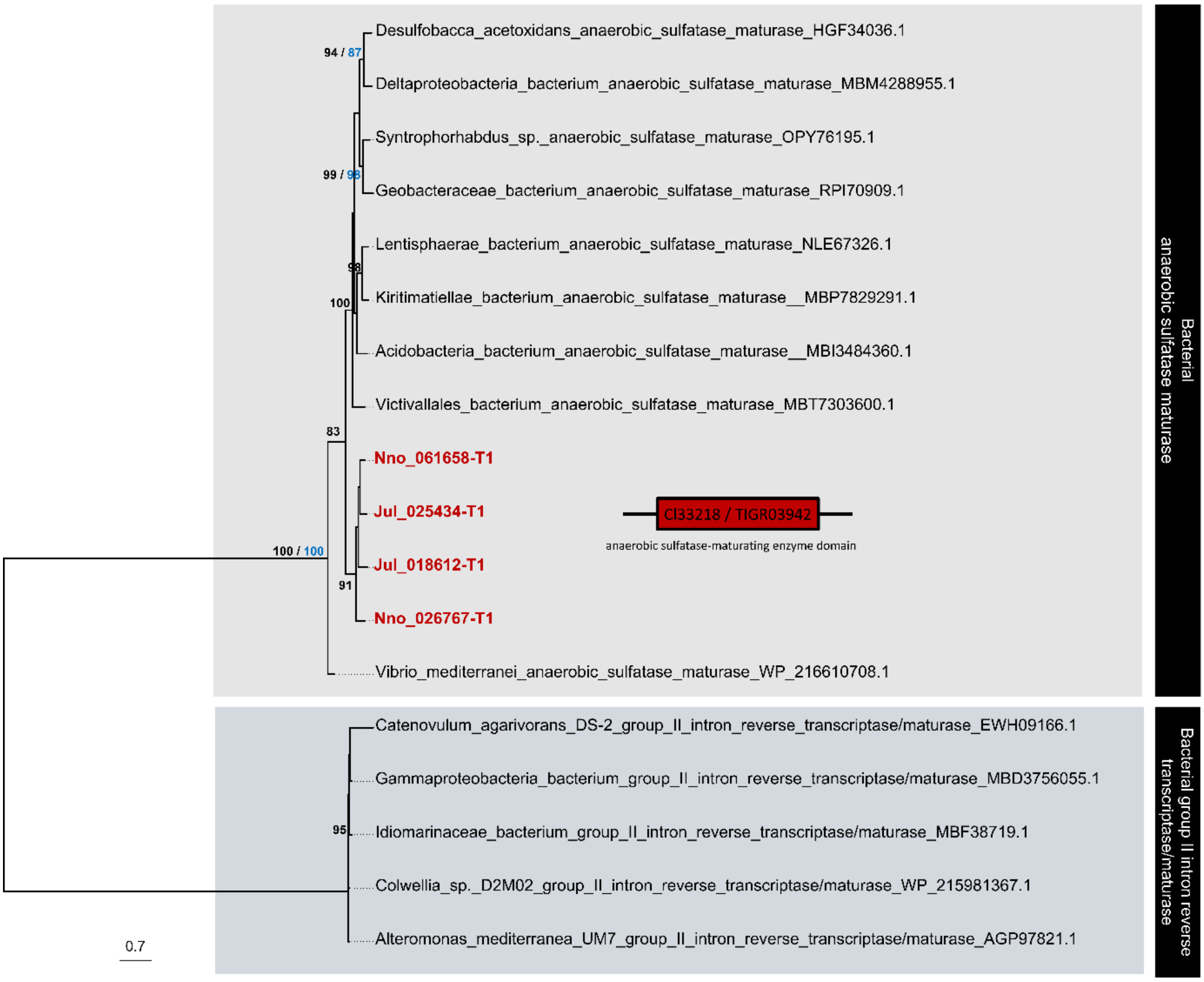

Supplementary Figure 16. Phylogenetic tree of anaerobic sulfatase maturase. Tree topology is displayed according to the Maximum Likelihood (ML) method (WAG+G4). Bootstrapping was conducted on each node with 1000 replicates on both ML (black) and NJ (blue) algorithms. Only bootstrap values larger than 80% are indicated for clarity. Nno, *N. nodulosa*; Jul, *A. tonginus*.

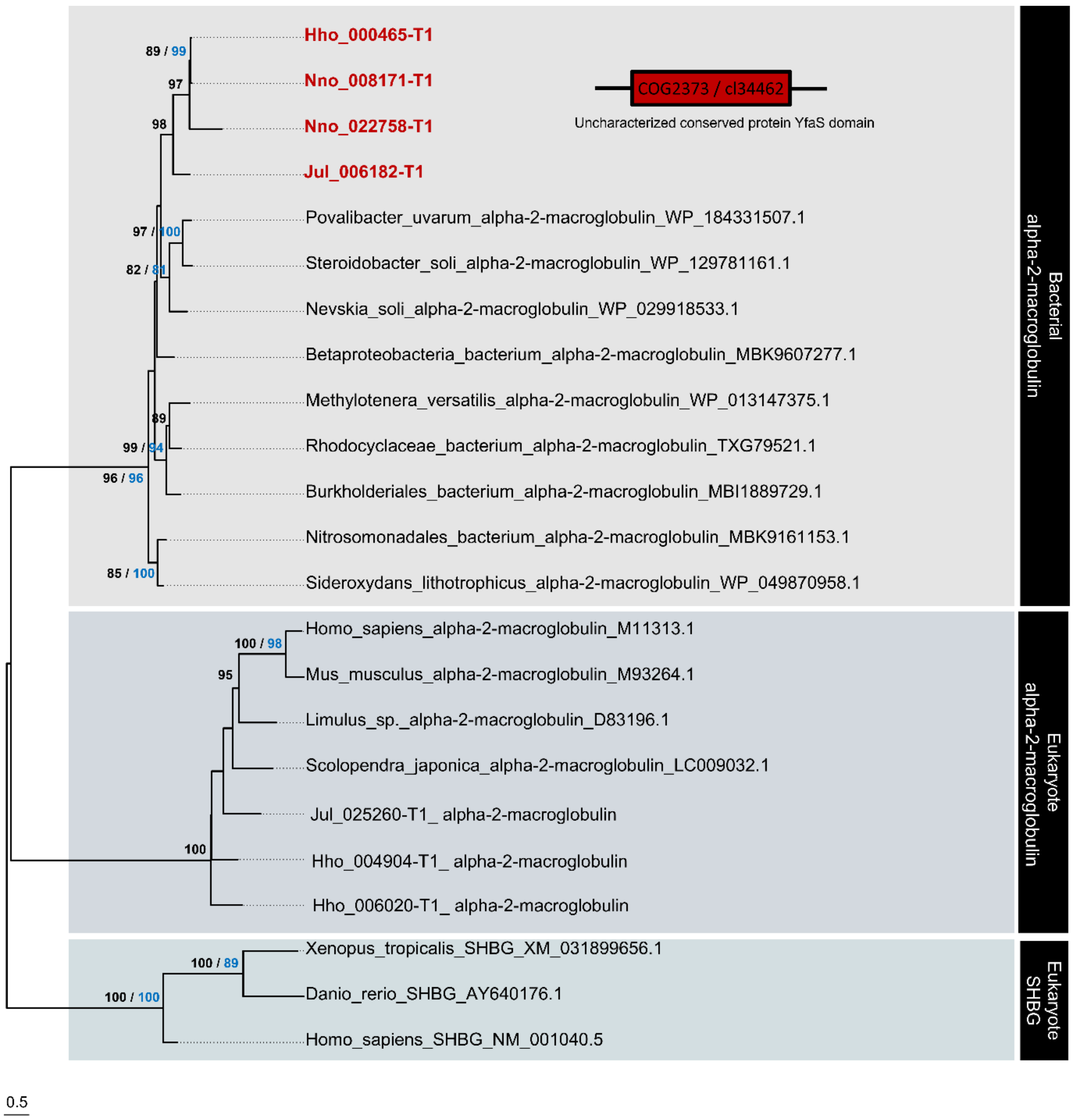

Supplementary Figure 17. Phylogenetic tree of alpha-2-macroglobulin. Tree topology is displayed according to the Maximum Likelihood (ML) method (WAG+F+G4). Bootstrapping was conducted on each node with 1000 replicates on both ML (black) and NJ (blue) algorithms. Only bootstrap values larger than 80% are indicated for clarity. Hho, *H. holstii*; Nno, *N. nodulosa*; Jul, *A. tonginus*.

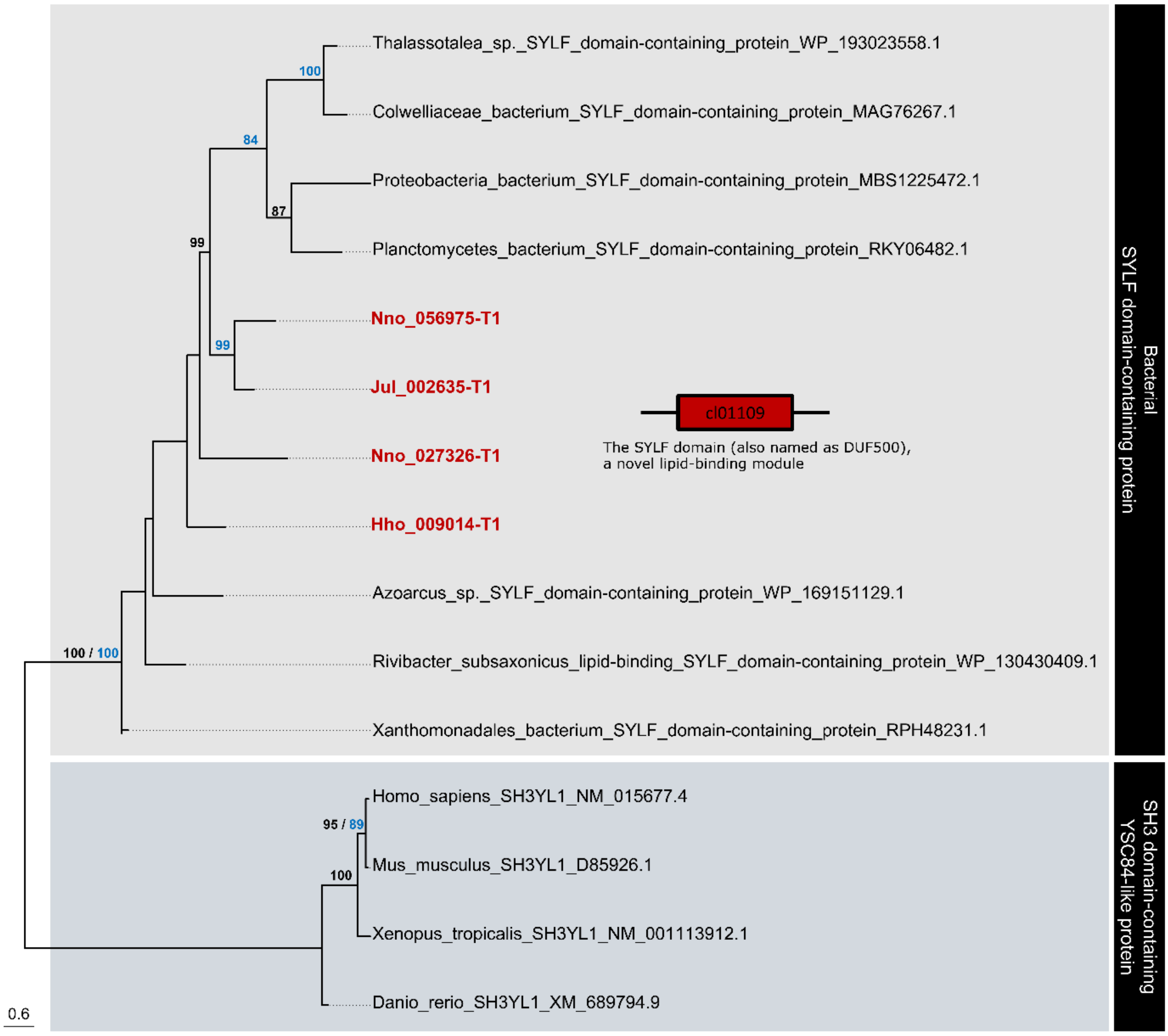

Supplementary Figure 18. Phylogenetic tree of SYLF domain-containing protein. Tree topology is displayed according to the Maximum Likelihood (ML) method (LG+R3). Bootstrapping was conducted on each node with 1000 replicates on both ML (black) and NJ (blue) algorithms. Only bootstrap values larger than 80% are indicated for clarity. Hho, *H. holstii*; Nno, *N. nodulosa*; Jul, *A. tonginus*.

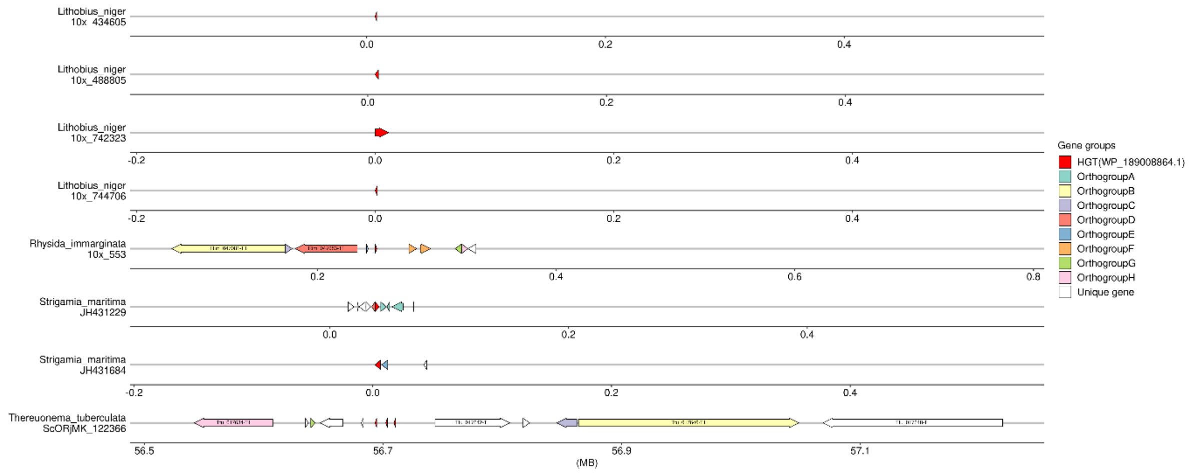

Supplementary Figure 19. Microsynteny of glucose/arabinose dehydrogenase (GDH; WP_189008864.1).

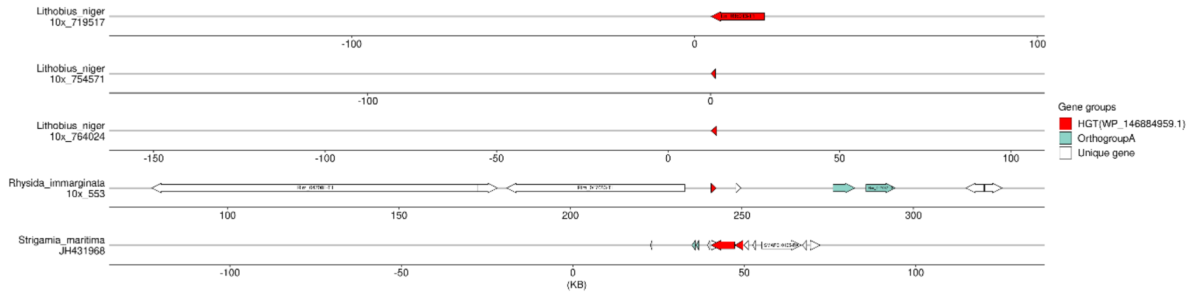

Supplementary Figure 20. Microsynteny of glucose/arabinose dehydrogenase (GDH; WP_146884959.1).

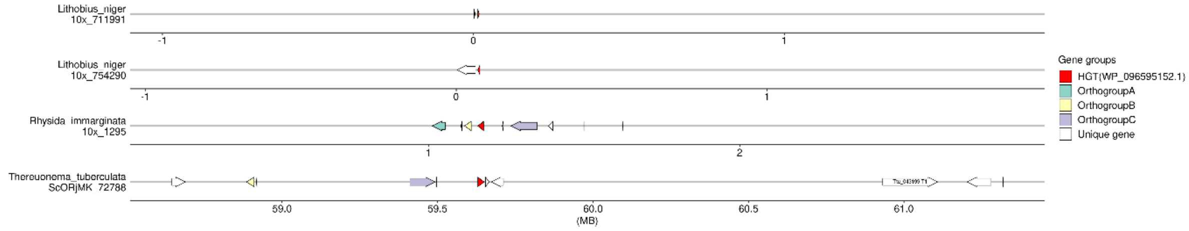

Supplementary Figure 21. Microsynteny of non-ribosomal peptide synthetase (NRPS; WP_096595152.1).

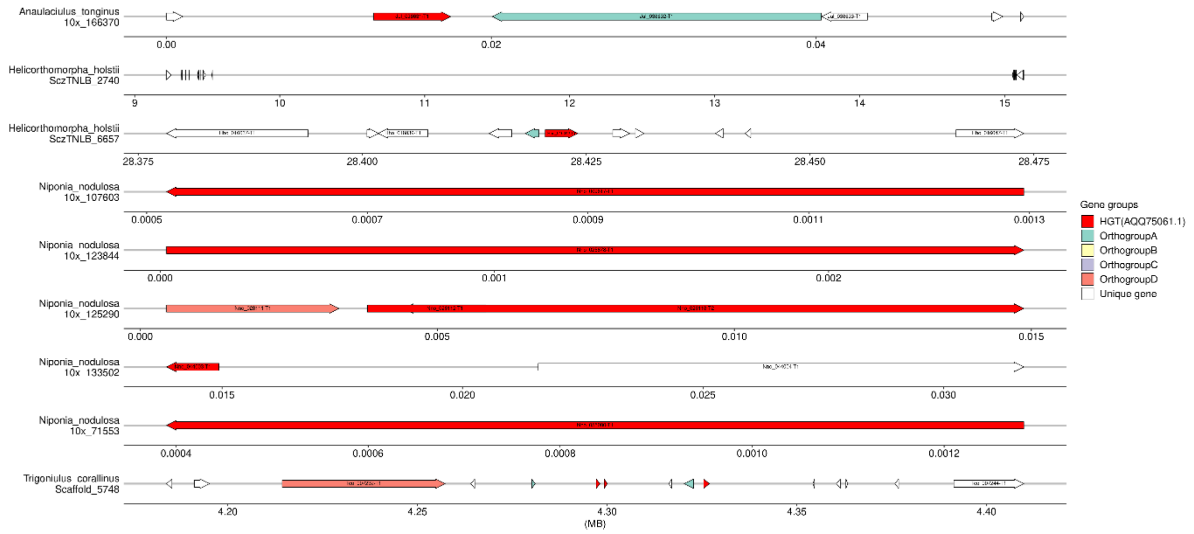

Supplementary Figure 22. Microsynteny of glycoside hydrolase family 16 protein (GH16; AQQ75061.1).

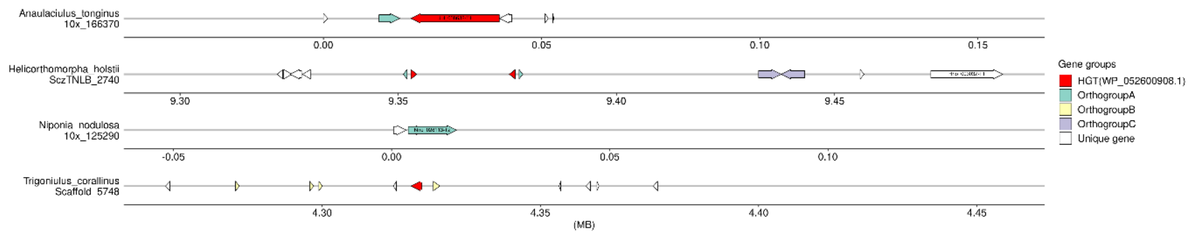

Supplementary Figure 23. Microsynteny of glycoside hydrolase family 16 protein (GH16; WP_052600908.1).

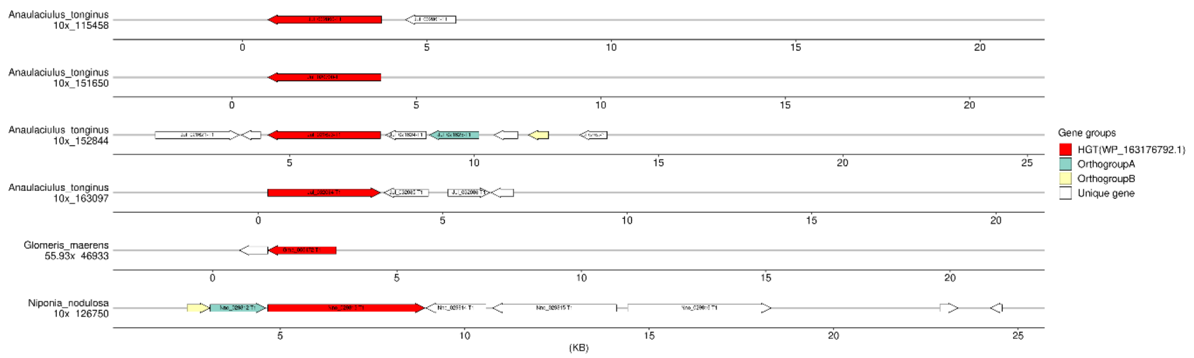

Supplementary Figure 24. Microsynteny of efflux RND transporter permease subunit (WP_163176792.1).

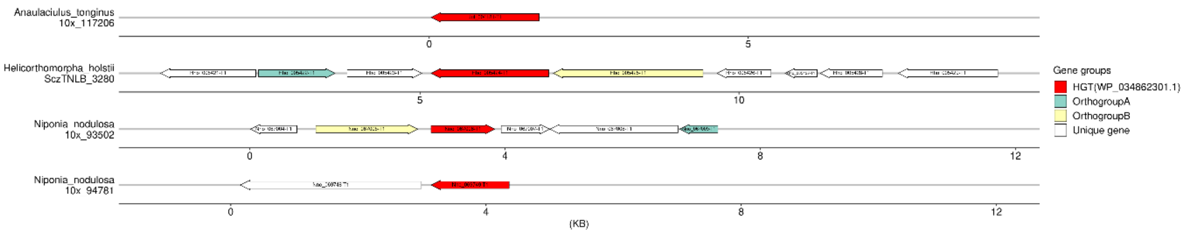

Supplementary Figure 25. Microsynteny of NADH dehydrogenase (WP_034862301.1).

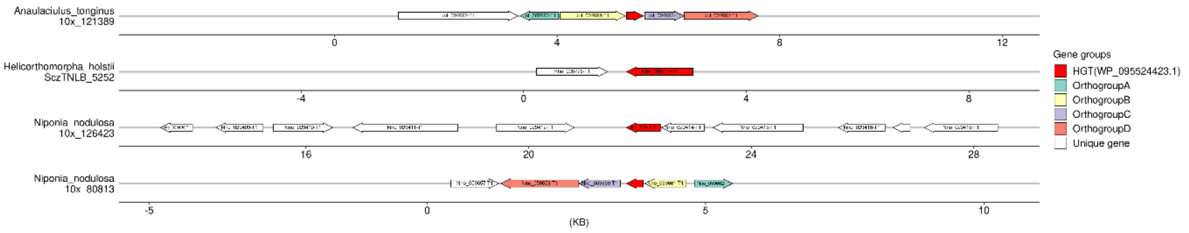

Supplementary Figure 26. Microsynteny of AzlD domain-containing protein (WP_095524423.1).

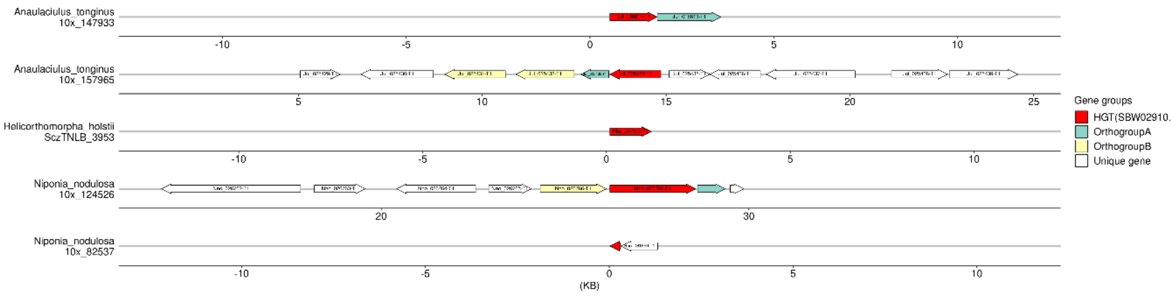

Supplementary Figure 27. Microsynteny of anaerobic sulfatase maturase (SBW02910.1).

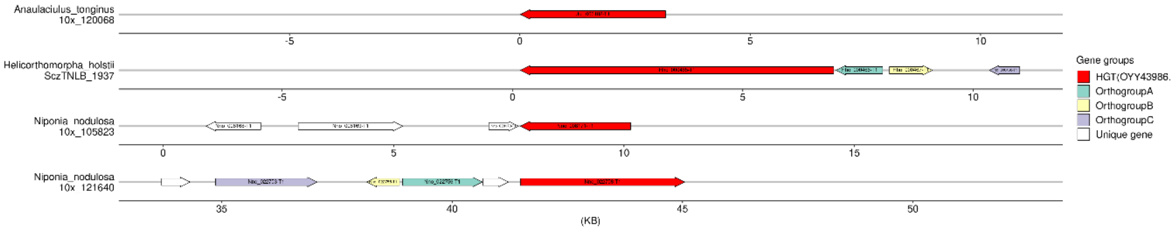

Supplementary Figure 28. Microsynteny of alpha-2-macroglobulin (OYY43986.1).

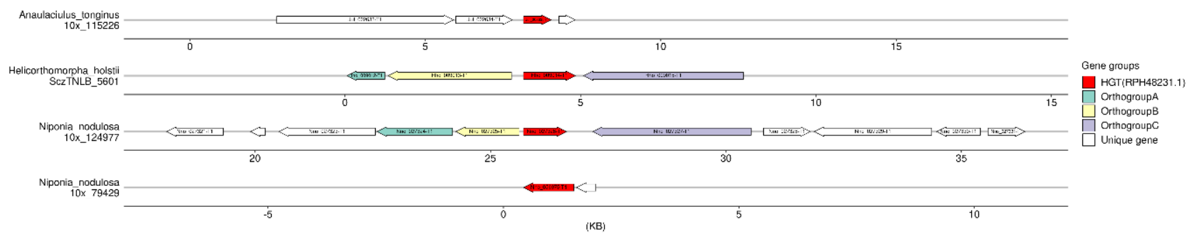

Supplementary Figure 29. Microsynteny of SYLF domain-containing protein (RPH48231.1).

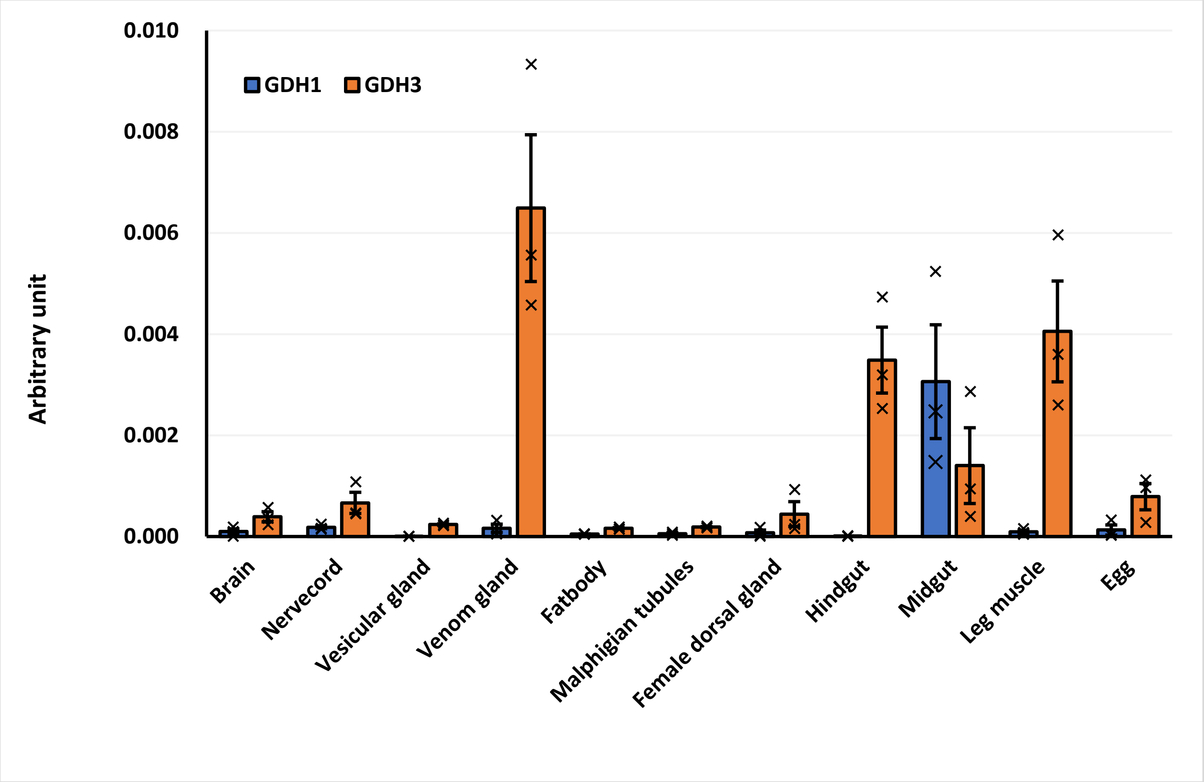

Supplementary Figure 30. Tissue differential expression of horizontal transferred WP_189008864.1 Glucose/arabinose dehydrogenases in adult female *T. tuberculata* (n=3 biologically independent *T. tuberculata*). Data are presented as mean values +/- SEM. Individual data points are present on each bar. The vertical error bars indicate the standard error of mean (SEM) among the data points in each experiment.

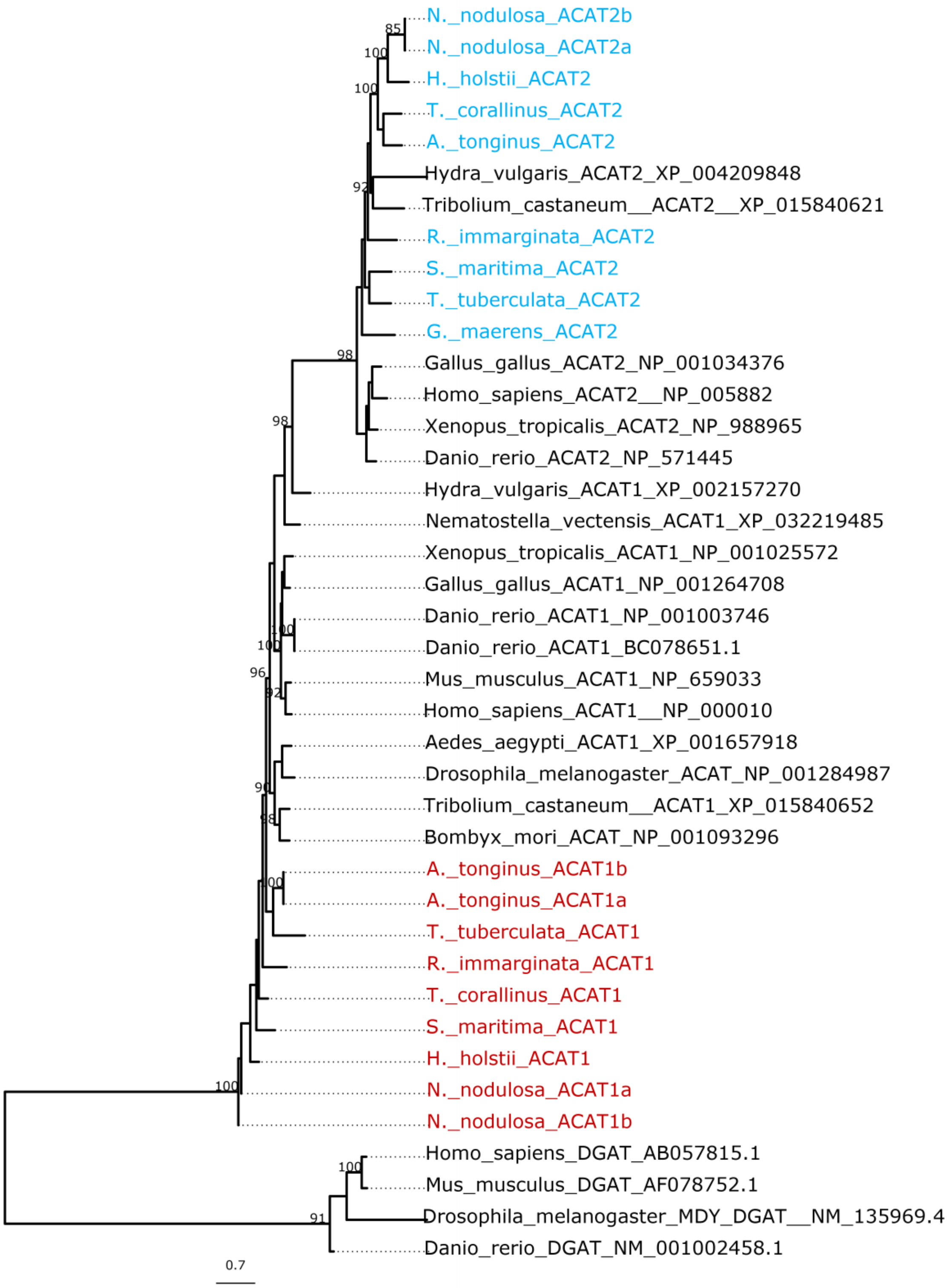

Supplementary Figure 31. Phylogenetic tree of acetyl-CoA C-acetyltransferase (ACAT). Tree is constructed with the maximum likelihood (ML) method with 1000 bootstrap replicates. Only bootstrap values larger than 80% are indicated for clarity. Tree rooted using metazoan diglyceride acyltransferase (DGAT).

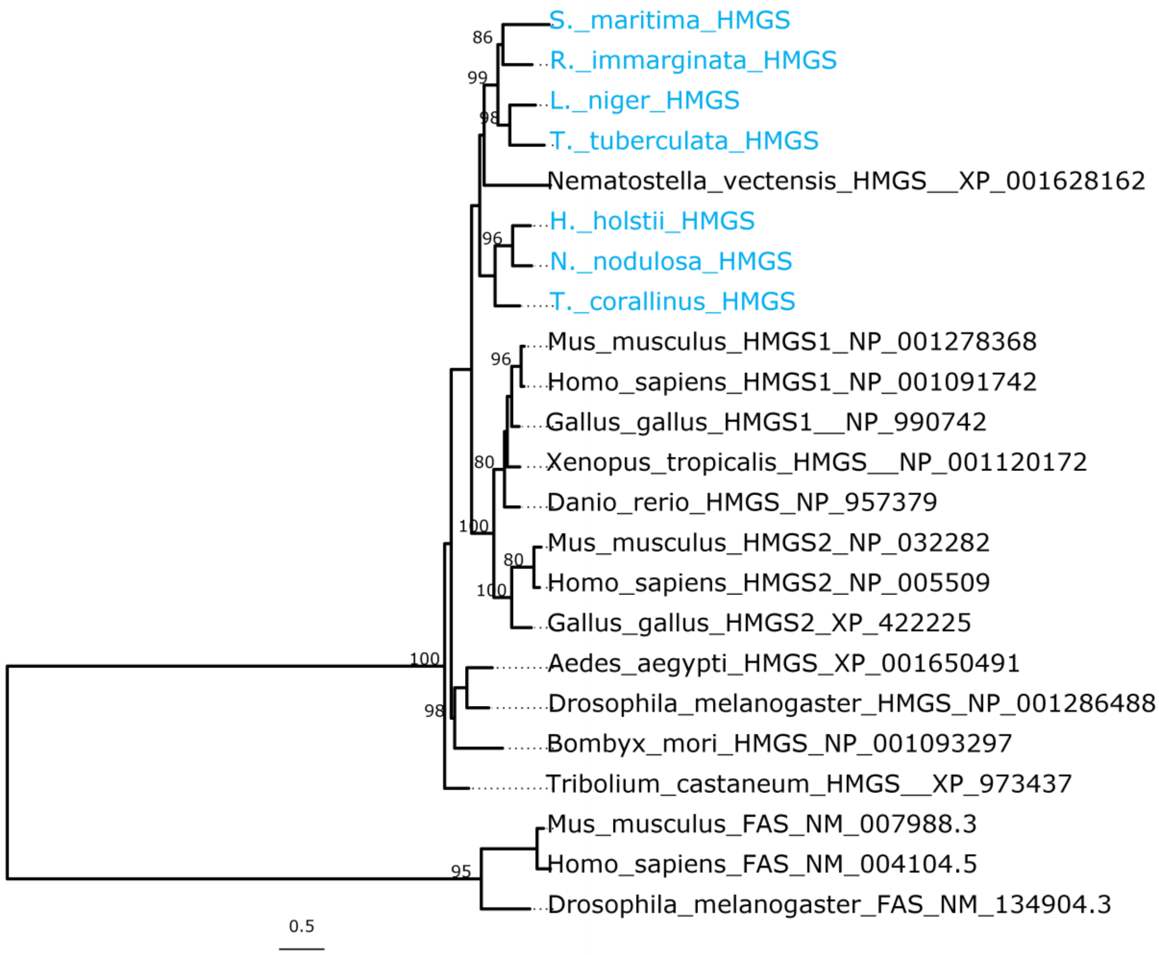

Supplementary Figure 32. Phylogenetic tree of hydroxymethylglutaryl-CoA synthase (HMGS). Tree is constructed with the maximum likelihood (ML) method with 1000 bootstrap replicates. Only bootstrap values larger than 80% are indicated for clarity. Tree rooted using metazoan fatty acid synthase (FAS).

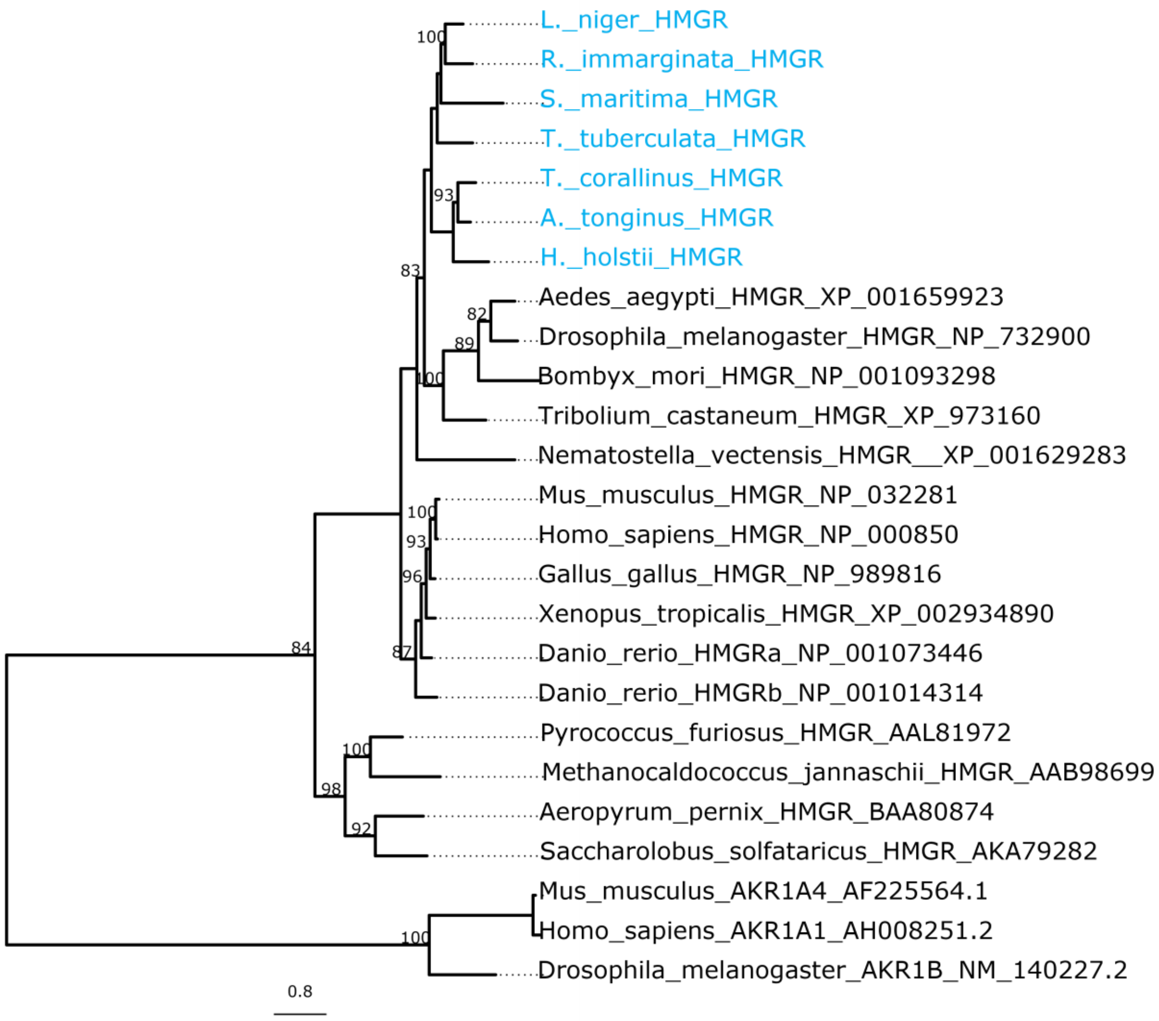

Supplementary Figure 33. Phylogenetic tree of hydroxymethylglutaryl-CoA reductase (HMGR). Tree is constructed with the maximum likelihood (ML) method with 1000 bootstrap replicates. Only bootstrap values larger than 80% are indicated for clarity. Tree rooted using metazoan aldo-keto reductase (AKR1).

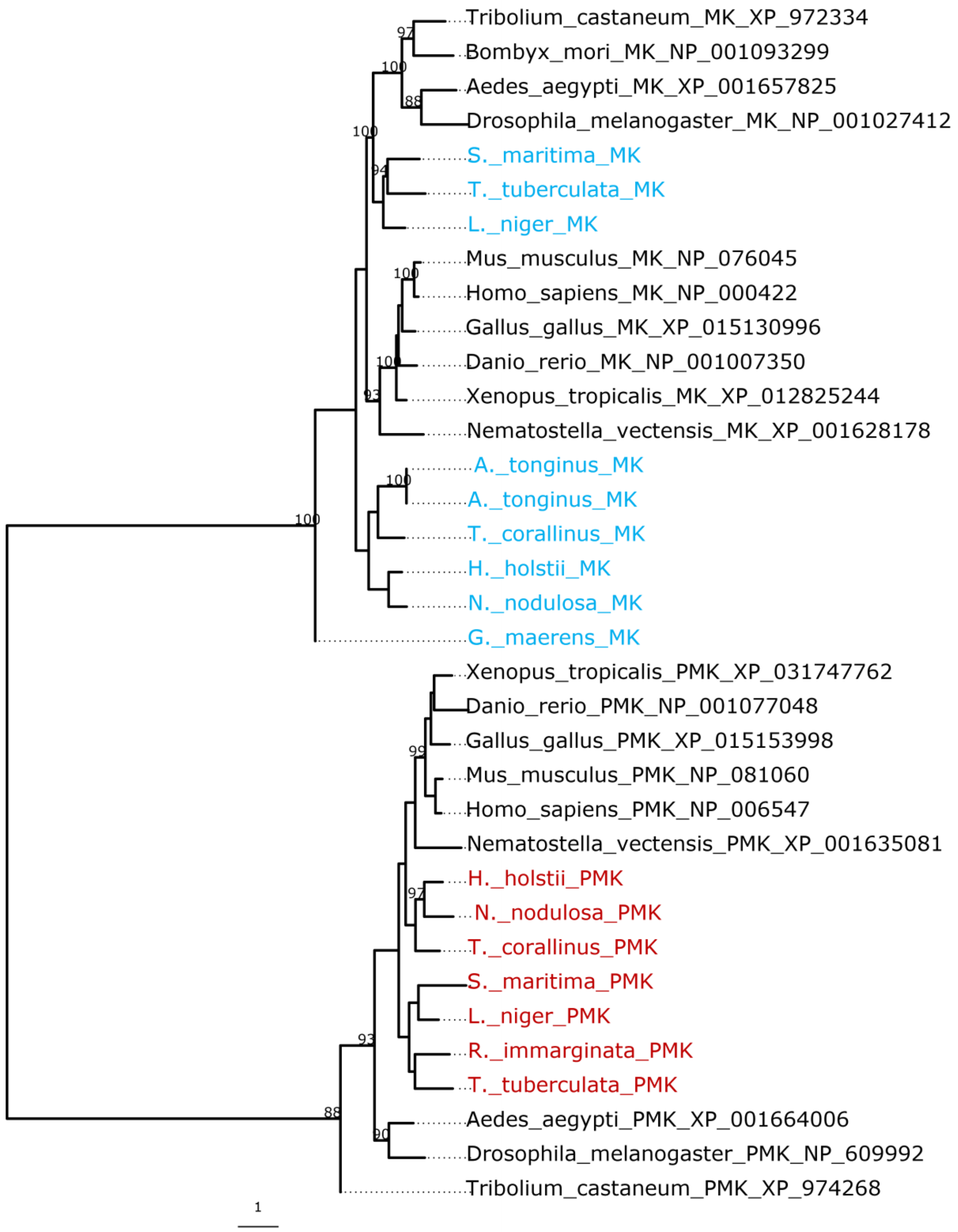

Supplementary Figure 34. Phylogenetic tree of mevalonate kinase (MK) and phosphomevalonate kinase (PMK). Tree is constructed with the maximum likelihood (ML) method with 1000 bootstrap replicates. Only bootstrap values larger than 80% are indicated for clarity.

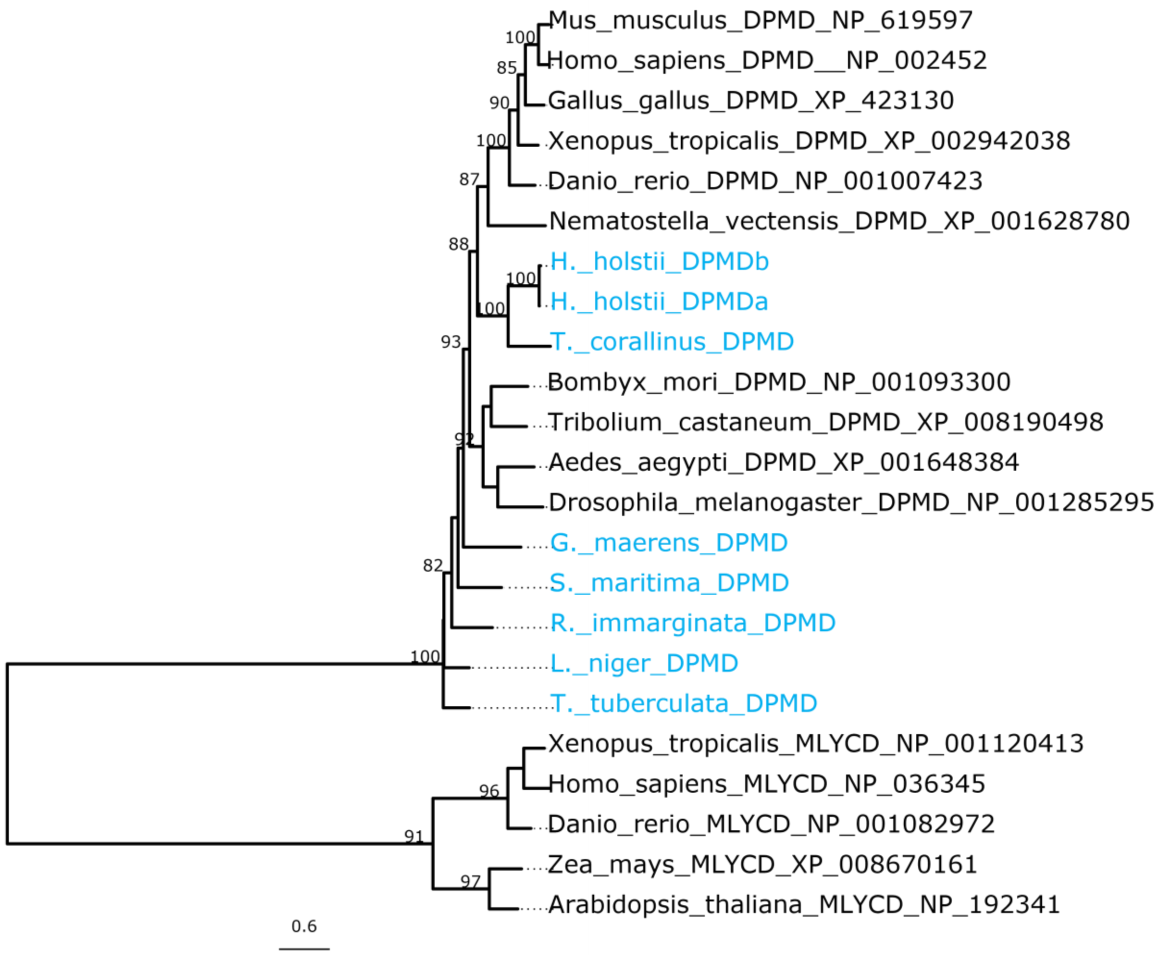

Supplementary Figure 35. Phylogenetic tree of diphosphomevalonate decarboxylase (DPMD). Tree is constructed with the maximum likelihood (ML) method with 1000 bootstrap replicates. Only bootstrap values larger than 80% are indicated for clarity. Tree rooted using malonyl-CoA decarboxylase (MLYCD).

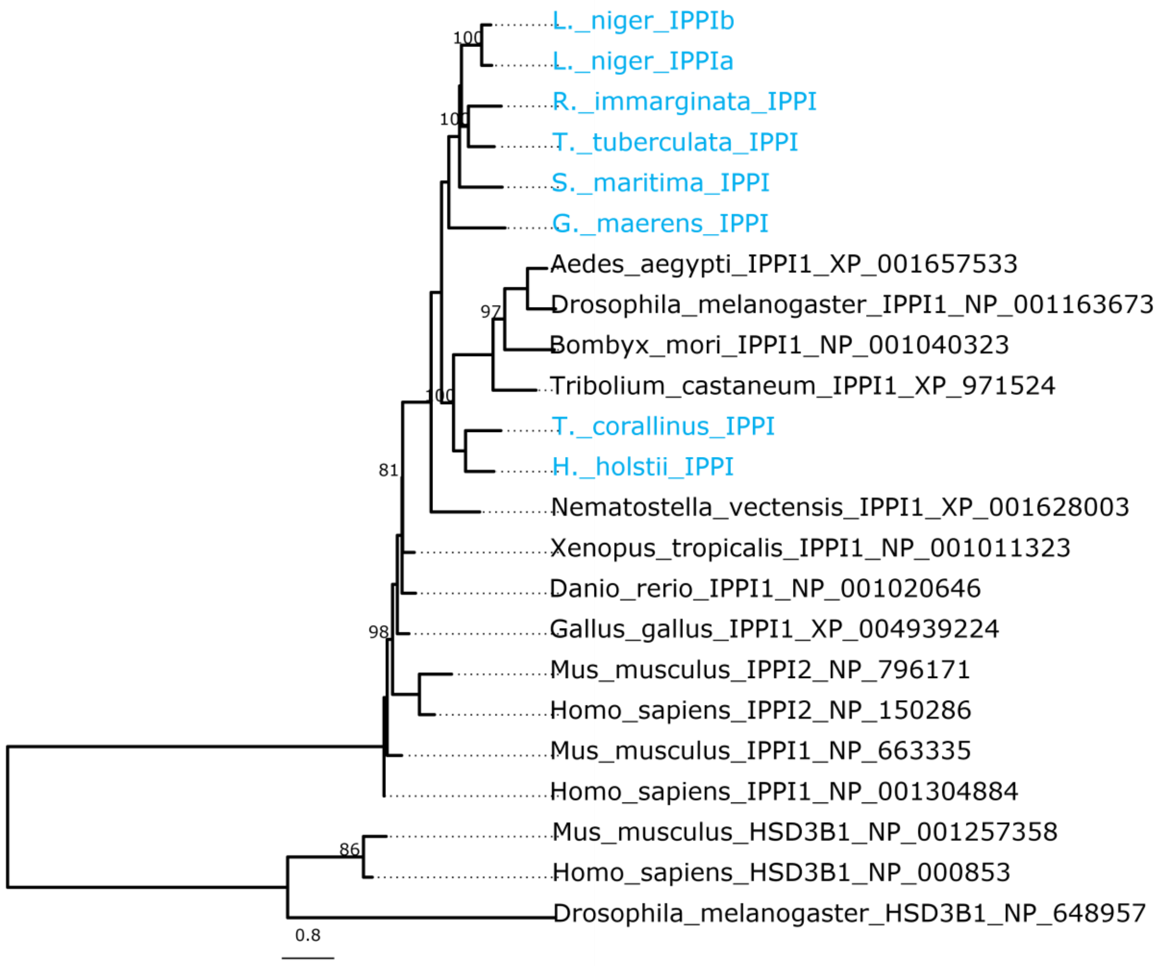

Supplementary Figure 36. Phylogenetic tree of isopentenyl-diphosphate delta-isomerase (IPPI). Tree is constructed with the maximum likelihood (ML) method with 1000 bootstrap replicates. Only bootstrap values larger than 80% are indicated for clarity. Tree rooted using metazoan hydroxy-delta-5-steroid dehydrogenase, 3 beta- and steroid delta-isomerase 1 (HSD3B1).

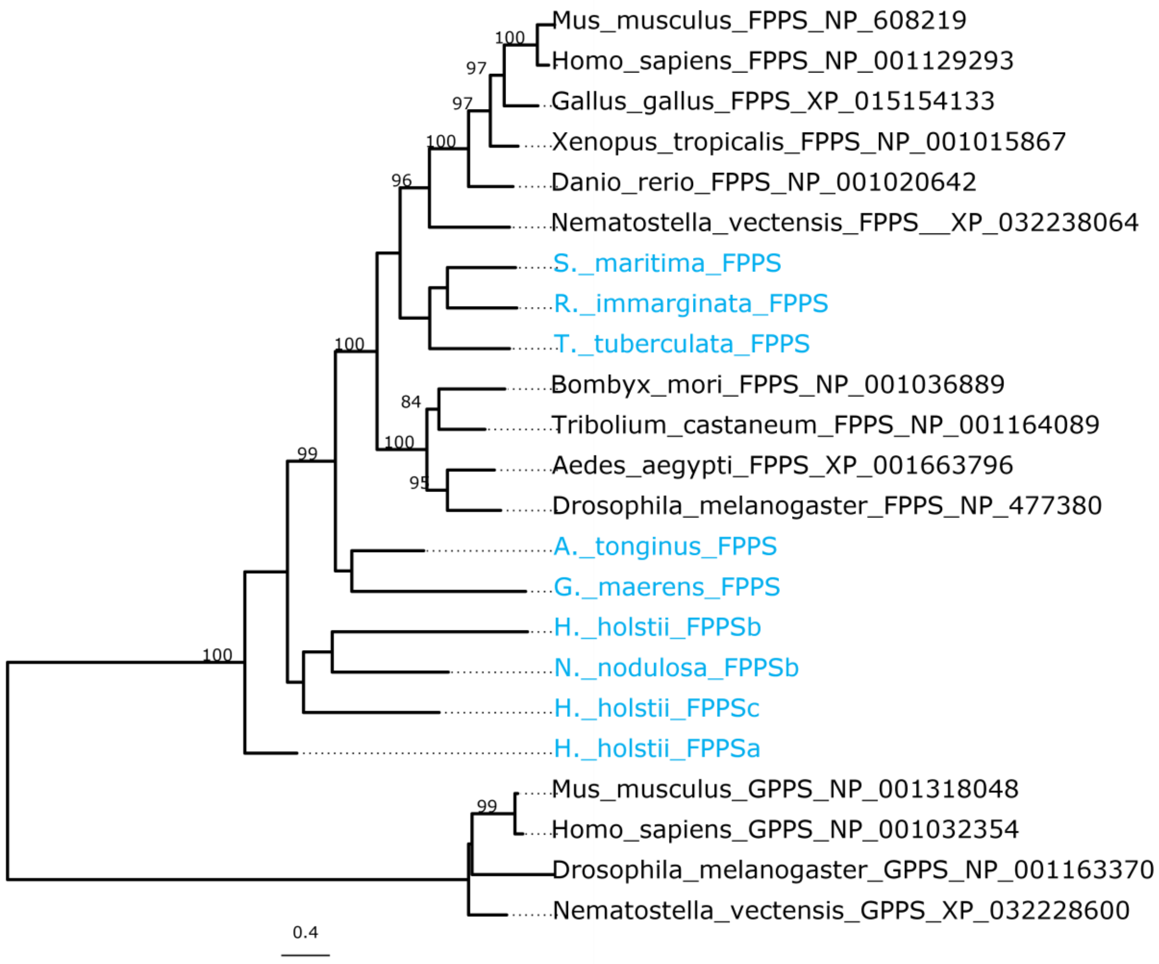

Supplementary Figure 37. Phylogenetic tree of farnesyl pyrophosphate synthase (FPPS). Tree is constructed with the maximum likelihood (ML) method with 1000 bootstrap replicates. Only bootstrap values larger than 80% are indicated for clarity. Tree rooted using metazoan geranyl diphosphate synthase (GPPS).

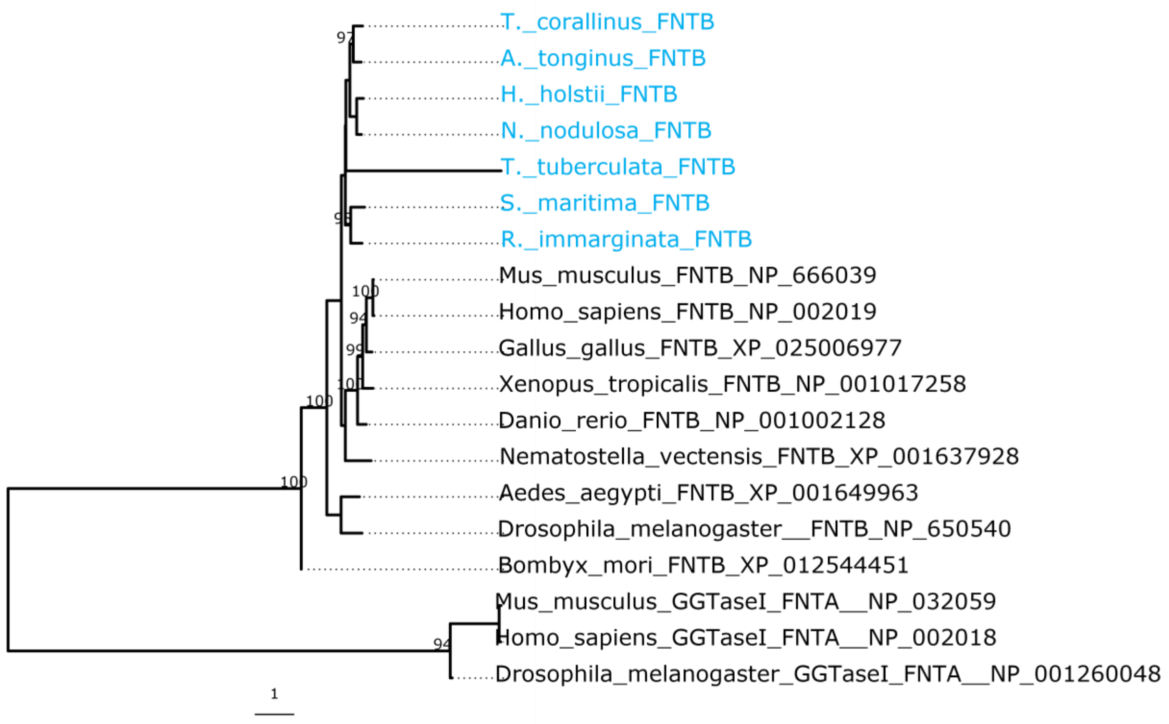

Supplementary Figure 38. Phylogenetic tree of farnesyltransferase beta (FNTB). Tree is constructed with the maximum likelihood (ML) method with 1000 bootstrap replicates. Only bootstrap values larger than 80% are indicated for clarity. Tree rooted using metazoan geranylgeranyltransferase (FNTA).

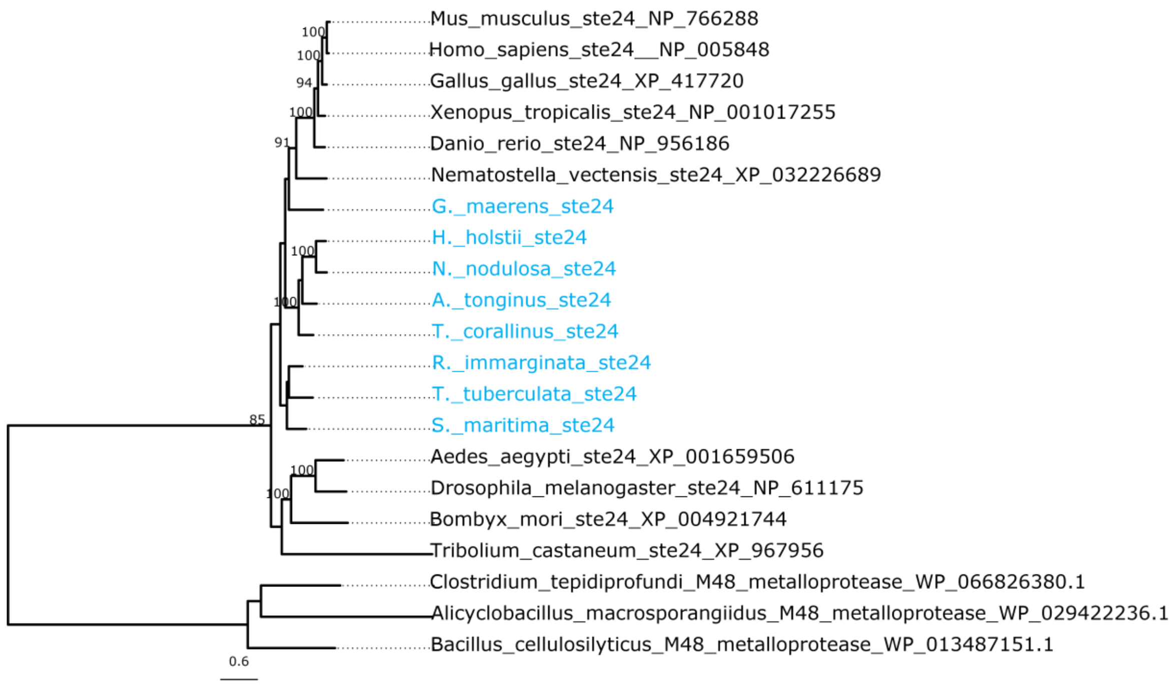

Supplementary Figure 39. Phylogenetic tree of ste24 endopeptidase (ste24). Tree is constructed with the maximum likelihood (ML) method with 1000 bootstrap replicates. Only bootstrap values larger than 80% are indicated for clarity. Tree rooted using protozoan M48 metalloprotease.

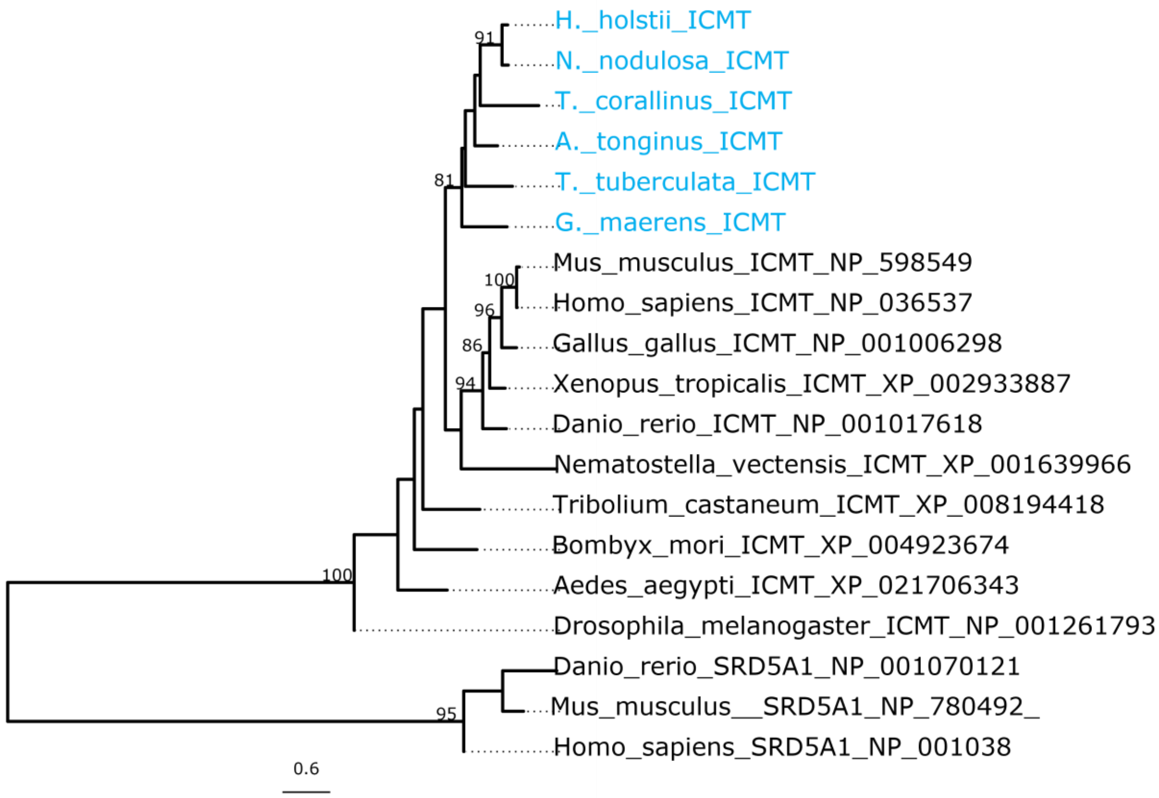

Supplementary Figure 40. Phylogenetic tree of protein-S-isoprenylcysteine O-methyltransferase (ICMT). Tree is constructed with the maximum likelihood (ML) method with 1000 bootstrap replicates. Only bootstrap values larger than 80% are indicated for clarity. Tree rooted using metazoan steroid 5 alpha-reductase 1 (SRD5A1).

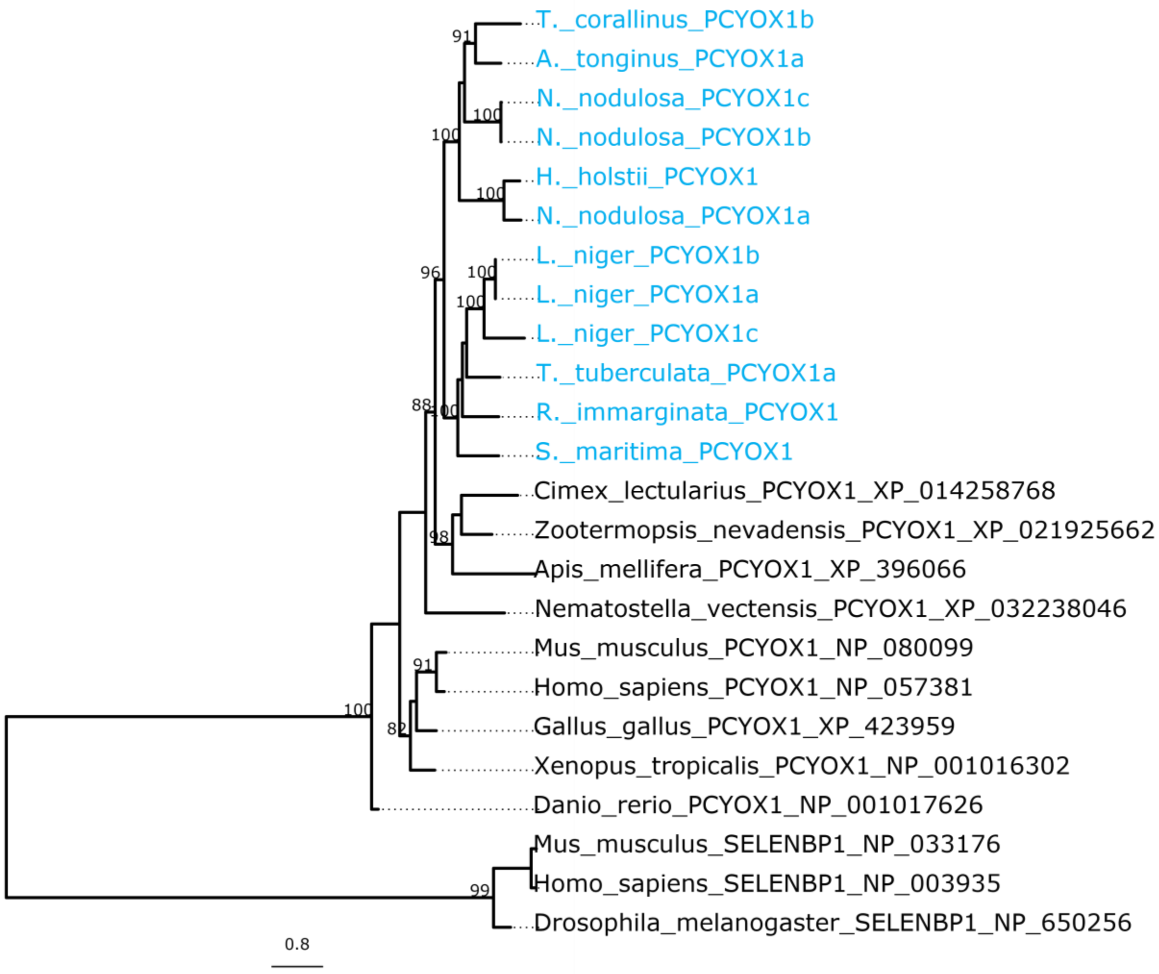

Supplementary Figure 41. Phylogenetic tree of prenylcysteine oxidase (PCYOX1). Tree is constructed with the maximum likelihood (ML) method with 1000 bootstrap replicates. Only bootstrap values larger than 80% are indicated for clarity. Tree rooted using metazoan selenium binding protein 1 (SELENBP1).

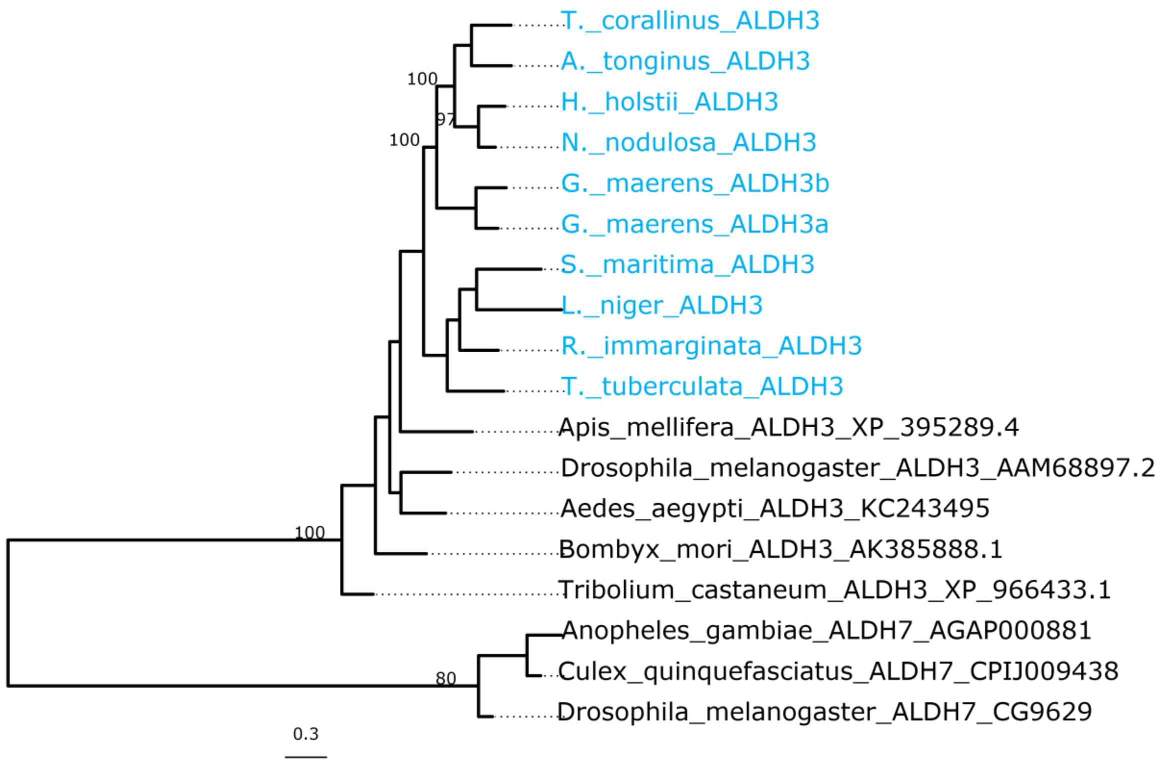

Supplementary Figure 42. Phylogenetic tree of aldehyde dehydrogenase 3 (ALDH3). Tree is constructed with the maximum likelihood (ML) method with 1000 bootstrap replicates. Only bootstrap values larger than 80% are indicated for clarity. Tree rooted using arthropod aldehyde dehydrogenase 7 (ALDH7).

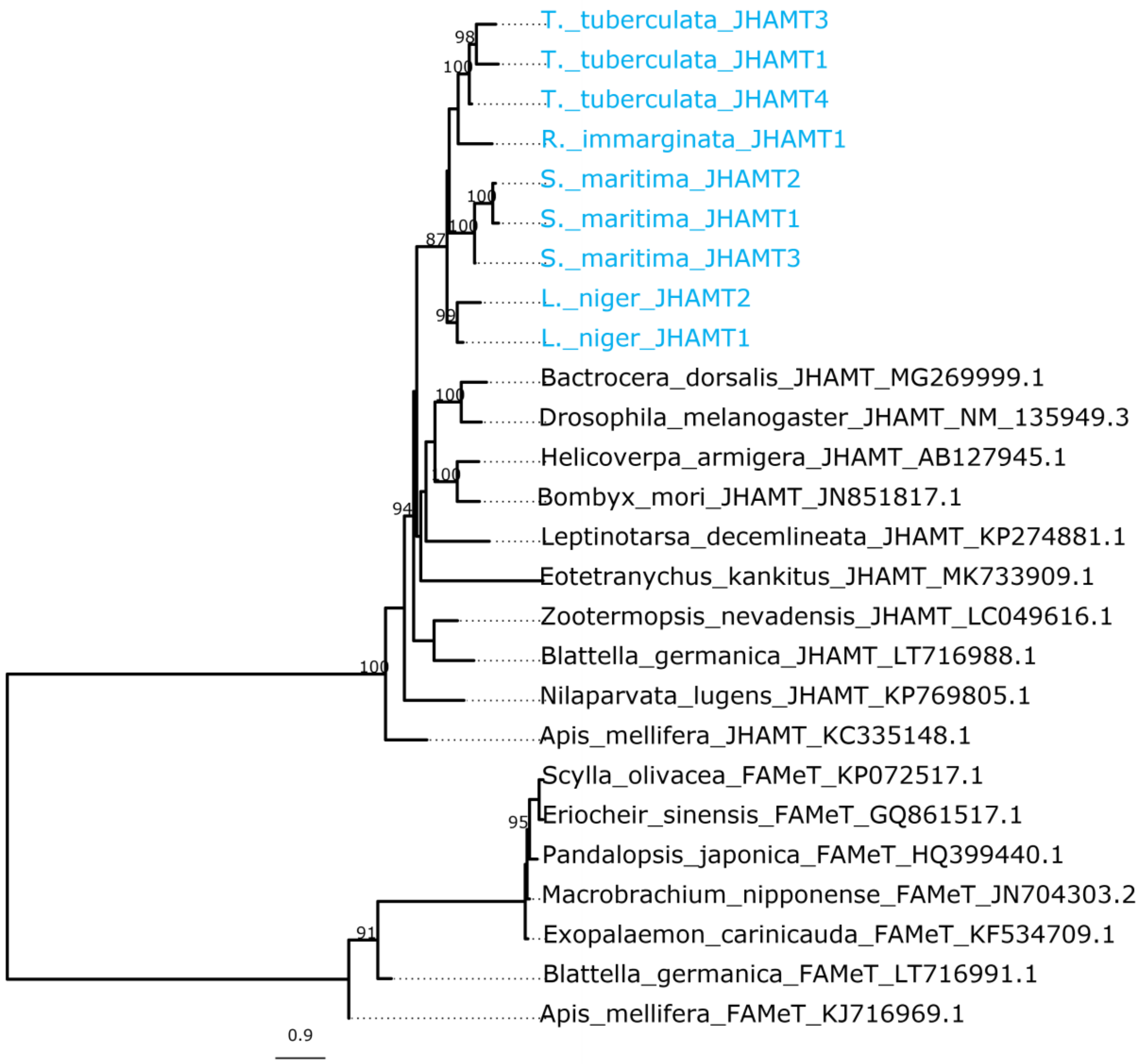

Supplementary Figure 43. Phylogenetic tree of juvenile hormone acid methyltransferase (JHAMT). Tree is constructed with the maximum likelihood (ML) method with 1000 bootstrap replicates. Only bootstrap values larger than 80% are indicated for clarity. Tree rooted using arthropod farnesoic acid methyltransferase (FAMeT).

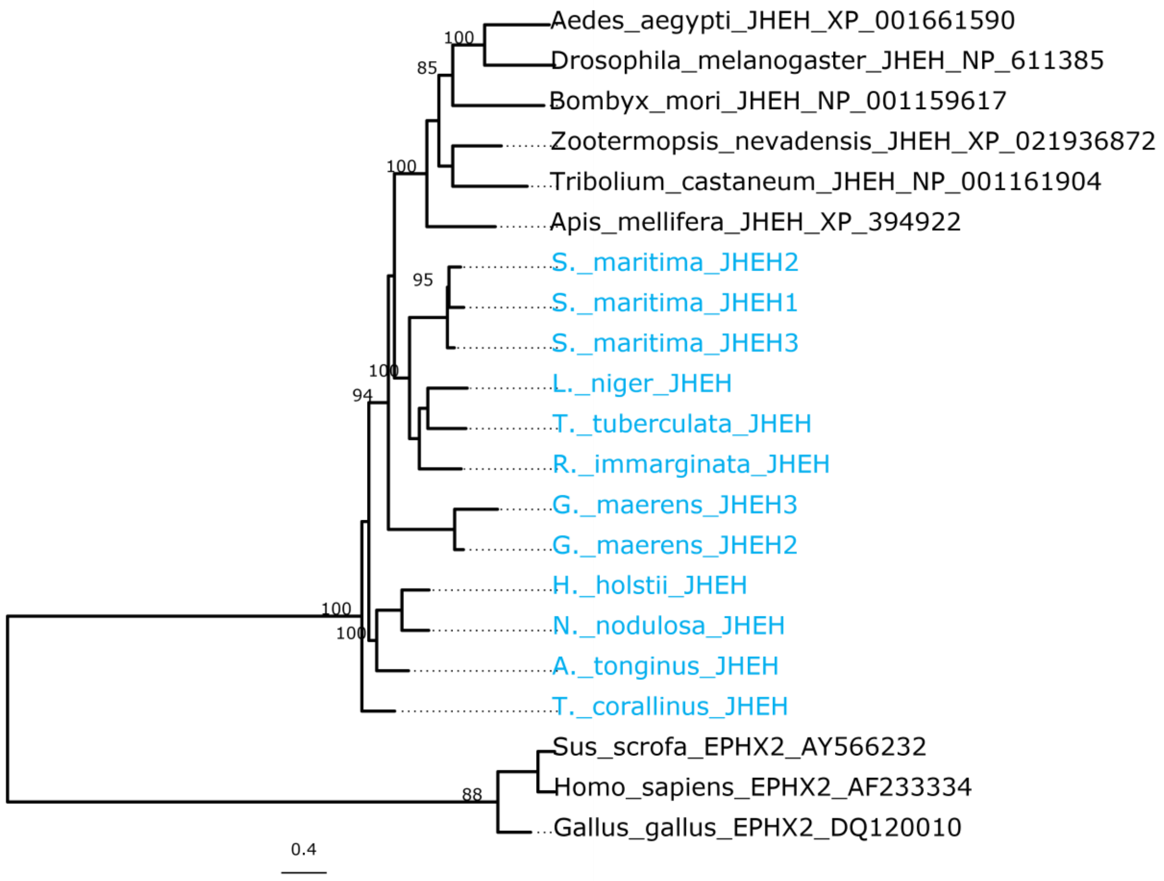

Supplementary Figure 44. Phylogenetic tree of juvenile hormone epoxide hydrolase (JHEH). Tree is constructed with the maximum likelihood (ML) method with 1000 bootstrap replicates. Only bootstrap values larger than 80% are indicated for clarity. Tree rooted using mammalian epoxide hydrolase (EPHX).

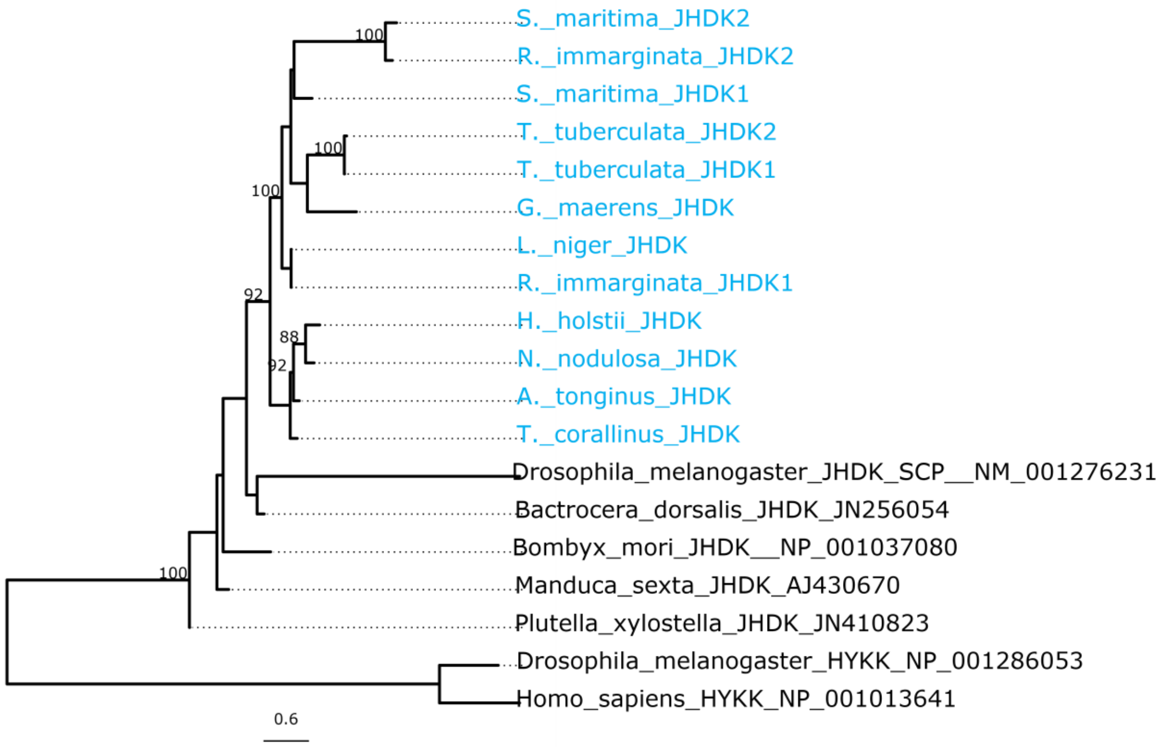

Supplementary Figure 45. Phylogenetic tree of juvenile hormone diol kinase (JHDK). Tree is constructed with the maximum likelihood (ML) method with 1000 bootstrap replicates. Only bootstrap values larger than 80% are indicated for clarity. Tree rooted using metazoan hydroxylysine kinase (HYKK).

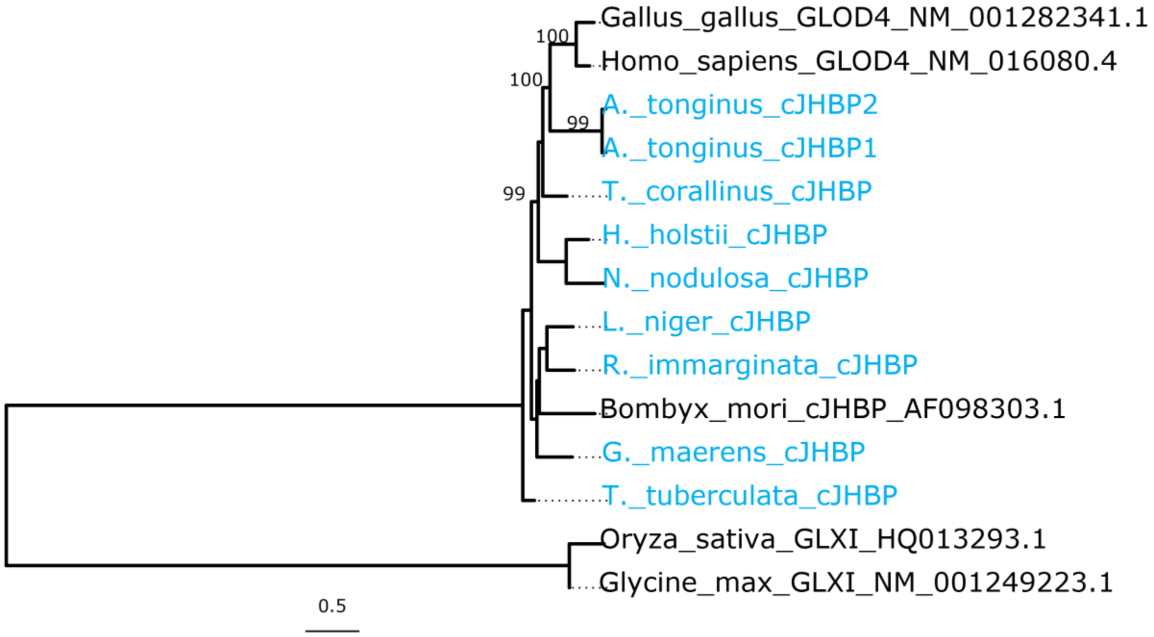

Supplementary Figure 46. Phylogenetic tree of cytosolic juvenile hormone binding protein (cJHBP)/glyoxalase domain containing protein 4 (GLOD4). Tree is constructed with the maximum likelihood (ML) method with 1000 bootstrap replicates. Only bootstrap values larger than 80% are indicated for clarity. Tree rooted using plant glyoxalase (GLXI).

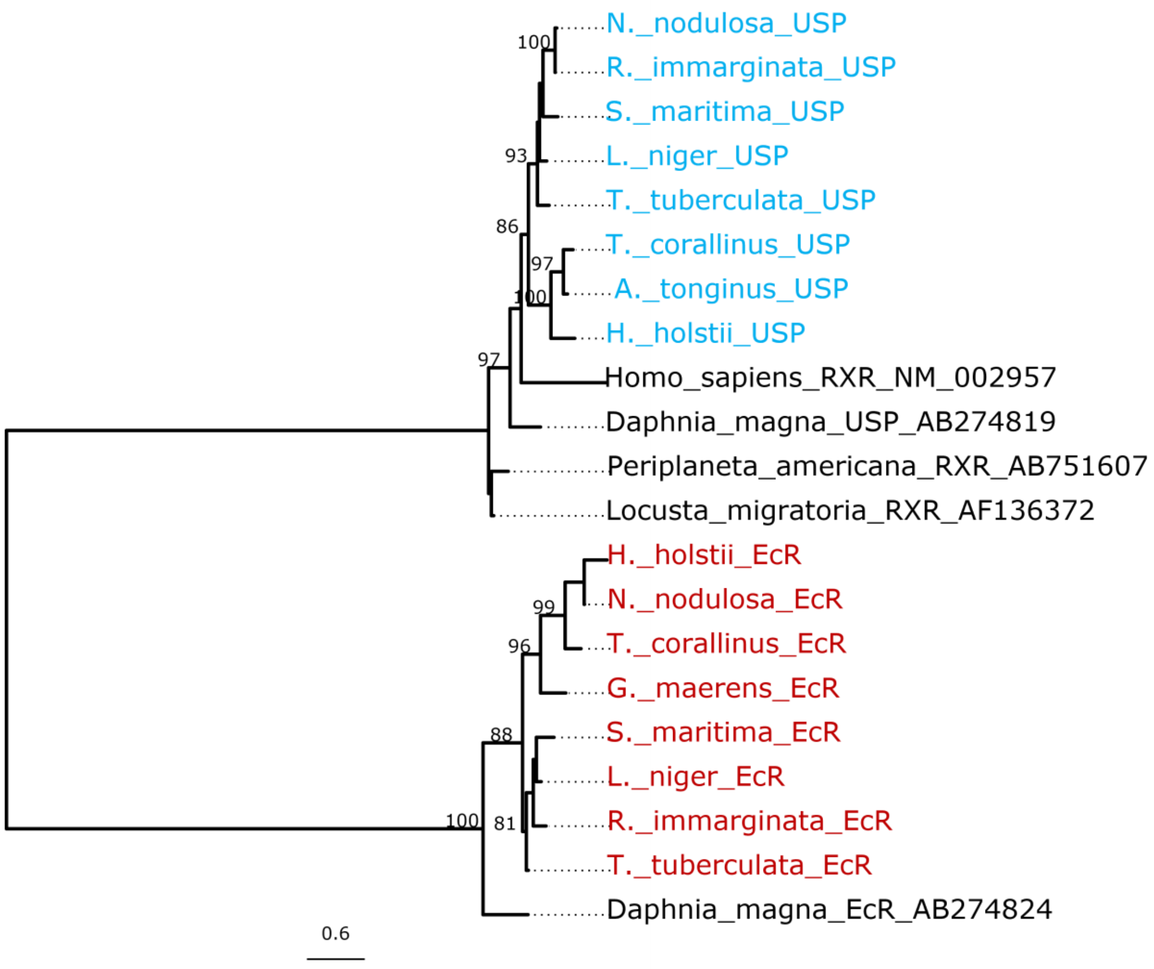

Supplementary Figure 47. Phylogenetic tree of ultraspiracle (USP) and ecdysteroid receptor (EcR). Tree is constructed with the maximum likelihood (ML) method with 1000 bootstrap replicates. Only bootstrap values larger than 80% are indicated for clarity.

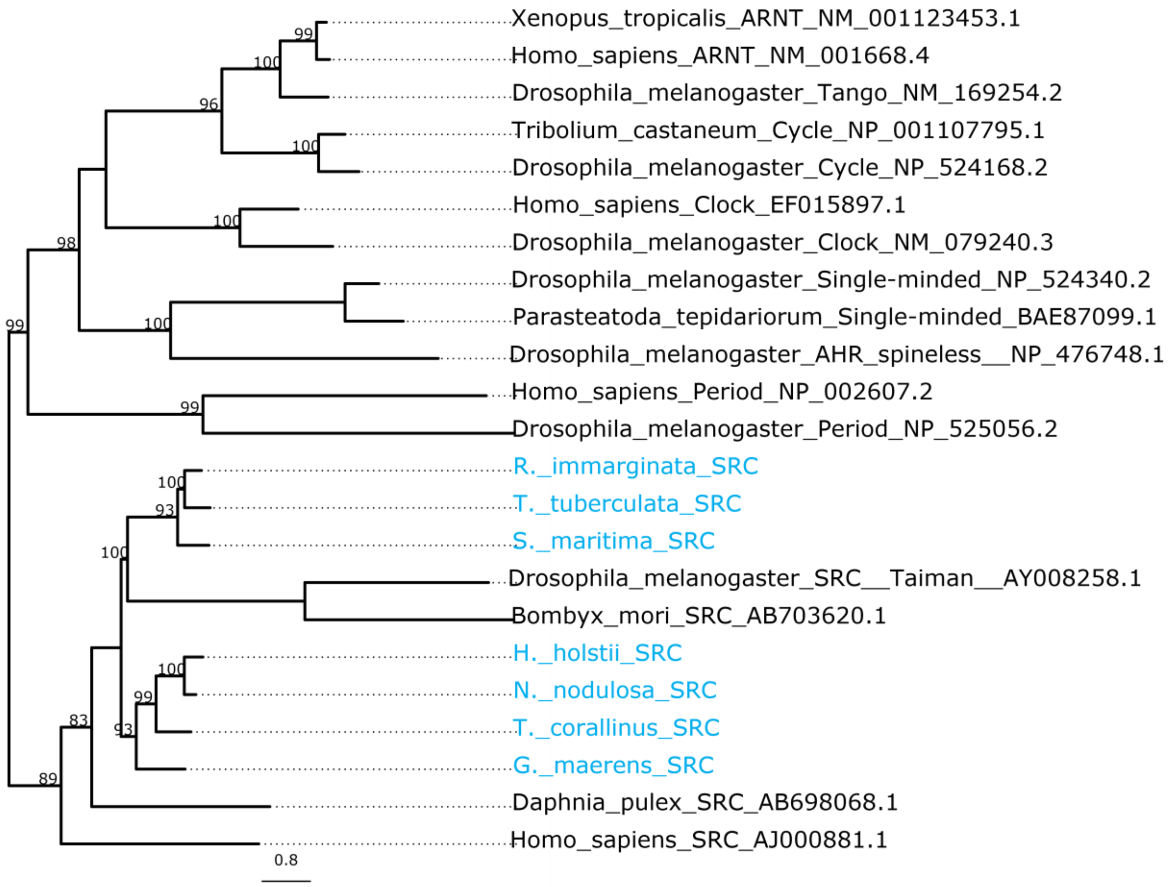

Supplementary Figure 48. Phylogenetic tree of steroid receptor coactivator (SRC). Tree is constructed with the maximum likelihood (ML) method with 1000 bootstrap replicates. Only bootstrap values larger than 80% are indicated for clarity. Tree rooted using other bHLH-PAS containing family proteins.

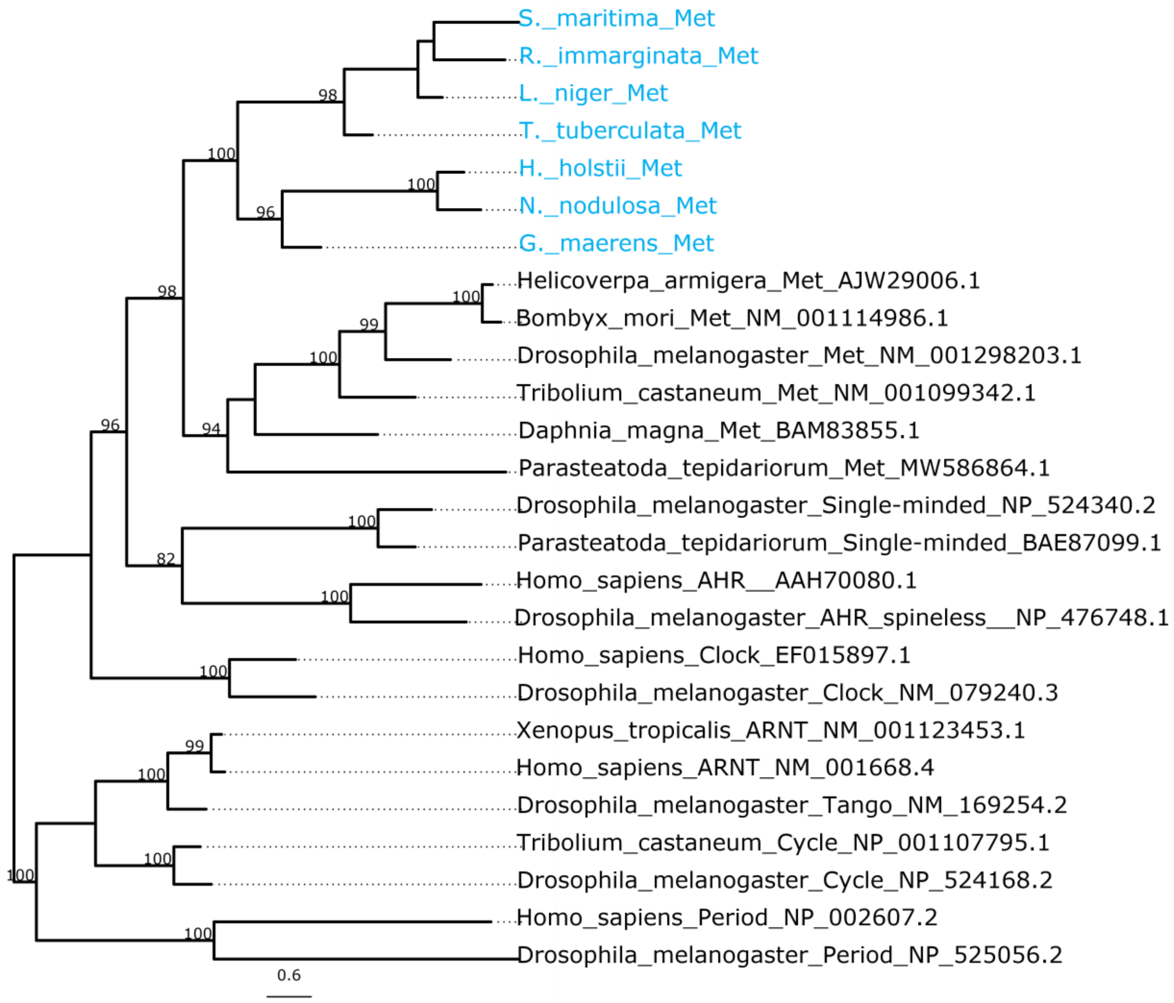

Supplementary Figure 49. Phylogenetic tree of methoprene receptor (Met). Tree is constructed with the maximum likelihood (ML) method with 1000 bootstrap replicates. Only bootstrap values larger than 80% are indicated for clarity. Tree rooted using other bHLH-PAS containing family proteins.

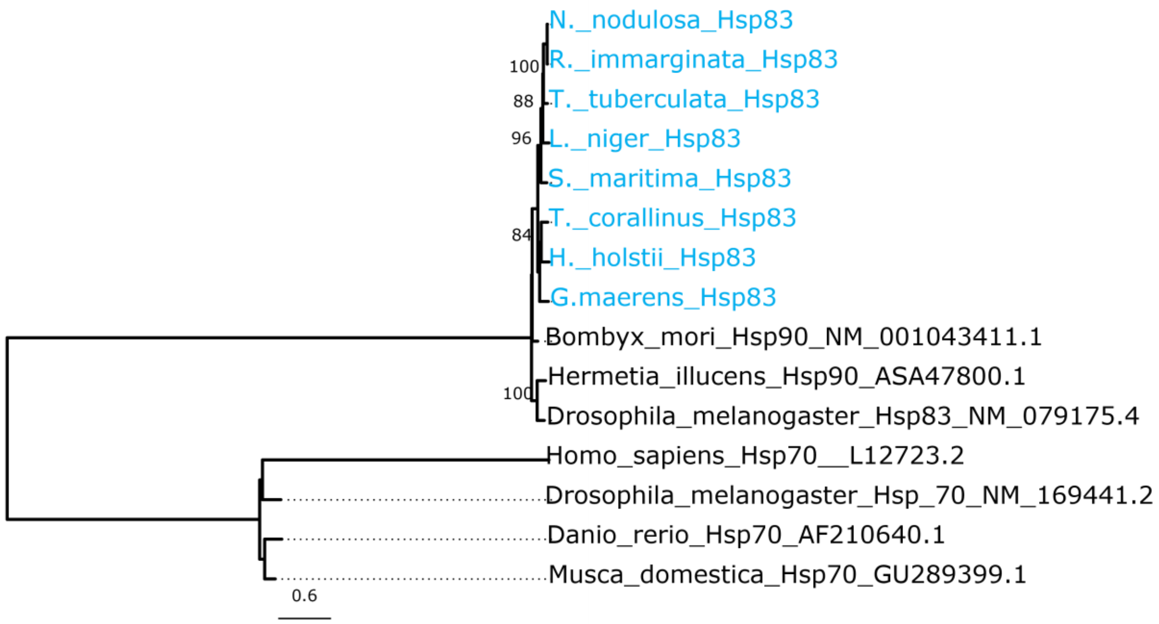

Supplementary Figure 50. Phylogenetic tree of heat shock protein 83 (Hsp83). Tree is constructed with the maximum likelihood (ML) method with 1000 bootstrap replicates. Only bootstrap values larger than 80% are indicated for clarity. Tree rooted using heat shock protein 70 (Hsp70).

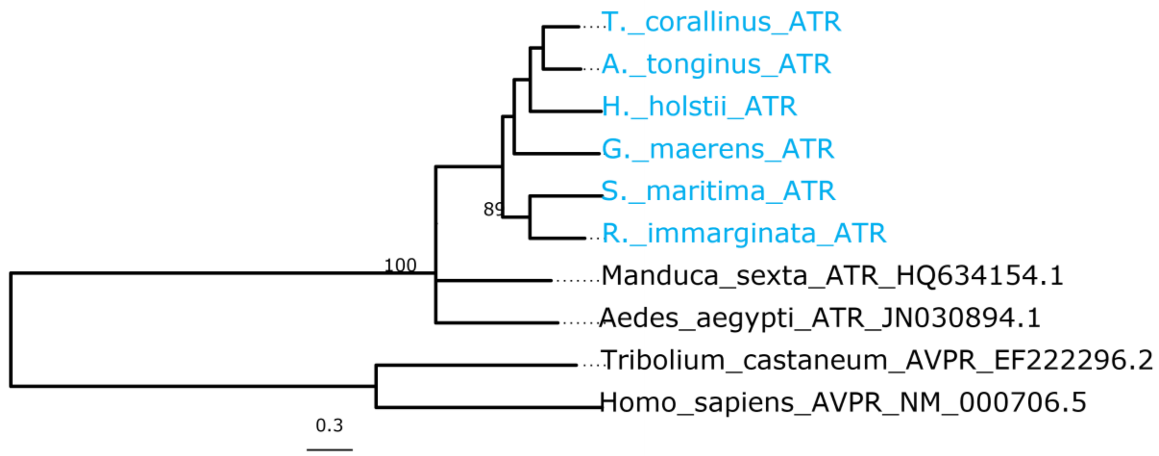

Supplementary Figure 51. Phylogenetic tree of allatotropin receptor (ATR). Tree is constructed with the maximum likelihood (ML) method with 1000 bootstrap replicates. Only bootstrap values larger than 80% are indicated for clarity. Tree rooted using metazoan arginine vasopressin receptor 1A (AVPR).

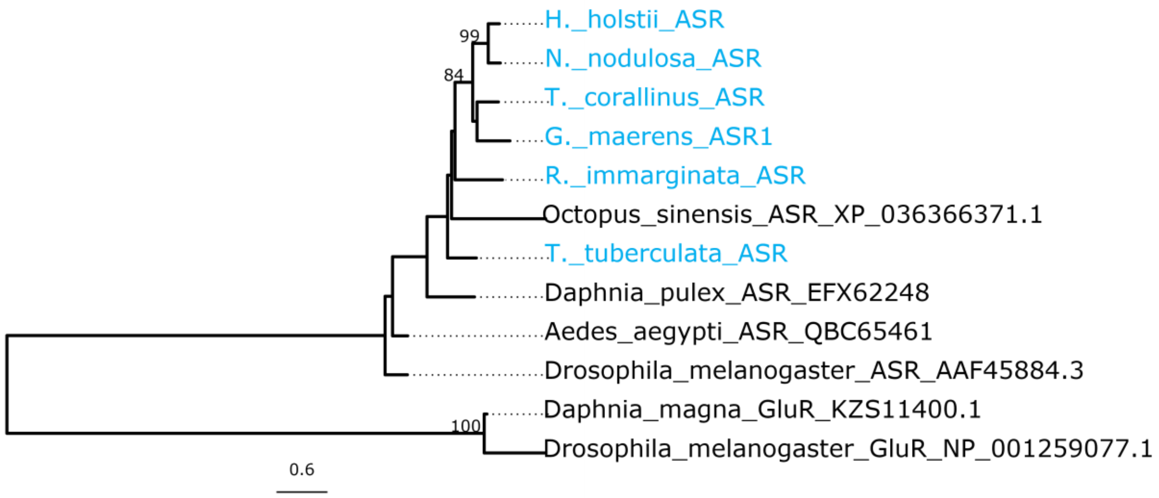

Supplementary Figure 52. Phylogenetic tree of allatostatin receptor (ASR). Tree is constructed with the maximum likelihood (ML) method with 1000 bootstrap replicates. Only bootstrap values larger than 80% are indicated for clarity. Tree rooted using metazoan glutamate receptor (GluR).

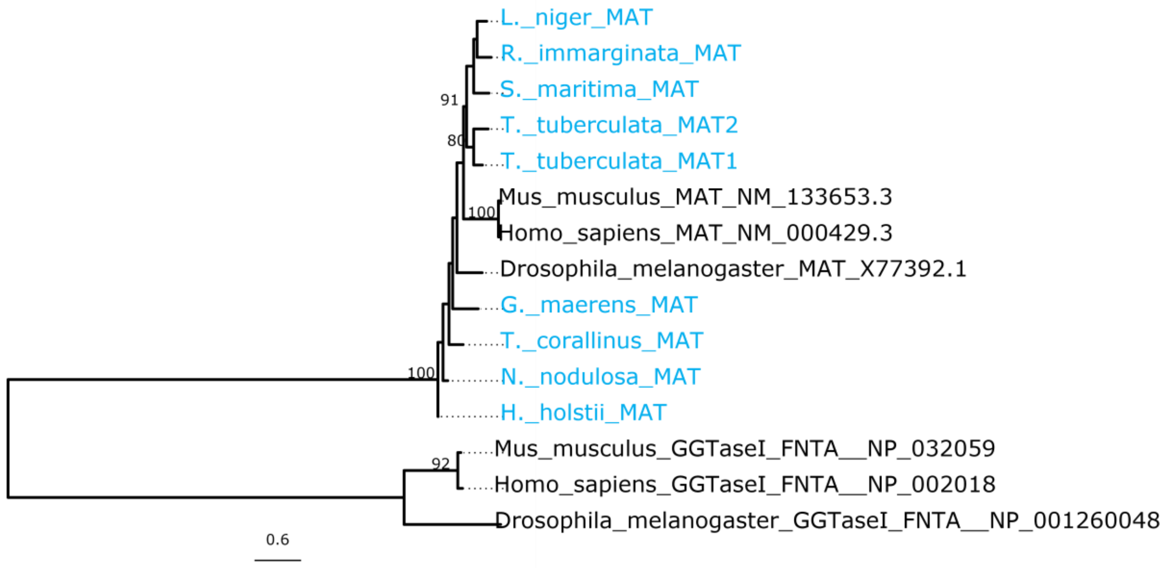

Supplementary Figure 53. Phylogenetic tree of S-adenosylmethionine synthase (MAT). Tree is constructed with the neighbor-Joining (NJ) method with 1000 bootstrap replicates. Only bootstrap values larger than 80% are indicated for clarity. Tree rooted using metazoan geranylgeranyltransferase (FNTA). The evolutionary distances were computed using the Poisson correction method and are in the units of the number of amino acid substitutions per site.

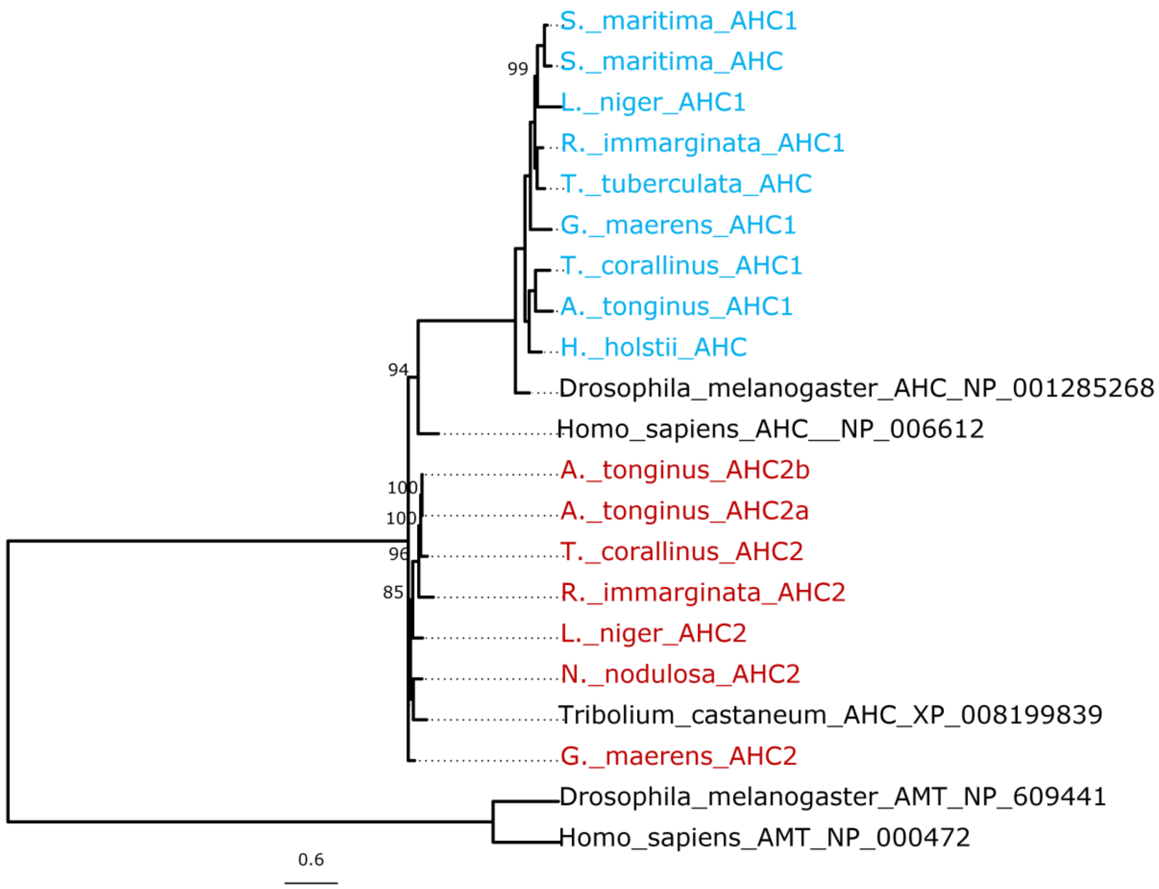

Supplementary Figure 54. Phylogenetic tree of S-adenosylhomocysteinase (AHC). Tree is constructed with the maximum likelihood (ML) method with 1000 bootstrap replicates. Only bootstrap values larger than 80% are indicated for clarity. Tree rooted using metazoan aminomethyltransferase (AMT).

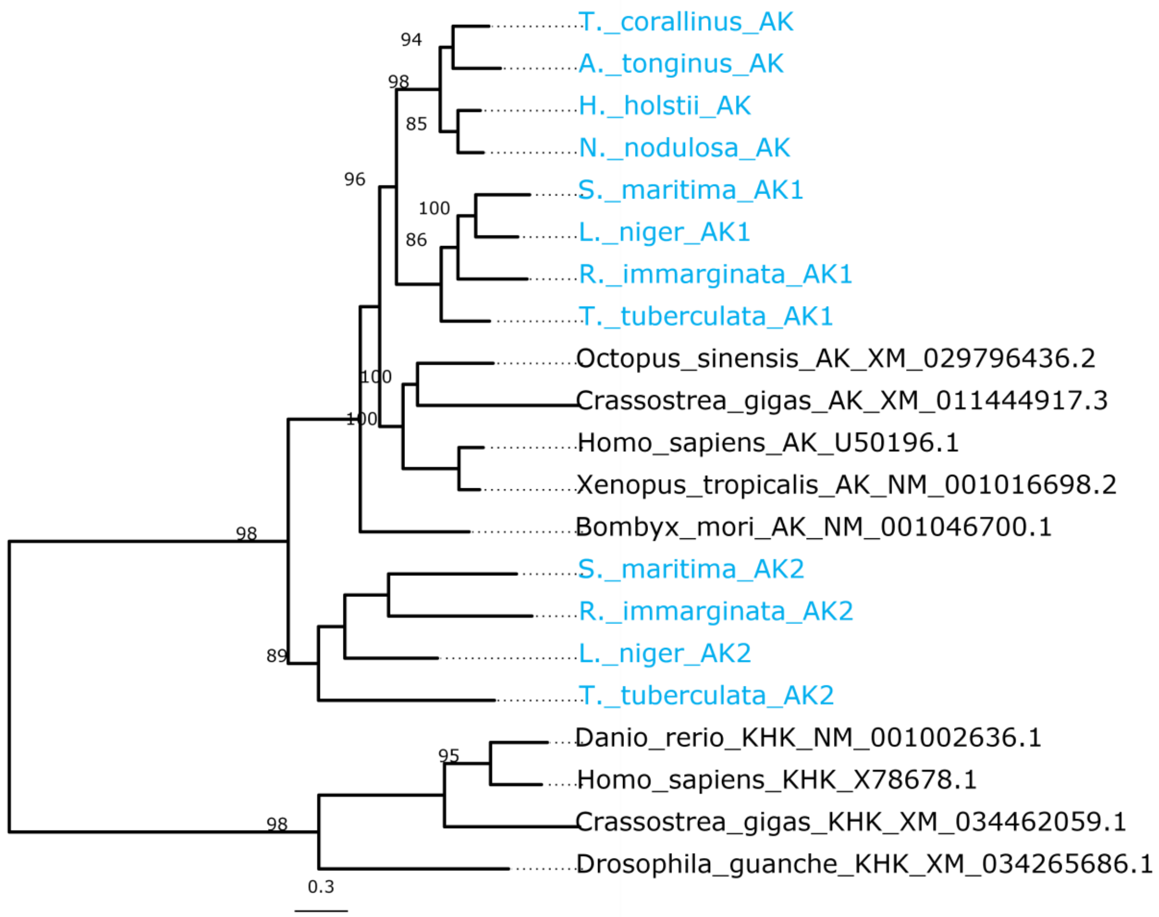

Supplementary Figure 55. Phylogenetic tree of adenosine kinase (AK). Tree is constructed with the maximum likelihood (ML) method with 1000 bootstrap replicates. Only bootstrap values larger than 80% are indicated for clarity. Tree rooted using metazoan ketohexokinase (KHK).

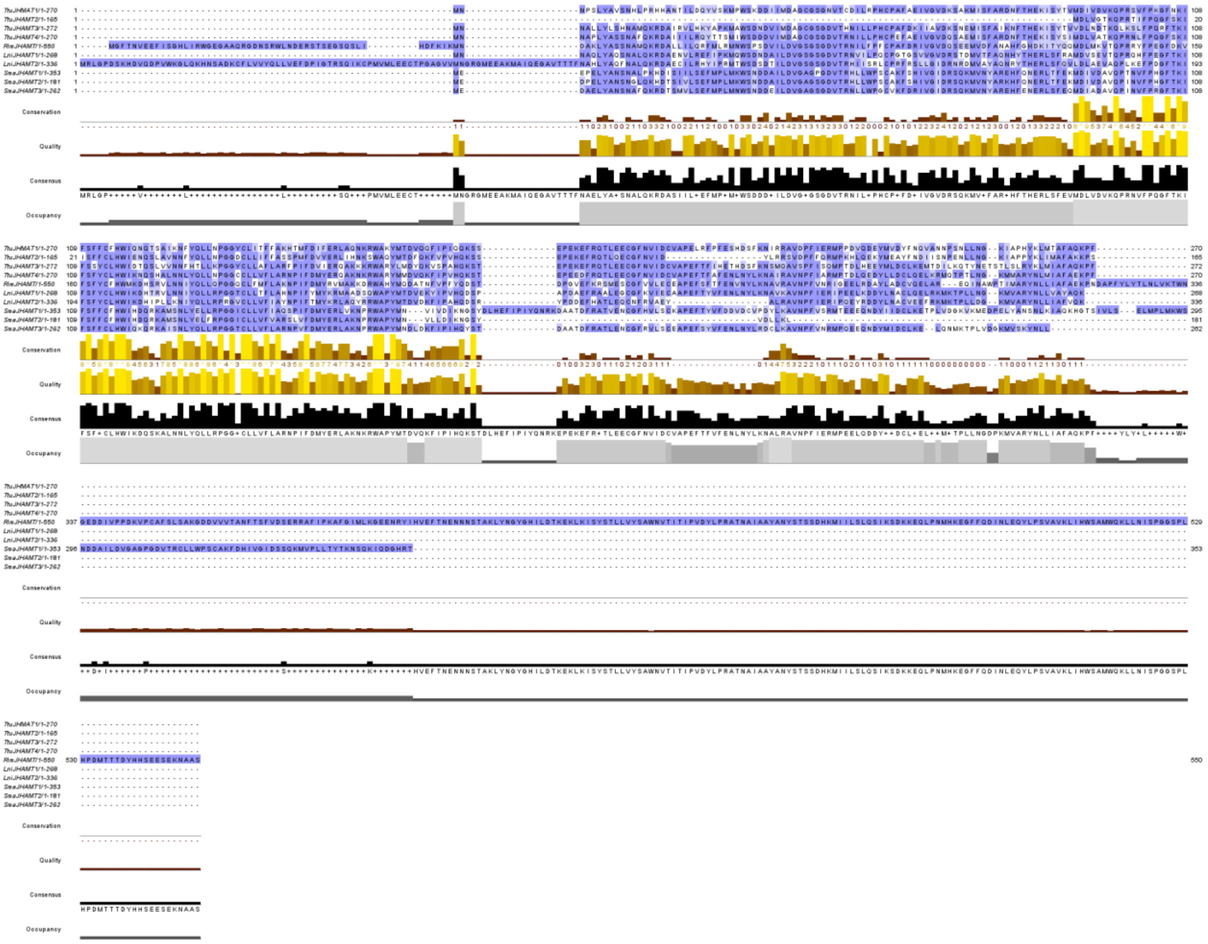

Supplementary Figure 56. Sequence alignment of centipede juvenile hormone acid methyltransferase (JHAMT). Alignment shows a highly conserved S-adenosyl methionine (SAM) binding domain among the genes. Ttu, *T. tuberculata*; Rim, *R. immarginata*; Lni, *L. niger*; Sma, *S. maritima*.

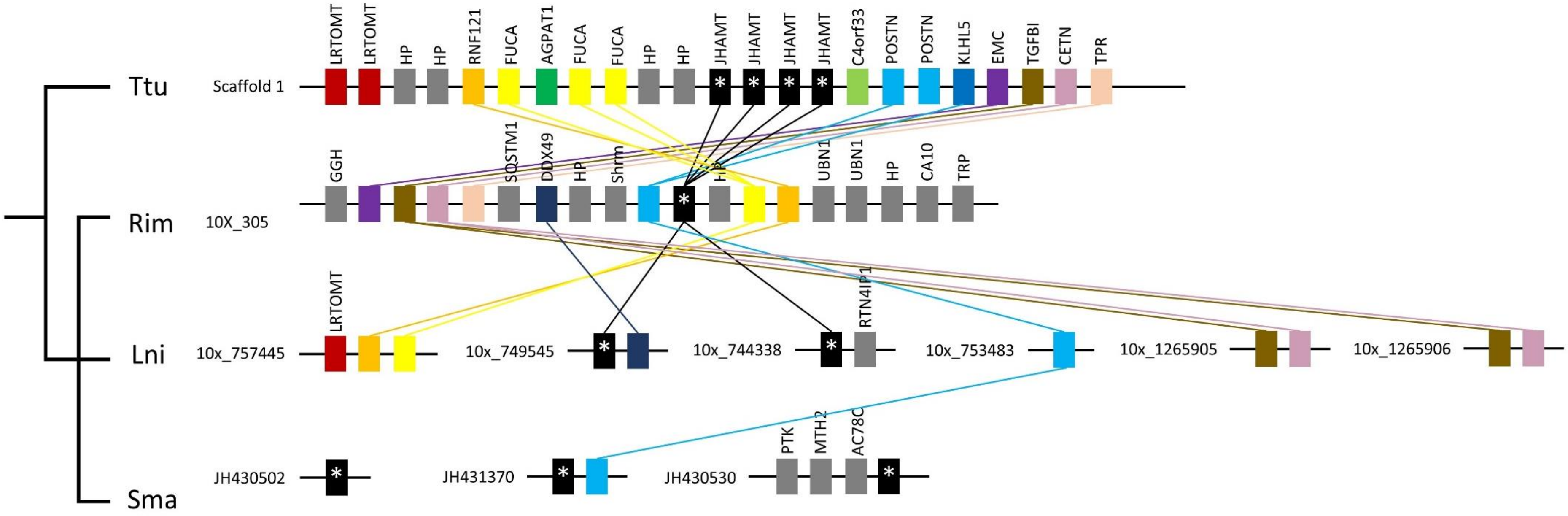

Supplementary Figure 57. Microsteny of JHAMT in centipede genomes. Ttu, *T. tuberculate*; Rim, *R. immarginata*; Lni, *L. niger*; Sma, *S. maritima*; LRTOMT, leucine-rich repeat-containing protein 51; HP, hypothetical protein; RNF121, RING finger protein 121; FUCA, alpha-L-fucosidase; AGPAT1, 1-acyl-sn-glycerol-3-phosphate acyltransferase alpha; JHAMT, juvenile hormone acid O-methyltransferase; C4orf33, UPF0462 protein C4orf33 homolog; POSTN, periostin; KLHL5, kelch-like protein 5; EMC, ER membrane protein complex subunit; TGFBI, transforming growth factor-beta-induced protein ig-h3; CETN, centrin; TPR, tetratricopeptide repeat protein; GGH, gamma-glutamyl hydrolase; SQSTM1, sequestosome-1; DDX49, ATP-dependent RNA helicase DDX49; Shrm, protein Shroom; UBN1, ubinuclein; CA10, carbonic anhydrase-related protein 10; TRP, transient-receptor-potential-like protein, RTN4IP1, reticulon-4-interacting protein 1 homolog, mitochondrial-like; PTK, tyrosine-protein kinase; MTH2, G-protein coupled receptor Mth2; AC78C, adenylyl cyclase 78C/adenylate cyclase type 8.

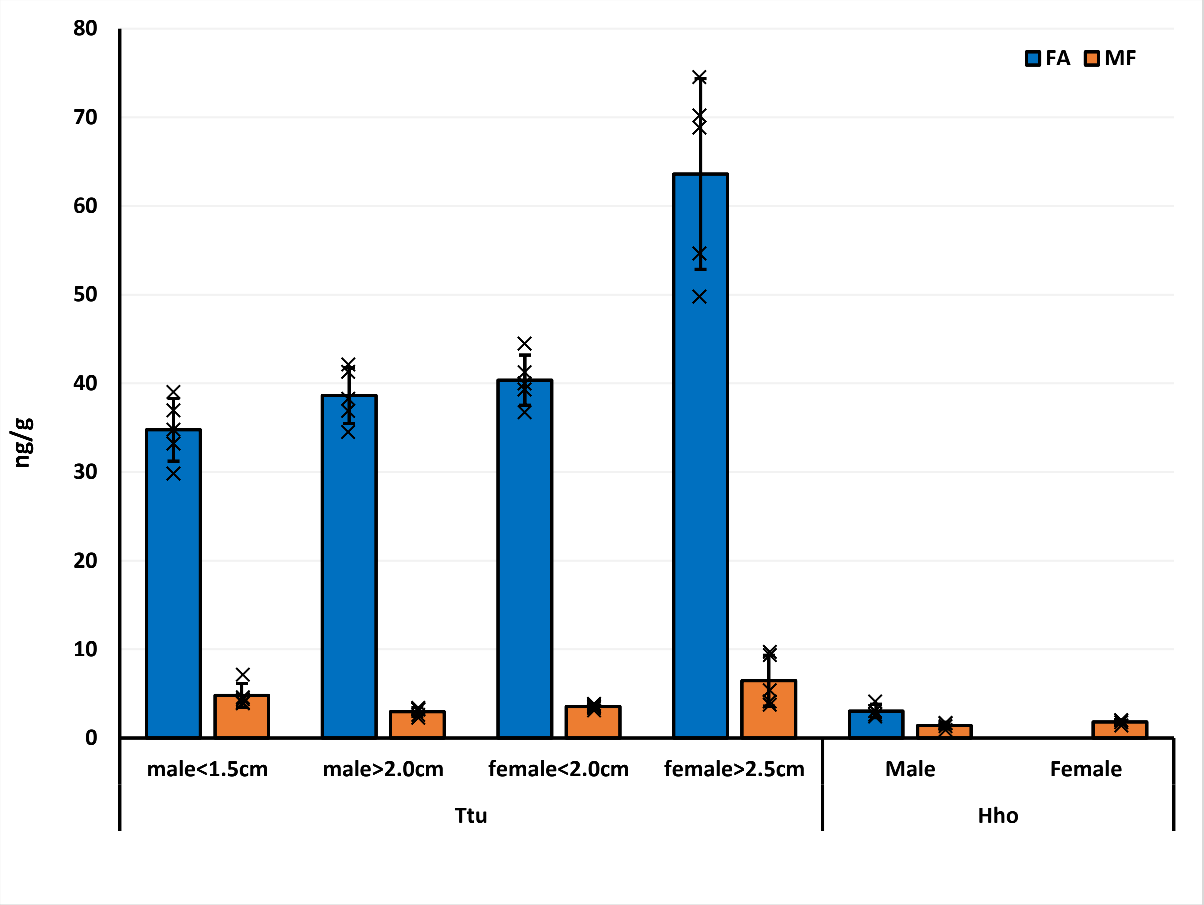

Supplementary Figure 58. Hormone measurement of sesquiterpenoids farnesoic acid and methyl farnesoate, in biologically independent adult *T. tuberculata* (male<1.5cm, n=5; male>2.0cm, n=5; female<2.0cm, n=5; female>2.5cm, n=5) and *H. holstii* (male, n=4; female, n=4). Ttu, *T. tuberculata*; Hho, *H. holstii*. Data are presented as mean values +/- SEM. Individual data points are present on each bar. The vertical error bars indicate the standard error of mean (SEM) among the data points in each experiment.

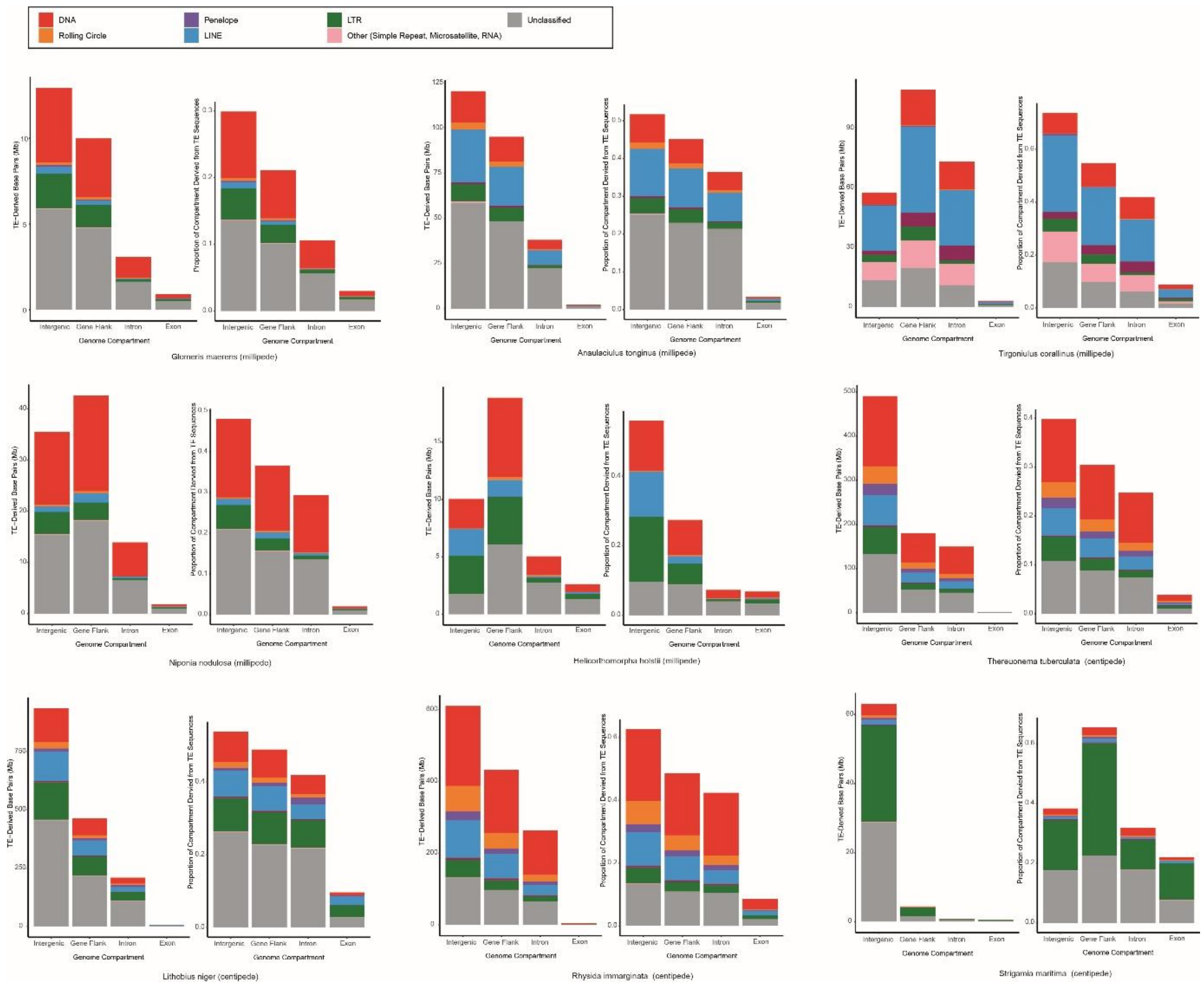

Supplementary Figure 59. Myriapod repeat location plots. Left-hand plots indicate the amount of each repeat class identified in each genomic compartment in base pairs (bp). Right-hand plots indicate the relative proportion of each genomic compartment occupied by each repeat type.

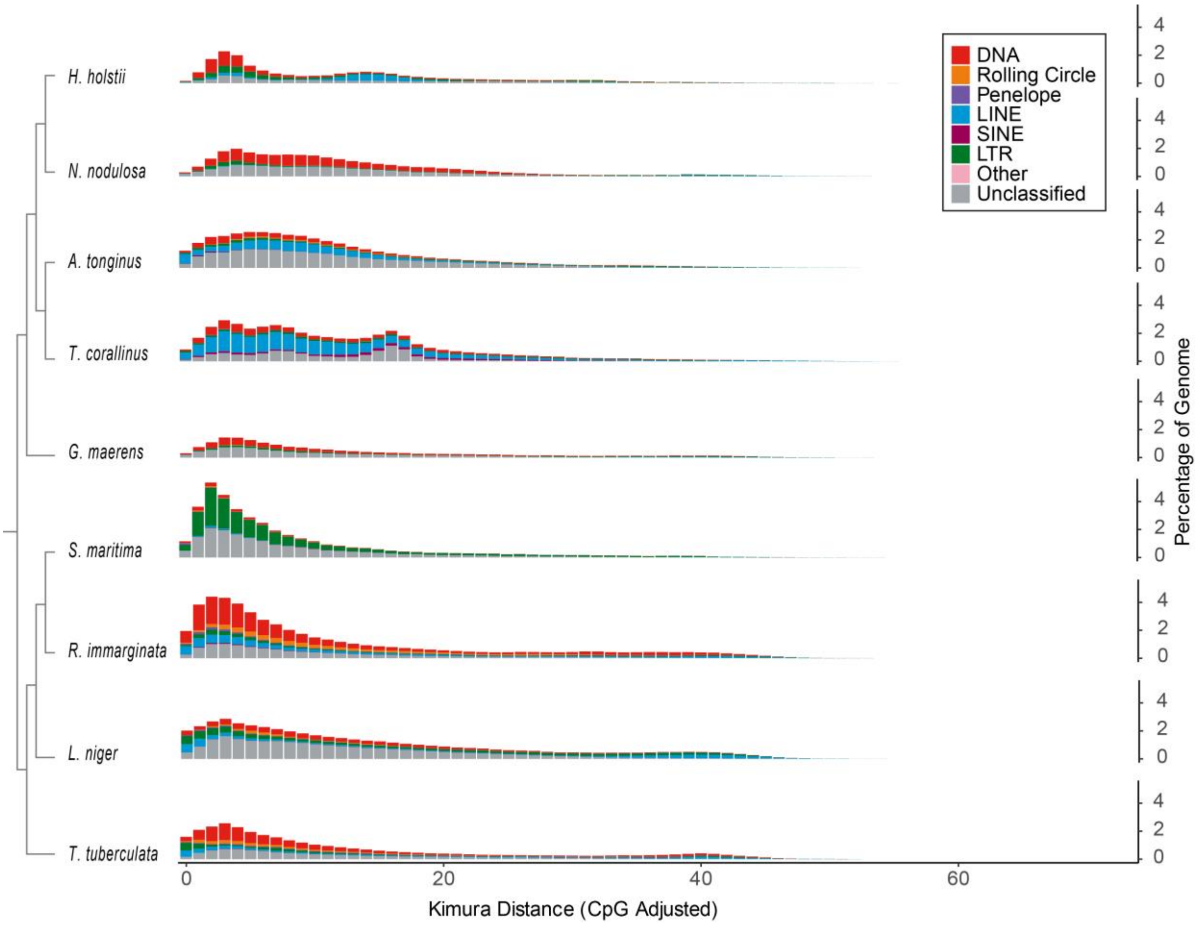

Supplementary Figure 60. Myriapod repeat landscape plots.

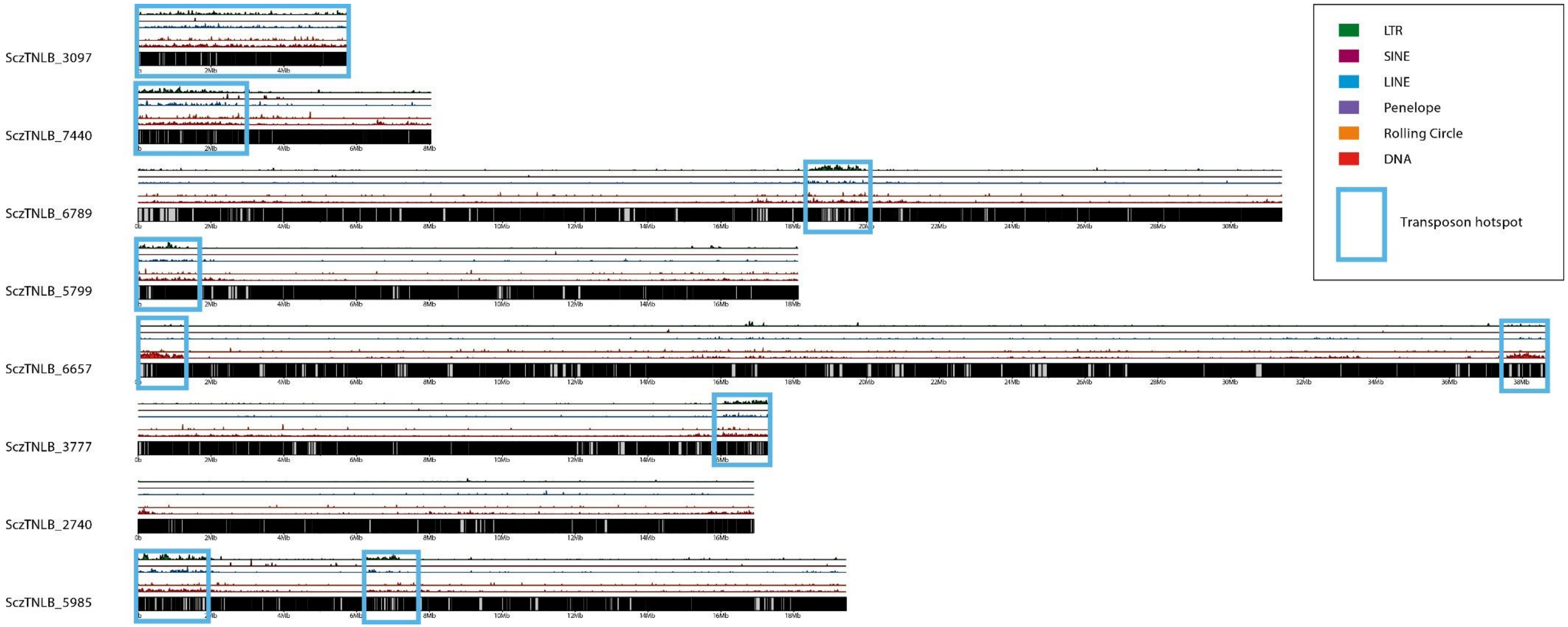

Supplementary Figure 61. Repeat density karyotype plot for *Helicorthomorpha holstii.*

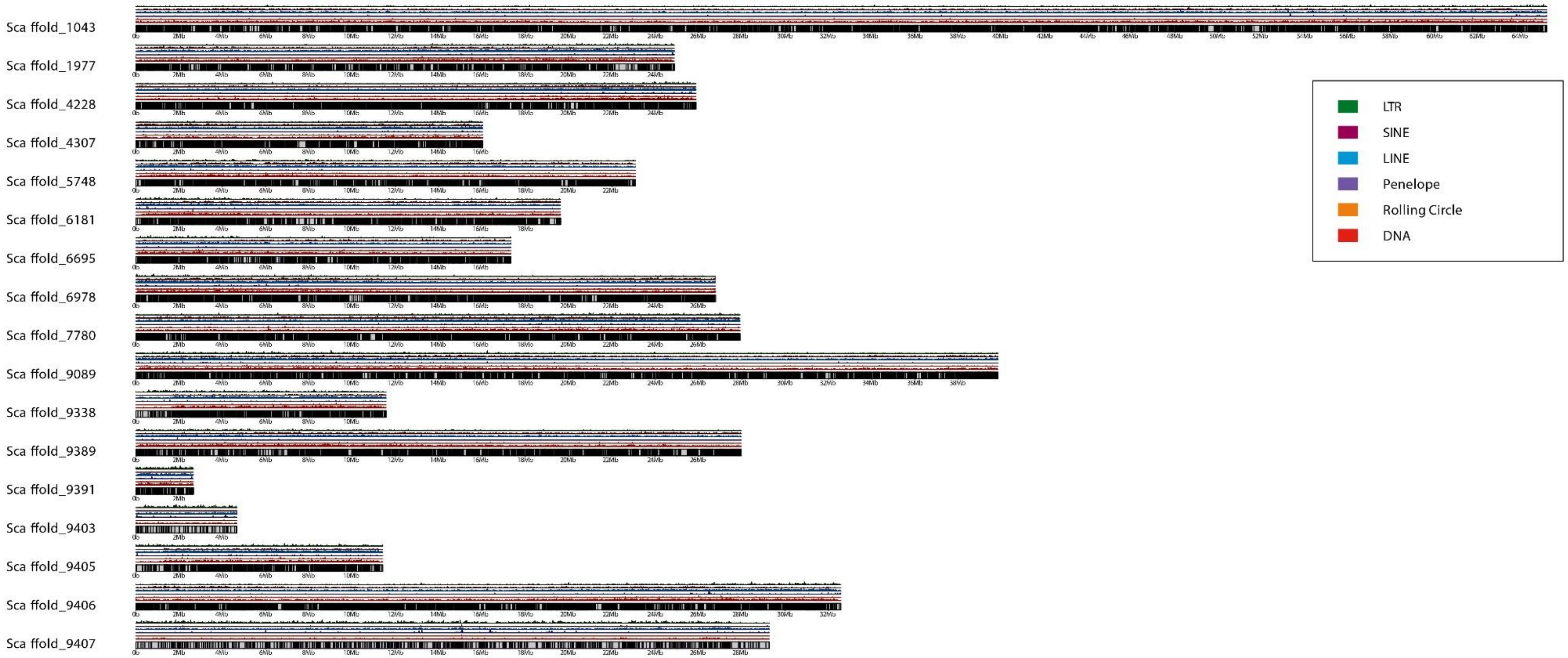

Supplementary Figure 62. Repeat density karyotype plot for *Trigoniulus corallinus*.

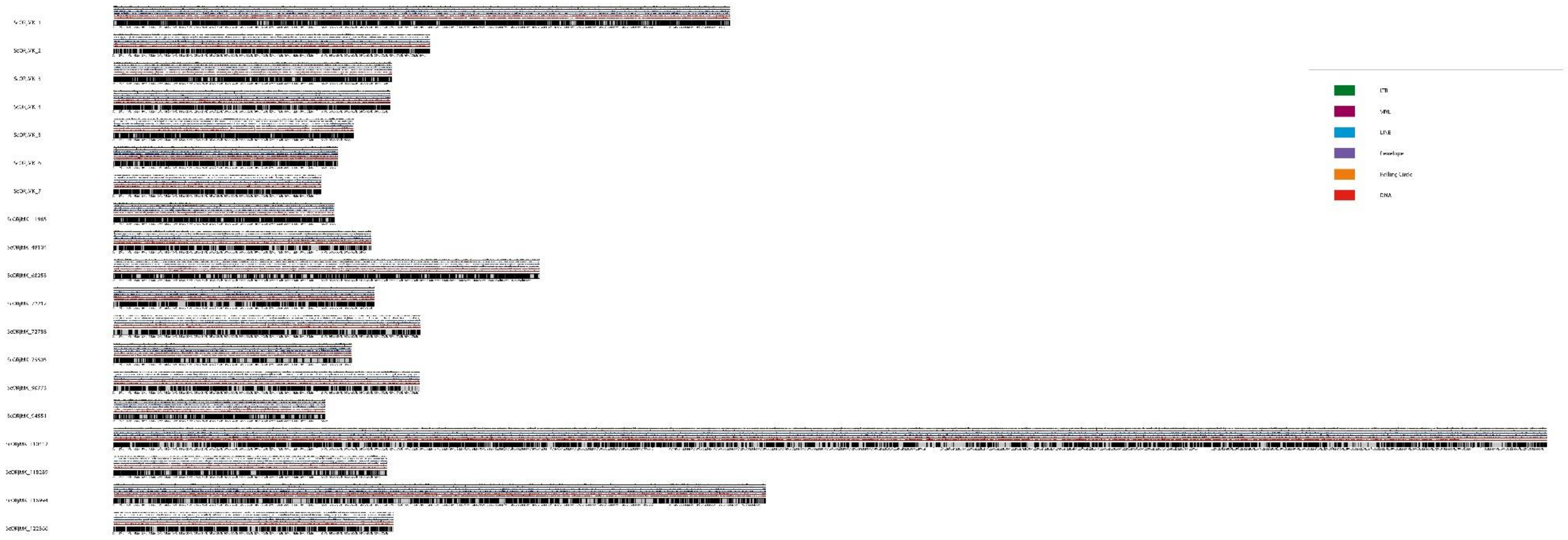

Supplementary Figure 63. Repeat density karyotype plot for *Thereuonema tuberculata*.

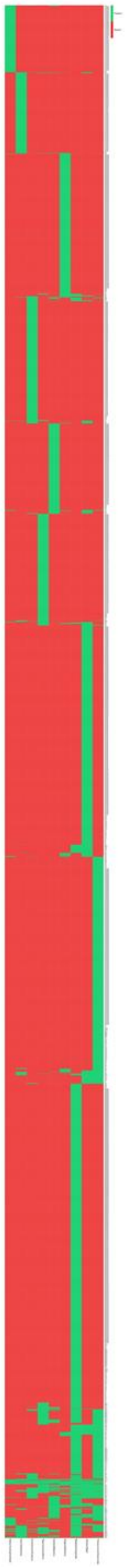

Supplementary Figure 64. Bar chart showing the pattern by which different myriapod species share individual TE families. Original figure can be found in Figshare: "sharedRepeatsSetmap_page-0001.zip" https://doi.org/10.6084/m9.figshare.15088722

Supplementary Discussion - Genome size and transposable element content

The transposable elements (TEs) of Myriapoda are poorly described. Here, we provide TE annotations, TE libraries, and a comparative TE analysis for nine genomes from across myriapod diversity (four centipedes, and five millipedes) to describe the abundance, diversity, and genomic distribution of myriapod TEs. We identify considerable variability in the proportion of the genome composed of TEs in myriapods, ranging from 17.84% in the millipede *G. maerens,* to 51.67% in the millipede *T. corallinus,* as well as the relative quantity of TEs present in myriapod genomes, ranging from 26.74Mb in the millipede *G. maerens,* to 1609.95Mb in the centipede *L. niger* (Supplementary Data 2). Overall, millipede genomes have a higher mean TE content (millipede $\bar{x}$ = 43.17%, centipede $\bar{x}$ = 31.86%), but centipede genomes have a much higher relative quantity of TEs (millipede $\bar{x}$ = 129.14Mb, centipede $\bar{x}$ = 945.78Mb).

Myriapod genomes contain representation from all major TE types except SINEs which are absent or only present <1% (except *T. corallinus* 3.43%). Retroelements comprise 3.00 – 26.69% of total genomic content, while DNA TEs comprise 2.05 – 20.34%, and rolling-circle elements comprise 0.17 – 5.22% (Supplementary Data 2). There is a relatively high proportion of unclassified repeats present in myriapod genomes (6.27 – 23.98%, Supplementary Data 2), as expected given that TEs are poorly explored in the group. DNA TEs and unclassified elements are often most abundant, while a few genomes instead show large proportions of LINEs (*A. tonginus, T. corallinus*) or LTR TEs (*L. niger*, *S. maritima*) (Supplementary Data 2, Figure 2A).

Considering TE distribution within myriapod genomes, most insertions occur in intergenic regions (non-coding regions >20Kb from annotated genes), except *S. maritima*, where the majority occur within gene flanks (<20kb from annotated genes) (Supplementary Figure 59-60). The genomic compartment with the second highest proportion of repeats is generally gene flanks (except *S. maritimia*). As expected, relatively few TE insertions are present within gene exons, but there is a much greater presence of TEs within gene introns in many genomes (Supplementary Figure 59-60).

For three myriapod genomes with chromosome-level assemblies, we were able to examine karyotype TE density plots (*H. holstii, T. tuberculata, T. corallinus,* Supplementary Figures 61-63). The karyotype plots for *T. tuberculata* and *T. corallinus* suggest a relatively even spread of TEs across chromosomes (Supplementary Figures 59-60 & 62-63). However,

the karyotype plot for *H. holstii* indicates the presence of several TE hotspots: SczTNLB_3097, high abundance of all TEs except SINEs over the entire short chromosome; SczTNLB_7440, high abundance of all TEs except SINEs at the start (0-2Mb); SczTNLB_6789, dense cluster of LINEs, LTR TEs, and DNA TEs off-centre (18-20Mb); SczTNLB_5799, cluster of all TEs except SINEs at the start (0-2Mb); SczTNLB_6657, dense clusters of DNA TEs at the start (0-1Mb) and end (38Mb); SczTNLB_3777, cluster of all TEs except SINEs at the end (16Mb); SczTNLB_2740, cluster of DNA TEs at the start (0Mb) and end (16Mb); SczTNLB_5985, dense cluster of all TEs except SINEs at the start (0Mb), and off-centre (7Mb) (Supplementary Figure 61). It is currently unclear what drives the accummulations of TEs in genomic hotspots in the millipede *H. holstii* compared to the millipede *T. coralinus* and the centipede *T. tuberculata*.
