# Supplementary material for: Myriapod genomes reveal ancestral horizontal gene transfer and hormonal gene loss in millipedes
Source: Nat Commun. 2022 May 30;13:3010. doi: 10.1038/s41467-022-30690-0 (PMC9151784; doi:10.1038/s41467-022-30690-0)
Supplement: Supplementary file 3 — Description of Additional Supplementary Files [file 41467_2022_30690_MOESM3_ESM.pdf]

Supplementary Data

Supplementary Data 1 – Next generation sequencing (NGS) Data

Supplementary Data 2 – Transposable elements (TEs) analysis

Supplementary Data 3 – Primer information used in this study

Supplementary Data 4 – Gene gain and loss annotated by Gene ontology (GO)

Supplementary Data 5 - Gene gain and loss annotated by EuKaryotic Orthologous Groups (KOG)

Supplementary Data 6 - Gene gain and loss annotated by Kyoto Encyclopedia of Genes and Genomes (KEGG)

Supplementary Data 7 – ANTP class gene annotations

Supplementary Data 8 – Microsynteny of glucose/arabinose dehydrogenase (GDH; WP_189008864.1)

Supplementary Data 9 - Microsynteny of glucose/arabinose dehydrogenase (GDH; WP_146884959.1)

Supplementary Data 10 - Microsynteny of non-ribosomal peptide synthetase (NRPS; WP_096595152.1)

Supplementary Data 11 - Microsynteny of glycoside hydrolase family 16 protein (GH16; AQQ75061.1)

Supplementary Data 12 – Microsynteny of hydrolase family 16 protein (GH16; WP_052600908.1)

Supplementary Data 13 - Microsynteny of efflux RND transporter permease subunit (WP_163176792.1)

Supplementary Data 14 - Microsynteny of NADH dehydrogenase (WP_034862301.1)

Supplementary Data 15 - Microsynteny of AzlD domain-containing protein (WP_095524423.1)

Supplementary Data 16 - Microsynteny of anaerobic sulfatase maturase (SBW02910.1)

Supplementary Data 17 - Microsynteny of alpha-2-macroglobulin (OYY43986.1)

Supplementary Data 18 - Microsynteny of SYLF domain-containing protein (RPH48231.1)

Supplementary Data 19 - Sesquiterpenoid hormone genes annotation in *T. tuberculata*

Supplementary Data 20 - Sesquiterpenoid hormone genes annotation in *R. immarginata*

Supplementary Data 21 - Sesquiterpenoid hormone genes in *L. niger*

Supplementary Data 22 - Sesquiterpenoid hormone genes annotation in *S. maritima*

Supplementary Data 23 - Sesquiterpenoid hormone genes annotation in *G. maerens*

Supplementary Data 24 - Sesquiterpenoid hormone genes annotation in *N. nodulosa*

Supplementary Data 25 - Sesquiterpenoid hormone genes annotation in *A. tonginus*

Supplementary Data 26 - Sesquiterpenoid hormone genes annotation in *H. holstii*

Supplementary Data 27 - Sesquiterpenoid hormone genes annotation in *T.corallinus*

Supplementary Data 28 - Centipede *JHAMT* microsynteny data
