# Supplementary material for: Myriapod genomes reveal ancestral horizontal gene transfer and hormonal gene loss in millipedes
Source: Nat Commun. 2022 May 30;13:3010. doi: 10.1038/s41467-022-30690-0 (PMC9151784; doi:10.1038/s41467-022-30690-0)
Supplement: Supplementary file 5 — Reporting Summary [file 41467_2022_30690_MOESM5_ESM.pdf]

nature portfolio

Corresponding author(s): Jerome H.L. Hui

Last updated by author(s): May 4, 2022

Reporting Summary

Nature Portfolio wishes to improve the reproducibility of the work that we publish. This form provides structure for consistency and transparency in reporting. For further information on Nature Portfolio policies, see our Editorial Policies and the Editorial Policy Checklist.

Statistics

For all statistical analyses, confirm that the following items are present in the figure legend, table legend, main text, or Methods section.

n/a	Confirmed	
☐	☒	The exact sample size (*n*) for each experimental group/condition, given as a discrete number and unit of measurement
☐	☒	A statement on whether measurements were taken from distinct samples or whether the same sample was measured repeatedly
☐	☒	The statistical test(s) used AND whether they are one- or two-sided<br>*Only common tests should be described solely by name; describe more complex techniques in the Methods section.*
☒	☐	A description of all covariates tested
☒	☐	A description of any assumptions or corrections, such as tests of normality and adjustment for multiple comparisons
☐	☒	A full description of the statistical parameters including central tendency (e.g. means) or other basic estimates (e.g. regression coefficient) AND variation (e.g. standard deviation) or associated estimates of uncertainty (e.g. confidence intervals)
☐	☒	For null hypothesis testing, the test statistic (e.g. *F*, *t*, *r*) with confidence intervals, effect sizes, degrees of freedom and *P* value noted<br>*Give P values as exact values whenever suitable.*
☒	☐	For Bayesian analysis, information on the choice of priors and Markov chain Monte Carlo settings
☒	☐	For hierarchical and complex designs, identification of the appropriate level for tests and full reporting of outcomes
☐	☒	Estimates of effect sizes (e.g. Cohen's *d*, Pearson's *r*), indicating how they were calculated

Our web collection on statistics for biologists contains articles on many of the points above.

Software and code

Policy information about availability of computer code

Data collection	Illumina, HiseqXten (Chromium WGS), Omni-C
Data analysis	alienness, version 2, link http://alienness.sophia.inra.fr/cgi/index.cgi<br>assembly-stats, version 1.0.1, link https://github.com/rjchallis/assembly-stats<br>AUGUSTUS, verion 3.3, link http://bioinf.uni-greifswald.de/augustus/binaries/old/augustus-3.3.tar.gz<br>blast+, version 2.2.31+, link https://ftp.ncbi.nlm.nih.gov/blast/executables/blast+/2.2.31/<br>BUSCO, version 5, link https://gitlab.com/ezlab/busco<br>bwa, version 2.4.2, link https://github.com/lh3/bwa<br>CAFE5, version 5.0.0, link https://github.com/hahnlab/CAFE5/releases/tag/v5.0<br>clusterProfiler, version 3.16.1, link https://bioconductor.org/packages/release/bioc/html/clusterProfiler.html<br>cutadapt, version 1.10, link https://github.com/marcelm/cutadapt/tree/v1.10<br>DSK, version 2.1.0, link http://gatb-tools.gforge.inria.fr/versions/bin/dsk-2.1.0-Linux.tar.gz<br>eggnog-mapper, version 2.1.7, link https://github.com/eggnogdb/eggnog-mapper/releases/tag/2.1.7<br>FastQC, version 0.11.8, link https://www.bioinformatics.babraham.ac.uk/projects/fastqc/fastqc_v0.11.8.zip<br>funannotate, version 1.7.4, link https://funannotate.readthedocs.io/en/latest/<br>genometools, version 1.5.8, link http://genometools.org/pub/genometools-1.5.8.tar.gz<br>gggenes, version 0.4.1, link https://cran.r-project.org/web/packages/gggenes/index.html<br>ggplot2, version 3.3.5, link https://cran.r-project.org/web/packages/ggplot2/index.html<br>ggtree, version 3.2.1, link https://bioconductor.org/packages/release/bioc/html/ggtree.html<br>InterProScan, version 5.26-65.0, link https://github.com/ebi-pf-team/interproscan/wiki/Interproscan5_26_65_ReleaseNotes<br>iqtree, version 2.2.0, link http://www.iqtree.org<br>KAT, version 2.1.1, link https://github.com/TGAC/KAT<br>Kraken2, version 2.0, link https://github.com/DerrickWood/kraken

March 2021

MAFFT，version 7.453, link https://mafft.cbrc.jp/alignment/software/source.html
MEGA, version 7, link https://www.megasoftware.net/
minion, version 15-065, link http://wwwdev.ebi.ac.uk/enright-dev/kraken/reaper/src/reaper-15-065.tgz
mirDeep2, version 2_0_0_8, link https://github.com/rajewsky-lab/mirdeep2
MITE_Hunter, version 11-2011, link http://target.iplantcollaborative.org/mite_hunter/MITE%20Hunter-11-2011.zip
MUSCLE, version 3.8.31, link https://www.drive5.com/muscle/downloads.htm
OrthoFinder, version 2.5.2, link https://github.com/davidemms/OrthoFinder/releases/tag/2.5.2
OrthoMCL, version 2.0.9, http://orthomcl.org/common/downloads/software/v2.0/orthomclSoftware-v2.0.9.tar.gz
PAML, version 3.1, link http://abacus.gene.ucl.ac.uk/software/paml.html
PASA, version 2.1.0, link https://github.com/PASApipeline/PASApipeline
pathview, version 1.34.0, link https://bioconductor.org/packages/release/bioc/html/pathview.html
Prokka, version 1.12-beta, link https://github.com/tseemann/prokka
ProtTest, version 3.4, link https://github.com/ddarriba/prottest3
R, version 3.6.3, link https://cran.r-project.org/src/base/R-3/
R, version 4.1.0, link https://cran.r-project.org/
r8s, version 1.8.1, link https://sourceforge.net/projects/r8s/files/r8s1.81.tar.gz
RaxML, version 8.2.4, link https://github.com/stamatak/standard-RAxML
RECON, version 1.08, link http://www.repeatmasker.org/RepeatModeler/RECON-1.08.tar.gz
RepeatModeler, version 1.0.11, link http://www.repeatmasker.org/RMDownload.html
Supernova, version 2.1.1, link https://github.com/major/supernova/archive/v2.1.0.tar.gz
SyMAP, version 4.2, link http://www.agcol.arizona.edu/software/symap/index.html
TransDecoder, version 5.3.0, link https://github.com/TransDecoder/TransDecoder
TransposonPSI, version 08222010, link https://sourceforge.net/projects/transposonpsi/files/TransposonPSI_08222010.tgz/download
trimAl, version 1.4, link https://github.com/scapella/trimal
Trinity, version 2.9.1, link https://github.com/trinityrnaseq/trinityrnaseq
Custom codes, link https://github.com/xieyichun50/Myriapod-genomes

For manuscripts utilizing custom algorithms or software that are central to the research but not yet described in published literature, software must be made available to editors and reviewers. We strongly encourage code deposition in a community repository (e.g. GitHub). See the Nature Portfolio guidelines for submitting code & software for further information.

Data

Policy information about availability of data

All manuscripts must include a data availability statement. This statement should provide the following information, where applicable:

- Accession codes, unique identifiers, or web links for publicly available datasets
- A description of any restrictions on data availability
- For clinical datasets or third party data, please ensure that the statement adheres to our policy

The final assemblies were submitted to NCBI Assembly under accession numbers WWPM00000000 (Glomeris maerens) [https://www.ncbi.nlm.nih.gov/nuccore/WWPM00000000], JAAFCF000000000 (Helicorthomorpha holstii) [https://www.ncbi.nlm.nih.gov/nuccore/JAAFCF000000000], WWPL00000000 (Anaulaciulus tonginus) [https://www.ncbi.nlm.nih.gov/nuccore/WWPL00000000], JAAIVG000000000 (Niponia nodulosa) [https://www.ncbi.nlm.nih.gov/nuccore/JAAIVG000000000], JAAFCE000000000 (Trigoniulus corallinus) [https://www.ncbi.nlm.nih.gov/nuccore/JAAFCE000000000], JAHWFP000000000 (Lithobius niger) [https://www.ncbi.nlm.nih.gov/nuccore/JAHWFP000000000], JAHWFO000000000 (Rhysida immarginata) [https://www.ncbi.nlm.nih.gov/nuccore/JAHWFO000000000], and JAFIDM000000000 (Thereuonema tuberculata) [https://www.ncbi.nlm.nih.gov/nuccore/JAFIDM000000000] in NCBI. The raw reads generated in this study were deposited to the NCBI database under the BioProject accessions PRJNA598061 (Glomeris maerens) [https://www.ncbi.nlm.nih.gov/bioproject/PRJNA598061], PRJNA564202 (Helicorthomorpha holstii) [https://www.ncbi.nlm.nih.gov/bioproject/PRJNA564202], PRJNA598060 (Anaulaciulus tonginus) [https://www.ncbi.nlm.nih.gov/bioproject/ PRJNA598060], PRJNA606398 (Niponia nodulosa) [https://www.ncbi.nlm.nih.gov/bioproject/PRJNA606398], PRJNA564195 (Trigoniulus corallinus) [https://www.ncbi.nlm.nih.gov/bioproject/PRJNA564195], PRJNA738717 (Lithobius niger) [https://www.ncbi.nlm.nih.gov/bioproject/PRJNA738717], PRJNA701115 (Rhysida immarginata) [https://www.ncbi.nlm.nih.gov/bioproject/PRJNA701115], and PRJNA699399 (Thereuonema tuberculata) [https://www.ncbi.nlm.nih.gov/bioproject/ PRJNA699399]. The genome annotation files were deposited in the Figshare (https://doi.org/10.6084/m9.figshare.15088722). The scripts for carrying out analyses of this study were deposited in github (https://github.com/xieyichun50/Myriapod-genomes). The databases are available for download from the following websites: eggNOG http://eggnog5.embl.de/download/eggnog_5.0/, GO http://geneontology.org/, KEGG https://www.genome.jp/kegg/pathway.html, and KOG https://www.hsls.pitt.edu/obrc/index.php?page=URL1144075392. Source data are provided with this paper.

Field-specific reporting

Please select the one below that is the best fit for your research. If you are not sure, read the appropriate sections before making your selection.

☒ Life sciences ☐ Behavioural & social sciences ☐ Ecological, evolutionary & environmental sciences

For a reference copy of the document with all sections, see nature.com/documents/nr-reporting-summary-flat.pdf

Life sciences study design

All studies must disclose on these points even when the disclosure is negative.

Sample size

To acvoid sequence polymorphism among individuals of the same species, only one individual of each species was chosen and harvested for genome sequencing of Lithobius niger, Rhysida immarginata, Thereuonema tuberculata, Anaulaciulus tonginus, Glomeris maerens and Niponia nodulosa.

For transcriptome sequencing, one individual was chosen and harvested in Lithobius niger, Rhysida immarginata, Anaulaciulus tonginus,

	Glomeris maerens and Niponia nodulosa. For the transcriptomes of Thereuonema tuberculata, various number of individuals (5 eggs, 5 stadium I, 5 stadium II, 3 stadium III, 1 stadium IV, 1 stadium V, 1 stadium VI and 1 adult) were chosen and pooled for transcriptome sequencing.
Data exclusions	No data were excluded from analysis.
Replication	Genomic and the transcriptomic sequencing were not replicated.
Randomization	Randomization is not applicable to this study.
Blinding	Blinding was not relevant for this study

Reporting for specific materials, systems and methods

We require information from authors about some types of materials, experimental systems and methods used in many studies. Here, indicate whether each material, system or method listed is relevant to your study. If you are not sure if a list item applies to your research, read the appropriate section before selecting a response.

Materials & experimental systems

n/a	Involved in the study	
☒	☐	Antibodies
☒	☐	Eukaryotic cell lines
☒	☐	Palaeontology and archaeology
☐	☒	Animals and other organisms
☒	☐	Human research participants
☒	☐	Clinical data
☒	☐	Dual use research of concern

Methods

n/a	Involved in the study	
☒	☐	ChIP-seq
☒	☐	Flow cytometry
☒	☐	MRI-based neuroimaging

Animals and other organisms

Policy information about studies involving animals; ARRIVE guidelines recommended for reporting animal research

Laboratory animals	This study did not involve laboratory animals.
Wild animals	Adult R. immarginata, L. niger and A. tonginus (10 for each sex) were collected in an agricultural garden of New Asia College, The Chinese University of Hong Kong. Mixed sexes and different developmental stages of 30 adult T. tuberculata, 20 adult G. maerens and 15 adult N. nodulosa were purchased from an exotic pet shop in Hong Kong. Live animals were collected, and each species was contained in a sealed and air-ventilated plastic box during transportation to the laboratory. The remaining animals after the experiment were kept alive in culture under laboratory conditions.
Field-collected samples	Alive adult R. immarginata, L. niger and A. tonginus (10 for each sex) were collected in an agricultural garden of New Asia College, The Chinese University of Hong Kong. The animals were all caught by hand-picking in a soil ground and under flowerpots during the daytime in the summer afternoon. The remaining animals after experiments were kept alive in culture under laboratory conditions.
Ethics oversight	The study did not require an ethical approval.

Note that full information on the approval of the study protocol must also be provided in the manuscript.
